# A tumour-host feed-forward loop contributes to the growth of chromosomal instability-induced tumours

**DOI:** 10.1038/s44319-026-00811-7

**Published:** 2026-06-05

**Authors:** Kaustuv Ghosh, Aishwarya Kunchur, Marco Milán

**Affiliations:** 1 Institute for Research in Biomedicine (IRB Barcelona), The Barcelona Institute of Science and Technology, Baldiri Reixac, 10, 08028 Barcelona, Spain; 2 Institució Catalana de Recerca i Estudis Avançats (ICREA), Pg. Lluís Companys 23, 08010 Barcelona, Spain

**Keywords:** Autophagy & Cell Death, Cancer, Chromatin, Transcription & Genomics

## Abstract

Chromosomal instability (CIN), characterized by frequent changes in chromosome number and structure, is common in human carcinomas and often leads to aneuploidy, an unbalanced number of chromosomes. *Drosophila* has been instrumental in demonstrating that CIN can promote tumour growth and malignancy through aneuploidy-induced senescence, a state marked by cell-cycle arrest and high secretory activity. Despite extensive chromosomal heterogeneity, we show that these cells share a distinct transcriptional programme, with most responses to aneuploidy and senescence regulated at the transcriptional level. We unravel a pro-survival function of the Hippo-Yorkie signalling pathway in aneuploidy-induced senescent cells and present evidence that nearly 10% of the most upregulated genes encode secreted proteins of the senescence-associated secretory phenotype. Five of these proteins act additively, locally or systemically, to block proliferation and induce cell death in neighbouring tissues. This non-autonomous cell death feeds back to the tumour to enhance its growth, resembling super-competition and providing insight into tumour–host interactions relevant to human cancer.

## Synopsis

In Chromosomal Instability-induced epithelial tumours, senescent cells activate the pro-survival Hippo-Yorkie pathway. The Senescence Associated Secretory Phenotype induces the death of neighbouring tissues to feed back to the tumour to enhance its growth.

- In CIN tissues, the cellular responses to aneuploidy and senescence are regulated at the transcriptional level.
- The Hippo-Yorkie pathway plays a pro-survival role in senescent cells.
- The SASP acts systemically through Dilp8/Relaxin and ImpL2/IGFBP7 to suppress tumour growth.
- The SASP acts locally through Eiger/TNF and Upd cytokines to mediate a tumour-host feed-forward loop.

## Abstract (web summary)

In Chromosomal Instability-induced epithelial tumours, senescent cells activate the pro-survival Hippo-Yorkie pathway. The Senescence Associated Secretory Phenotype induces the death of neighbouring tissues to feed back to the tumour to enhance its growth.

## Graphical abstract

**Figure:**
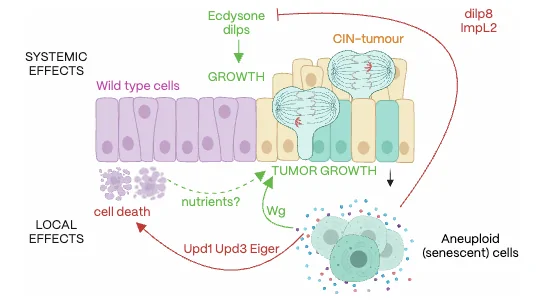

**Figure:**
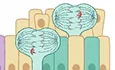

## Introduction

Chromosomal instability (CIN), an increased rate of errors in chromosome segregation during cell division, is one of the most common forms of genomic instability in cancer. It is prevalent in most solid tumours and is closely associated with chemotherapy resistance, reduced patient survival, and metastasis (Nguyen et al, 2022; Lee et al, 2011; van Dijk et al, 2021; Lukow et al, 2021; Ippolito et al, 2021; Davoli et al, 2013). Traditionally, CIN has been understood to be a driver of genetic diversity within tumours, mainly through the gradual gain of chromosomes that carry oncogenes and the loss of those containing tumour suppressor genes. However, beyond these slow, progressive changes, CIN can also cause sudden and dramatic alterations in the genome. For example, mis-segregated chromosomes can end up in micronuclei, where they often experience extensive DNA damage. This process, known as chromothripsis, leads to the fragmentation and chaotic rearrangement of chromosomes (Stephens et al, 2011). In addition to fuelling cancer progression through both gradual and abrupt mechanisms, CIN also causes cytosolic double-stranded DNA, likely originating from ruptured micronuclei. This DNA is detected by cellular sensors, such as cGAS, which normally activate immune responses. However, cancer cells can exploit this pathway to reshape the tumour microenvironment in a way that suppresses immune activity and promotes metastasis (Li et al, 2023, 2021; Bakhoum et al, 2018).

Although low levels of CIN are widely recognized as a driver of cancer, excessive levels can be harmful, leading to cell death and the suppression of tumour growth (Silk et al, 2013; Godek et al, 2016; Weaver et al, 2007; Monserrat et al, 2021). In normal cells, CIN often results in aneuploidy—an abnormal number of chromosomes—which can disrupt cell function (Thompson and Compton, 2010). Studies in yeast and human cells have shown that this imbalance in the number of chromosomes alters protein ratios and interferes with key cellular processes like DNA replication, mitosis, and metabolism (Santaguida and Amon, 2015; Zhu et al, 2018; Li and Zhu, 2022). One outcome of such metabolic disruption is the production of reactive oxygen species (ROS). Studies in *Drosophila* and human epithelial cells subjected to CIN have shown that cells with aneuploid karyotypes that affect a large proportion of the genome typically enter a state of senescence, where they permanently stop dividing and begin releasing inflammatory molecules (Barroso-Vilares et al, 2020; Macedo et al, 2018; Santaguida et al, 2017; Joy et al, 2021). These molecules help the mammalian immune system identify and eliminate abnormal cells (Wang et al, 2021). Research in *Drosophila* has further revealed that aneuploidy-induced senescence promotes tumour development. The fly genome is organized into four chromosomes [two autosomes, a sex chromosome pair (X and Y), and a tiny dot-like autosome]. In epithelial tissues subjected to CIN, random chromosome mis-segregation events generate cells with whole-chromosome aneuploidies (thus impacting a large proportion of the genome) that are expelled from tissues as a result of gene dosage and proteome imbalances, proteostasis failure, and mitochondrial dysfunction (Dekanty et al, 2012; Clemente-Ruiz et al, 2016; Joy et al, 2021). Once extruded, these cells undergo apoptosis as a result of ROS-driven activation of JNK and JAK/STAT signalling pathways (Dekanty et al, 2012; Morais da Silva et al, 2013; Muzzopappa et al, 2017; Joy et al, 2021). When fly aneuploid cells are maintained in the tissue by apoptosis inhibition, they often undergo a JNK-driven transition to a mesenchymal-like state and enter a form of senescence marked by G2 cell cycle arrest (Benhra et al, 2018; Joy et al, 2021). Key molecules expressed by senescent cells include the *Drosophila* orthologs of Wnt and IL-6 [Wingless (Wg) and Upd3, respectively], which act on the epithelial tissue subjected to CIN to drive tumour growth, to enhance cell motility of aneuploidy-induced delaminated cells, and even affect systemic hormonal signals that prevent developmental maturation and cause organismal death (Gracia-Latorre et al, 2022; Dekanty et al, 2012; Romão et al, 2021; Barrio et al, 2023).

In this study, we used the *Drosophila* epithelial model of CIN-induced tumorigenesis to characterize the transcriptional profile of aneuploidy-induced senescent cells. Despite the highly heterogeneous chromosome content of these cells, we unravel a distinct transcriptional programme. This unique transcriptional programme presents evidence that most cellular responses to aneuploidy and senescence, including cell cycle arrest, activation of autophagy, reorganization of the actin cytoskeleton, and a highly secretory phenotype, are regulated at the transcriptional level. In addition to the signalling pathways involved in driving all these cellular responses, we unravel a pro-survival function of the Hippo-Yorkie signalling pathway in aneuploid senescent cells. Besides the secreted proteins well known to promote tumour growth, metastasis, and malignancy (Dekanty et al, 2012; Romão et al, 2021; Barrio et al, 2023; Gracia-Latorre et al, 2022), we uncovered a role of dIlp8 and ImpL2 (fly orthologs of Relaxin and IGBP7, respectively (Garelli et al, 2012; Colombani et al, 2012; Kwon et al, 2015)) in blocking proliferative growth of both wild type and tumour cells, and identified a role for cytokines Upd1 and Upd3 and the *Drosophila* Tumour Necrosis Factor (TNF-α) homologue Eiger in non-autonomously driving cell death in neighbouring wild type cells. Most importantly, we present evidence that this non-autonomous cell death feeds back to the tumour to enhance its growth, thus paralleling the phenomenon of super-competition in cancer, an aggressive form of cell competition where tumour cells actively eliminate neighbouring, healthy wild-type cells to expand its territory (Nagata et al, 2022; Moreno and Basler, 2004; Moreno et al, 2019). Overall, our data highlight potential therapeutic strategies to prevent CIN-driven tumour development by targeting aneuploidy-induced senescent cells and disrupting the relevant interactions with the tumour host.

## Results

### Cellular responses to aneuploidy and senescence are reflected at the transcriptional level

**Figure 1:**
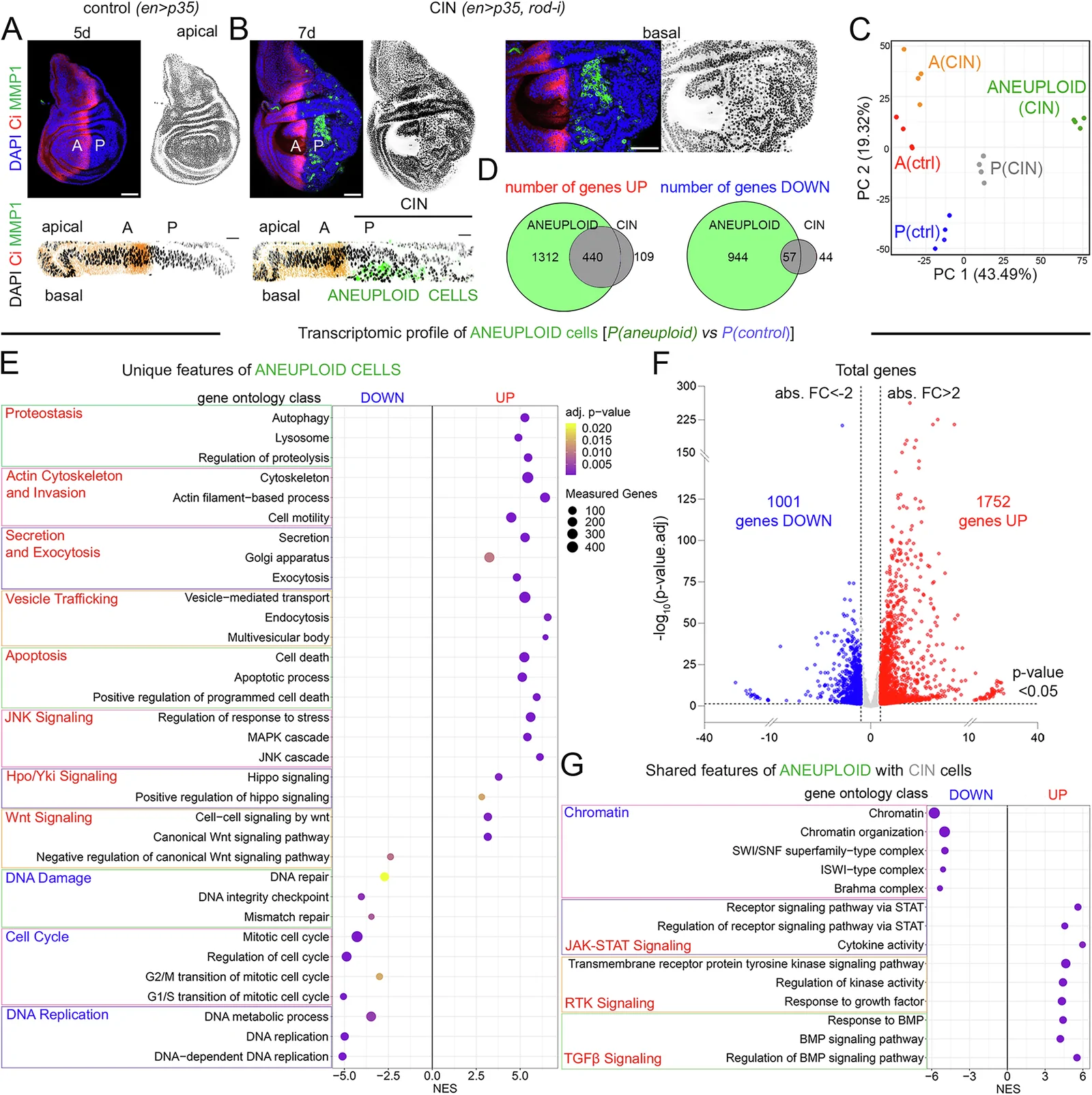
A distinct transcriptomic profile of aneuploidy-induced senescent cells. (**A**,** B**) Control wing discs (**A**) and wing discs subjected to CIN (**B**), stained for DAPI (blue or black), Ci (red, to label the A compartment), and MMP1 (green, to label the aneuploid cells) and expressing the indicated transgenes in the P compartment under the control of the *en-gal4* driver. Scale bars, 50 µm. Days of dissection after egg laying are as indicated. (**C**) Principal Component (PC) analysis showing the transcriptomic differences between the 5 different populations: A (Red) and P (Blue) cells of control wing discs, and aneuploid (green), A (orange) and P (grey) cells of wing discs subjected to CIN. (**D**) Venn diagram representing the number of genes up- or down-regulated uniquely in aneuploid cells (green), and proliferating cells subjected to CIN cells or in both types of cells (grey). (**E**) Bubble plot representing Gene Ontology (GO) enrichment analyses unique to aneuploid cells when compared to P cells of control discs. Size of the bubble represents the number of measured genes, and the colour scale represents the associated *p*-value. (**F**) Volcano plot representing all the genes that are differentially up- (red dots, FC > 2 and *p*-value < 0.05) or down-regulated (blue dots FC < −2 and *p*-value < 0.05), or not differentially expressed (grey dots, *p*-value > 0.05 or 2 > FC > −2) in aneuploid cells when compared to P cells of control discs. Fold changes and *p*-values were calculated using the DESeq2 R package. *p*-values were adjusted for multiple comparisons with the Benjamini-Hochberg algorithm. (**G**) Bubble plot representing Gene Ontology (GO) enrichment analyses shared between aneuploid and proliferating cells when compared to P cells of control discs. In (**E**, **G**), statistics from the roastgsa enrichment algorithm of selected pathways are shown, where the X axis corresponds to the normalized enrichment score (NES, see the methods section for details on methodology), size of the bubble represents the number of measured genes and the colour scale represents the associated *p*-value. In (**E**–**G**), *n* (number of technical replicates per genotype) = 4. See also Figs. EV1, EV2, and Dataset EV1. Scale bars, 10 µm. Source data are available online for this figure.

**Figure EV1:**
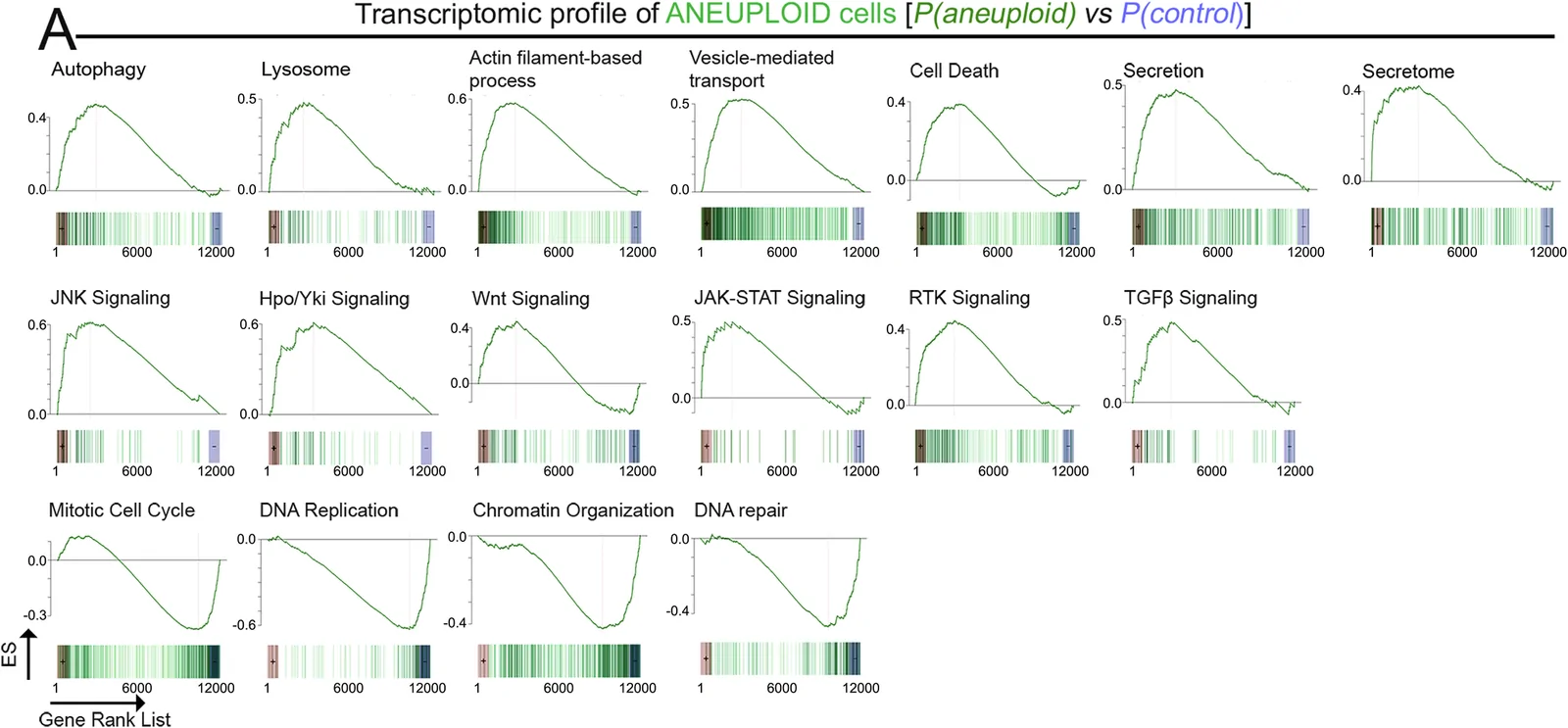
Transcriptomic landscape of aneuploidy-induced senescent cells (related to Fig. 1). (**A**) Mountain plots representing Enrichment Score (ES) upon Gene Set Enrichment Analysis (GSEA) comparing P(Aneuploid) with P(control) cells. Top row - up-regulated cellular processes: Autophagy - GO0006914, Lysosome - GO0005764, Actin filament-based process - GO0030029, Vesicle-mediated transport - GO0016192, Cell death - GO0008219, Secretion - GO0046903, Secretome - Custom Gene Set. Middle row - up-regulated signalling pathways: JNK Signalling - GO0007254, Hpo/Yki Signalling - GO0035329, Wnt Signalling - GO0198738, JAK-STAT Signalling - GO0097696, RTK Signalling - GO0007169, TGFβ Signalling - GO0030509. Bottom row - down-regulated cellular processes: Mitotic cell cycle - GO0000278, DNA Replication - GO0006260, Chromatin Organization - GO0006325, DNA repair - GO0006281. See also Dataset EV1.

**Figure 2:**
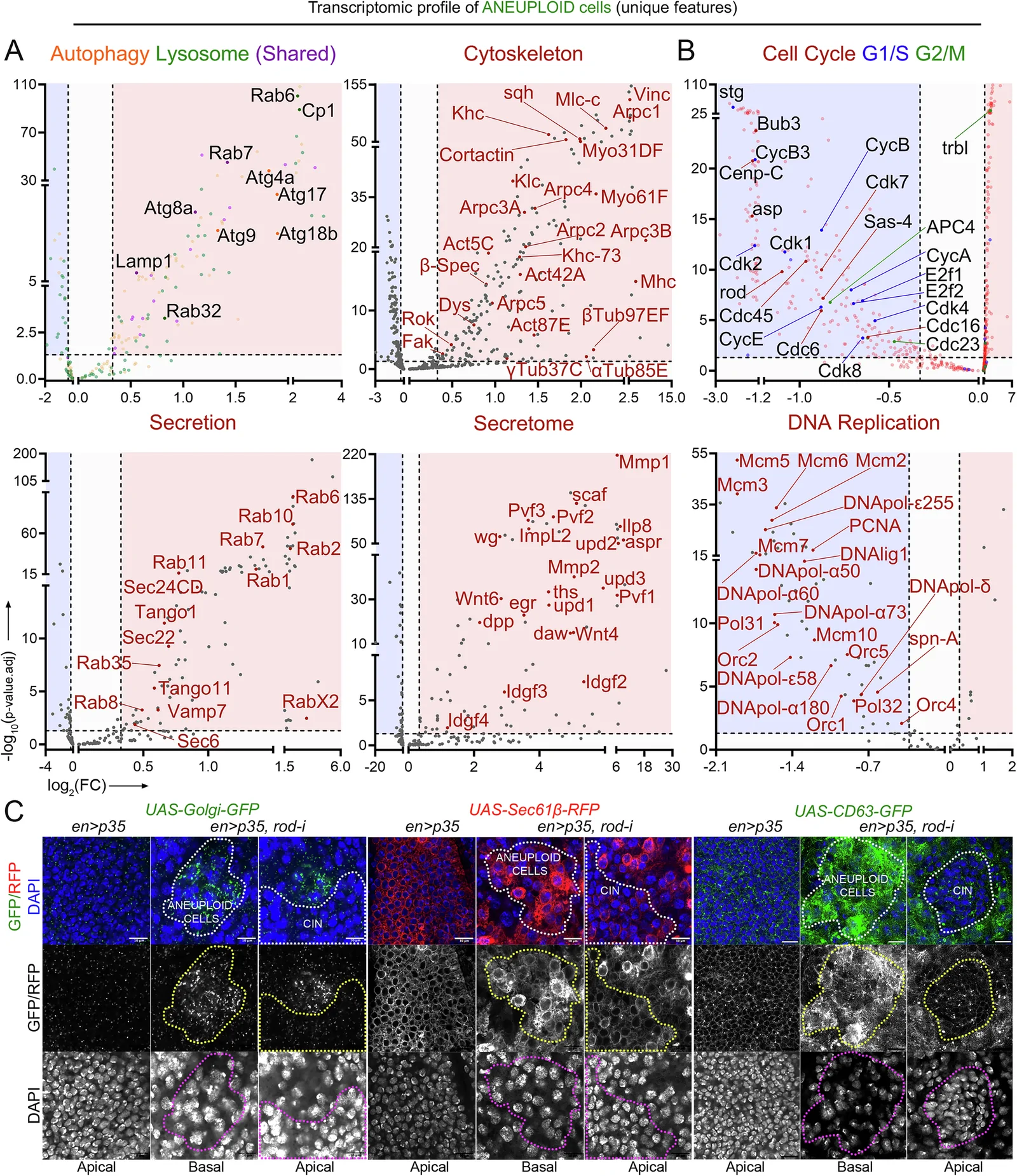
The transcriptome of aneuploidy-induced senescent cells points to a heightened secretory potential. (**A**, **B**) Volcano plots representing GO class-specific genes that are differentially upregulated (red area, FC > 2 and *p*-value < 0.05), downregulated (blue area, FC < −2 and *p*-value < 0.05), or not differentially expressed (white area, *p*-value > 0.05 or 2 > FC > −2) in aneuploid cells when compared to P cells of control discs. GO classes (**A**): Autophagy - GO0006914, Lysosome - GO0005764, Cytoskeleton - GO0005856, Secretion - GO0046903, Secretome - Custom Gene Set. GO classes (**B**): G1/S Transition - GO0000082, G2/M Transition - GO0000086, Regulation of cell cycle - GO0051726, and DNA Replication - GO0006260. Genes corresponding to each of the GO classes have a distinct colour code. Fold changes and *p*-values were calculated using the DESeq2 R package. *p*-values were adjusted for multiple comparisons with the Benjamini-Hochberg algorithm. *n* (number of technical replicates per genotype) = 4. (**C**) High magnification images of wing discs expressing the indicated transgenes under the control of the *en-gal4* driver and stained to visualize differential expression of fluorescent reporters (green, red, or white) and DAPI (blue and white). Apical and basal sections are shown. Aneuploid and CIN cells are marked by a dashed line. Scale bars, 10 µm. See also Dataset EV1 and EV2. Source data are available online for this figure.

**Figure EV2:**
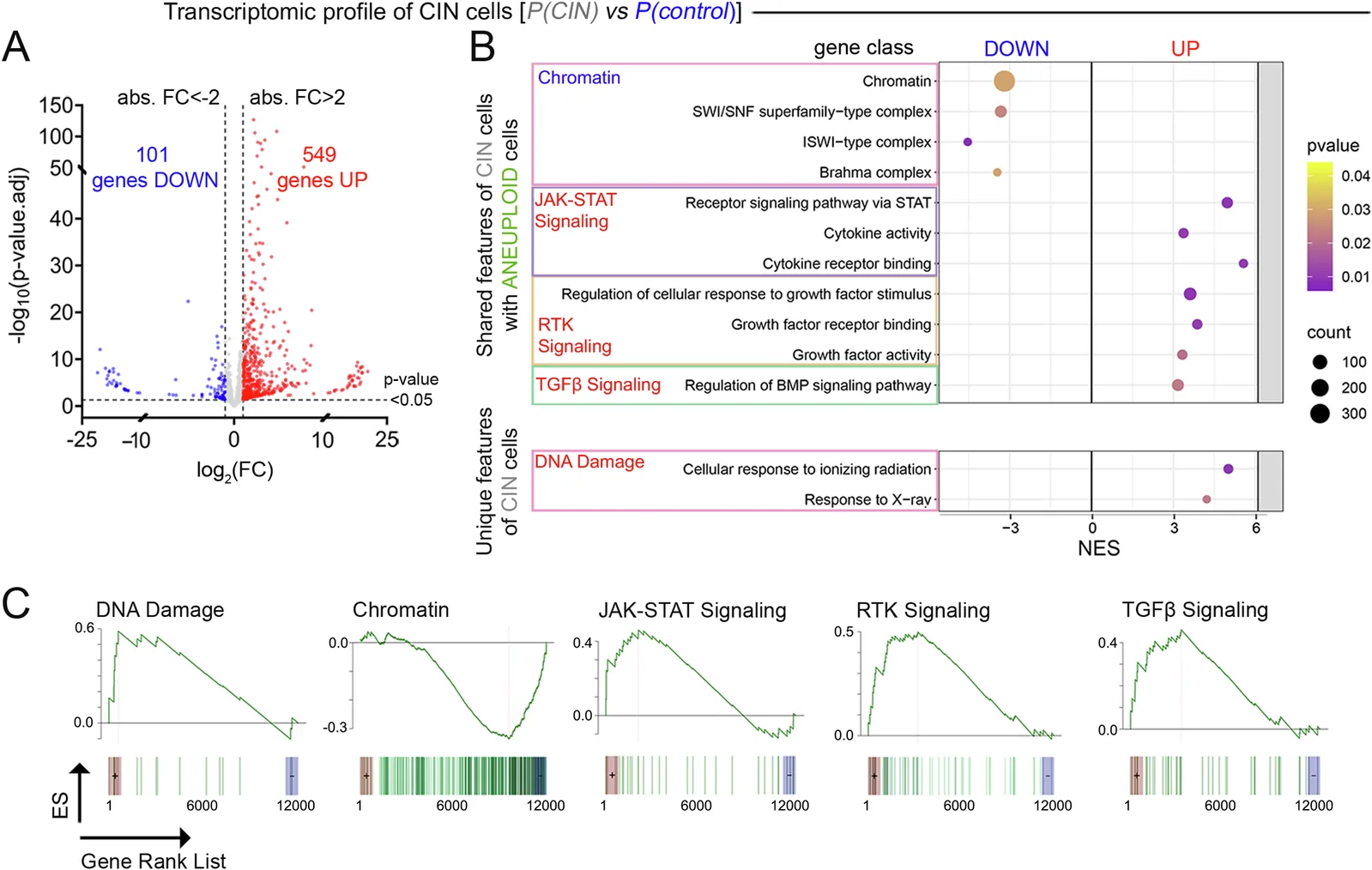
Transcriptomic landscape of proliferating epithelium subjected to CIN (related to Fig. 1). (**A**) Volcano plot representing all genes that are differentially up- (red dots, FC > 2 and *p*-value < 0.05) or down-regulated (blue dots, FC < −2 and *p*-value < 0.05), or not differentially expressed (grey dots, *p*-value > 0.05 or 2 > FC > −2) in proliferating cells subjected to CIN when compared to P cells of control discs. Fold changes and *p*-values were calculated using the DESeq2 R package. *p*-values were adjusted for multiple comparisons with the Benjamini-Hochberg algorithm. (**B**) Bubble plot representing Gene Ontology (GO) enrichment analyses of proliferating epithelium subjected to CIN when compared to P cells of control discs. On the top, GOs shared with aneuploid cells. On the bottom, those GOs uniquely upregulated in the proliferating epithelium subjected to CIN. Statistics from the roastgsa enrichment algorithm of selected pathways are shown, where X axis corresponds to the normalized enrichment score (NES), size of the bubble represents the number of measured genes and colour-scale represents the associated *p*-value. See the methods section for details on methodology. (**C**) Mountain plots representing Enrichment Score (ES) upon Gene Set Enrichment Analysis (GSEA) of proliferating cells subjected to CIN when compared to P cells of control discs. GO classes: DNA Damage - GO0071479, Chromatin - GO0000785, JAK-STAT Signalling - GO0097696, RTK Signalling - GO0090287, TGFβ Signalling - GO0030510. *n* (number of technical replicates per genotype) = 4. See also Dataset EV1.

We used the wing primordium of *Drosophila*—a highly proliferative epithelial monolayer that forms a two-sided epithelial sac (Fig. 1A)—as a model system to characterize the transcriptional profile of cells subjected to CIN. CIN, an increased rate of changes in chromosome structure and number, was induced by depletion of the spindle assembly checkpoint (SAC) gene *rod* (*rough deal*) using an RNAi form expressed with the GAL4/UAS system (Dekanty et al, 2012; Morais da Silva et al, 2013). The *engrailed-gal4* driver, which is restricted to the posterior (P) compartment of the wing primordium (Fig. 1A), was used to limit the induction of CIN to a defined cell population. One of the most attractive features of this model of CIN is that it facilitates the identification of three well-defined cell populations with distinct behaviours (Fig. 1B). On one hand, if apoptosis is blocked by the expression of p35 (a baculovirus protein that binds and represses effector caspases Dcp-1 and Drice (Hay et al, 1994)), those cells that become aneuploid—as a consequence of random chromosome missegregation events in mitosis caused by *rod* depletion—delaminate and become cell cycle-arrested, motile, and highly secretory. These cells can then be tracked with MMP1-GFP, which is a GFP-based transcriptional reporter of JNK (Uhlirova and Bohmann, 2006) (Fig. 1B, green cells). On the other hand, the epithelial tissue subjected to CIN (the P compartment), which consists of cells that remain largely euploid, enters a hyperplastic programme that relies on high mitotic activity and unlimited growth potential (Fig. 1B, cells located apically to the green cells). Finally, the anterior (A) compartment of these wing primordia (Fig. 1B, cells labelled by the Ci marker in red), which are not subject to CIN and whose cells are euploid, will be treated as the neighbouring cell population. The transcriptional profile of these three cell populations (CIN, aneuploid cells, and neighbouring cell population) was compared to the transcriptional profile of control wing primordia expressing p35 under the control of the *engrailed-gal4* driver. Four distinct biological replicates were analysed. Principal Component Analysis (PCA) revealed significant transcriptomic differences between the five populations, showing strict clustering based on their respective traits (Fig. 1C). Interestingly, the aneuploid cell population cluster presented the highest level of difference when compared to the compartment of origin [P(CIN)] or the control tissue [P(Control)]. Consistent with this observation, the aneuploid population showed a higher number of upregulated and downregulated genes than the CIN epithelium (Fig. 1D,F, see also Fig. EV2A). However, there was a certain degree of overlap in the type of genes that changed in these two populations when compared to control tissues. We thus analysed the transcriptional profile that was unique to aneuploid cells. Remarkably, many of the previously observed cellular responses to aneuploidy were reflected at the transcriptional level, as shown by gene set enrichment analysis (GSEA, Figs. 1E and EV1A; Dataset EV1). First, high levels of proteotoxic stress caused by unbalanced karyotypes were reflected by strong upregulation of genes involved in autophagy (e.g., *atg4a*, *atg8a*, *atg9*, *atg17*, and *atg18b*) and lysosome function (e.g., *Lamp1*, *Vps13D*, *Vps16A, Vamp7*, and *lqf*). Second, consistent with the activation of the JNK stress signalling pathway as a consequence of mitochondrial dysfunction and ROS (Muzzopappa et al, 2017; Joy et al, 2021), many of its target genes (e.g., *mmp1*, *ets21C*, and *scaf*) were strongly upregulated in aneuploid cells (Figs. 1E, 2A and EV1A). Third, the ability of aneuploid cells to become motile through modulation of the actin cytoskeleton and a non-apoptotic role of caspases (Benhra et al, 2018; Barrio et al, 2023) was reflected by strong upregulation of genes involved in these two processes, as well as in cell motility (e.g., *sqh*, *cortactin*, *rok*, *fak*, *Arpc2-5, hid*, *rpr*, *Dronc*, *Drice*, and *Dcp-1;* Figs. 1E, 2A and EV1A). Finally, entry into a senescence-like state ^1^ was also reflected at the transcriptional level, as shown by strong downregulation of genes involved in cell cycle progression (e.g., *stg*, *cdk1*, *cycA*, and *cycB*) and DNA replication (e.g., *DNA polymerase*, *Orc* and *Mcm* complexes, Figs. 1E and 2B), and the clear upregulation of genes involved in secretion (e.g., *Tango 5, 11, Sec 5, 6, 33, 24CD*, and *Rab 1, 2, 6, 7, 10*, and *35*) and encoding for secreted proteins (Figs. 1E, 2A, and Dataset EV2). Indeed, 10% of the most upregulated genes encoded for secreted proteins (Table 1). These included the systemic signals Upd3 cytokine (involved in CIN-induced invasion and developmental delay (Romão et al, 2021; Barrio et al, 2023)), Dilp8 relaxin (mediating developmental delay (Romão et al, 2021; Barrio et al, 2023)), Pvf1 (PDGF- and VEGF-related factor 1 involved in driving organ wasting (Song et al, 2019)) and the mitogenic signals Wg and Wnt6 (Gracia-Latorre et al, 2022), many of them are known targets of JNK. We confirmed the highly secretory phenotype of senescent cells by using three fluorescent reporters to mark the enlargement of the Golgi and the endoplasmic reticulum (ER) compartment, and an increase in the number of exosomes (Fig. 2C). The transcriptional profile of aneuploid cells that was shared with CIN comprised genes involved in the activation of ERK and JAK/STAT signalling, previously shown to contribute to various aspects of CIN-induced tumorigenesis, such as growth and invasiveness (Benhra et al, 2018; Barrio et al, 2023) (Figs. 1G and EV2B,C). In CIN cells, we observed unique upregulation of the genes involved in DNA damage, consistent with the high propensity of dividing cells subjected to SAC depletion to accumulate lagging or broken chromosomes (Fig. EV2B,C). Overall, these results indicate that the cellular responses to aneuploidy and senescence are reflected at the transcriptional level, and they reinforce the highly secretory phenotype of aneuploidy-induced senescent cells.

**Table 1:** The secretome of aneuploidy-induced senescent cells.

Symbol	Gene name	Vertebrate homolog	Significance (*p* value)	Fold change
RYa	Ryamide	NPY family	3.25E−08	21721233,75
NLaz	Neural Lazarillo	ApoD and RBP4	1.07E−35	681,212
aspr	Asperous	DNER and EDIL3	1.32E−55	491,381
Ilp8	Insulin-like peptide 8	Relaxin	5.46E−81	198,57
upd2	Unpaired 2	Leptin	3.26E−49	110,948
nec	Necrotic	SERPIN	1.02E−75	89,581
PGRP-SA	Peptidoglycan recognition protein SA	PGLYRP3	2.88E−59	88,703
Mmp1	Matrix metalloproteinase 1	MMP	2.30E−215	83,782
Pvf1	PDGF- and VEGF-related factor 1	PDGF and VEGF	4.28E−31	75,409
Tig	Tiggrin	RGD-containing integrin ligand	1.96E−26	73,827
Sp7	Serine protease 7	Serine Proteases	5.87E−85	65,623
upd3	Unpaired 3	Il-6	3.28E−34	56,97
CG30280	Unannotated	MFAP4	8.16E−05	43,886
sona	sol narae	ADAMTS	2.08E−120	41,327
tnc	tenectin	Mucin-type protein with RGD motif	4.07E−17	37,48
scaf	scarface	Serine Protease	2.42E−124	34,526
Mmp2	Matrix metalloproteinase 2	MMP	3.28E−39	31,565
frm	farmer	FLG	1.72E−144	30,547
Wnt4	Wnt oncogene analog 4	WNT	1.01E−14	28,179
Idgf2	Imaginal disc growth factor 2	CHI3L1, CHI3L2, and OVGP1	5.07E−07	26,81
daw	dawdle	NODAL	1.84E−14	26,357
Spn47C	Serpin 47C	SERPIN	4.32E−71	23,661
Pvf2	PDGF- and VEGF-related factor 2	PDGF and VEGF	5.62E−99	21,107
ths	thisbe	FGF8	1.46E−32	18,374
CG31869	Unannotated	KCP	2.31E−86	18,285
upd1	unpaired 1	Il-6	1.01E−26	18,278
mtg	mind the gap	Lectin	3.46E−90	13,673
ImpL2	Ecdysone-inducible gene L2	IGFBP7	1.43E−76	12,527
Pvf3	PDGF- and VEGF-related factor 3	PDGF and VEGF	1.21E−92	12,395
CecB	Cecropin B	Similar to Antimicrobial peptides	1.15E−03	12,284
spz	spatzle	Neurotrophin	7.22E−09	11,326
CG9095	Unannotated	Secretory phospholipase A2	3.11E−19	10,982
egr	eiger	TNF	2.52E−22	10,773
fon	fondue	NCOA5 and NUP98	3.01E−08	9,755
l(1)G0193	orion	Similar to Chemokines	2.25E−56	8,84
Dro	Drosocin	Similar to Antimicrobial peptides	1.43E−03	8,007
Spn55B	Serpin 55B	SERPIN	1.94E−64	7,591
Wnt6	Wnt oncogene analog 6	WNT	1.32E−29	6,846
wg	wingless	WNT	5.12E−62	6,798
Cp15	Chorion protein 15	No homolog identified	4.48E−04	6,417

Table representing the 40 most differentially upregulated genes (comparing the transcriptome of the aneuploidy-induced senescent cells with control cells) which are annotated as genes encoding for secreted proteins, along with their vertebrate homolog, adjusted *p* values and shrunken fold-changes. The genes labelled in red are those which have been mentioned in the current work. A manually curated custom gene set, consisting of 261 known or predicted secreted proteins as inferred from FlyBase is provided in Dataset EV2. The transcriptome of the aneuploidy-induced senescent cells was compared to posterior wild-type cells.

### A survival role of hippo/Yorkie in CIN-induced senescent cells

**Figure 3:**
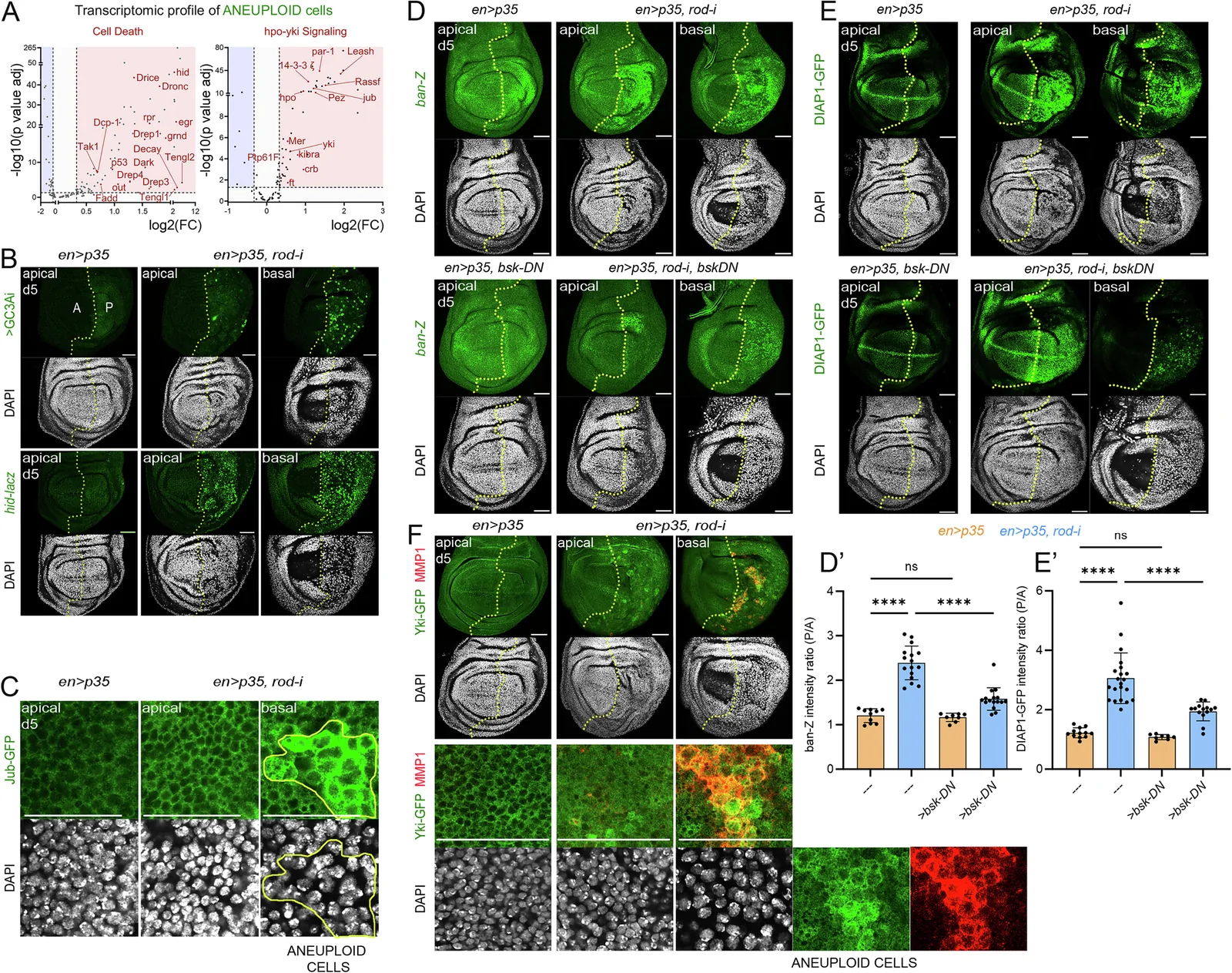
The transcriptome of aneuploidy-induced senescent cells points to activation of the apoptotic and Yorkie pathways. (**A**) Volcano plot representing selected genes in ‘Cell death - GO0008219’ (left), and ‘hpo-yki signalling - GO0035329’ (right) classes that are differentially upregulated (red area, FC > 1.3 and *p*-value < 0.05), downregulated (blue area, FC < −1.3 and *p*-value < 0.05), or not differentially expressed (white area, *p*-value > 0.05 or 1.3 > FC > −1.3) in aneuploid cells when compared to P cells of control discs. Fold changes and *p*-values were calculated using the DESeq2 R package. *p*-values were adjusted for multiple comparisons with the Benjamini-Hochberg algorithm*. n* (number of technical replicates per genotype) = 4. (**B**–**F**) Control wing discs (first column) and wing discs subjected to CIN (remaining columns), stained to visualize GC3Ai or *hid-lacZ* (green, **B**), Jub-GFP (green, **C**), *bantam-lacZ* (green, **D**), DIAP1-GFP (green, **E**), Yorkie-GFP (green, **F**), DAPI (white, **B**–**F**), MMP1 (red, **F**), and expressing the indicated transgenes in the P compartment under the control of the *en-gal4* driver. In (**C**), aneuploidy-induced senescent cells are marked by a yellow line. Scale bars, 50 µm. Discs were dissected 5 days AEL. The boundary between A and P cells is labelled by a yellow line. Note in (**D**) and (**E**), a reduction in the expression levels of *bantam-lacZ* and *DIAP1-GFP* upon expression of a dominant negative form of JNK (Bsk-DN). (**D’**, **E’**) Histograms plotting the P/A signal intensity ratio of *bantam-lacZ* (**D’**) and *DIAP1-GFP* (**E’**) of wing discs expressing the indicated transgenes in the P compartment under the control of the *en-gal4* driver. Graphs represent mean and standard deviation. *p < 0.05, **p < 0.01, ***p < 0.001, ****p < 0.0001, ns, not significant. One-way ANOVA with Dunnett’s multiple comparison test, n=number of biological replicates. Sample size and *P* value: (**D’**): *en* > *p35* (*n* = 10) vs *en* > *p35, rod-i* (*n* = 16) (*p* = 8.17 × 10^−10^); *en* > *p35* (*n* = 10) vs *en* > *p35, bsk-DN* (*n* = 8) (*p* = 0.979); and *en* > *p35, rod-i* (*n* = 16) vs *en* > *p35,rod-i, bsk-DN* (*n* = 16) (*p* = 8.22 × 10^−7^); (**E’**): *en* > *p35* (*n* = 12) vs *en* > *p35, rod-i* (*n* = 21) (*p* = 5.41 × 10^−9^); *en* > *p35* (*n* = 12) vs *en* > *p35, bsk-DN* (*n* = 8) (*p* = 6.69 × 10^−2^); and *en* > *p35, rod-i* (*n* = 21) vs *en* > *p35, rod-i, bsk-DN* (*n* = 14) (*p* = 2.75 × 10^−5^), See also Dataset EV3. See also Fig. EV3, and Dataset EV1 and EV3. Source data are available online for this figure.

**Figure 4:**
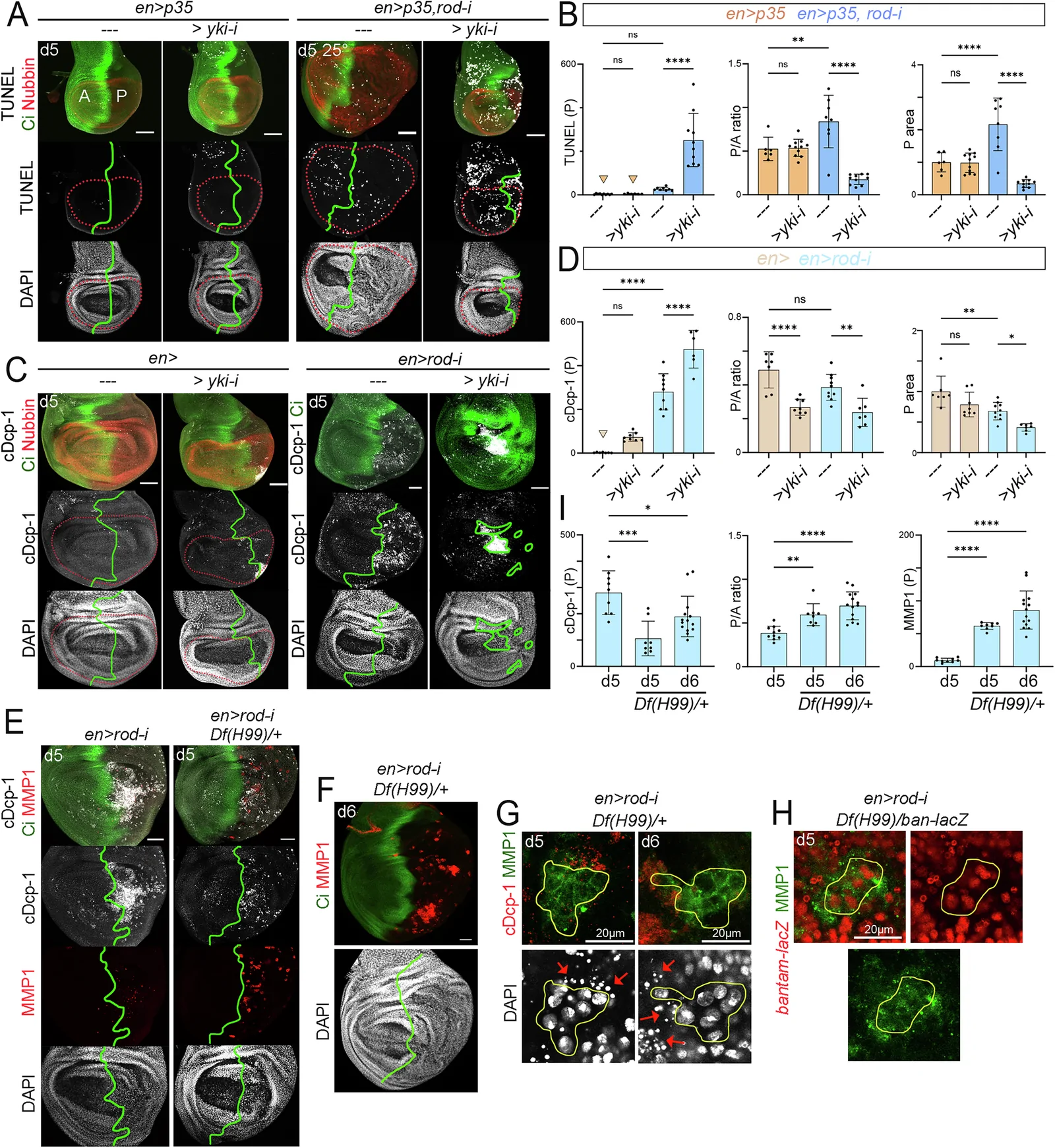
Yorkie plays a pro-survival role in CIN tissues. (**A**) Control wing discs (first columns) and wing discs subjected to CIN (remaining columns), stained to visualize TUNEL (white), Ci (green), Nubbin (red), and DAPI (white) expressing the indicated transgenes in the P compartment under the control of the *en-gal4* driver. (**B**, **D**, **I**) Histograms plotting the amount of TUNEL (**B**, left), MMP1 (**I**, right) and cDcp-1 signal (**D**, and **I**, left) in the P wing pouch compartment (normalized to the size of the corresponding compartment), the normalized size of the P wing pouch compartment (**B**, **D**, right), and size ratio between the P and A wing pouch compartments (**B**,** D**, **I**, centre) of wing discs expressing the indicated transgenes in the P compartment under the control of the *en-gal4* driver. Graphs represent mean and standard deviation. *p < 0.05, **p < 0.01, ***p < 0.001, ****p < 0.0001, ns, not significant. One-way ANOVA with Šídák’s multiple comparisons test (**B**, **D**), one-way ANOVA with Dunnett’s multiple comparison test (**I**), n=number of biological replicates. Sample size and *p* value: (**B**, TUNEL): *en* > *p35* (*n* = 10) vs *en* > *p35, yki-i* (*n* = 6) (*p* = 0.99); *en* > *p35* (*n* = 10) vs *en* > *p35, rod-i* (*n* = 8) (*p* = 0.84); and *en* > *p35, rod-i* (*n* = 8) vs *en* > *p35, rod-i, yki-i* (*n* = 10) (*p* = 2.35 × 10^−7^); (**B**, P/A ratio): *en* > *p35* (*n* = 6) vs *en* > *p35, yki-i* (*n* = 11) (*p* = 0.998); *en* > *p35* (*n* = 6) vs *en* > *p35, rod-i* (*n* = 8) (*p* = 4.26 × 10^−3^); and *en* > *p35, rod-i* (*n* = 8) vs *en* > *p35, rod-i, yki-i* (*n* = 10) (*p* = 4.98 × 10^−9^); (**B**, P area): *en* > *p35* (*n* = 6) vs *en* > *p35, yki-i* (*n* = 11) (*p* = 1); *en* > *p35* (*n* = 6) vs *en* > *p35, rod-i* (*n* = 8) (*p* = 8.60 × 10^−5^); and *en* > *p35, rod-i* (*n* = 8) vs *en* > *p35, rod-i, yki-i* (*n* = 10) (*p* = 2.43 × 10^−9^); (**D**, cDcp1 (P)): *en* > (*n* = 7) vs *en> yki-i* (*n* = 8) (*p* = 0.09); *en* > (*n* = 7) vs *en>rod-i* (*n* = 9) (*p* = 3.97 × 10^−9^); and *en> rod-i* (*n* = 9) vs *en>rod-i, yki-i* (*n* = 6) (*p* = 5.53 × 10^−6^); (**D**, P/A ratio): *en* > (*n* = 7) vs *en> yki-i* (*n* = 8) (*p* = 3.54 × 10^−5^); *en* > (*n* = 7) vs *en> rod-i* (*n* = 10) (*p* = 0.04); and *en> rod-i* (*n* = 10) vs *en> rod-i, yki-i* (*n* = 7) (*p* = 2.60 × 10^−3^); (**D**, P area): *en* > (*n* = 7) vs *en> yki-i* (*n* = 8) (*p* = 0.08); *en* > (*n* = 7) vs *en> rod-i* (*n* = 10) (*p* = 2.89 × 10^−3^); and *en> rod-i* (*n* = 10) vs *en> rod-i, yki-i* (*n* = 7) (*p* = 0.015); (**I**, cDcp1 (P)): *en>rod-i* d5 (*n* = 9) vs *en> rod-i; Df(H99)/+* d5 (*n* = 8) (*p* = 1.20 × 10^−4^); and *en> rod-i; Df(H99)/+* d5 (*n* = 9) vs *en> rod-i*; *Df(H99)/+* d6 (*n* = 14) (*p* = 0.017); (**I**, P/A ratio): *en>rod-i* d5 (*n* = 10) vs *en> rod-i; Df(H99)/+* d5 (*n* = 8) (*p* = 3.98 × 10^−3^); and *en> rod-i; Df(H99)/+* d5 (*n* = 10) vs *en> rod-i; Df(H99)/+* d6 (*n* = 14) (*p* = 6.18 × 10^−6^); (**I**, MMP1): *en>rod-i* d5 (*n* = 9) vs *en> rod-i; Df(H99)/+* d5 (*n* = 8) (*p* = 2.13 × 10^−5^); and *en> rod-i; Df(H99)/+* d5 (*n* = 9) vs *en> rod-i; Df(H99)/+* d6 (*n* = 14) (*p* = 2.33 × 10^−9^). (**C**,** E**,** F**,** G**,** H**) Control wing discs (first columns in **C**) and wing discs subjected to CIN (remaining columns), stained to visualize cDcp-1 (**C**, **E**, white, **G**, red), *bantam-lacZ* (red, **H**), Ci (green, **C**, **E**,** F**), Nubbin (red, **C**), MMP1 (red, **E**, green, **G**, **H**), and DAPI (white, **C**, **E**, **F**, **G**) expressing the indicated transgenes in the P compartment under the control of the *en-gal4* driver. In (**A**, **C**,** E**, **F**,** G**,** H**), scale bars, 50 µm, unless indicated otherwise, discs were dissected at the indicated days of development, the boundary between A and P cells is labelled by a green line, and the wing pouch by a red dotted line. (**G**) and (**H**) show high magnification images of the basal sections of CIN tissues, where aneuploidy-induced senescent cells are found and marked by a yellow line. Red arrows in (**G**) point to dying cells. See also Dataset EV3. Source data are available online for this figure.

In CIN-induced tumours, the ability of aneuploid cells to become motile relies on modulation of the actin cytoskeleton and a non-apoptotic role of caspases (Benhra et al, 2018; Barrio et al, 2023). Consistent with this, activation of the apoptotic pathway in CIN tissues (monitored with cDcp1 antibodies, Fig. 3D,E) was not followed by apoptosis execution (monitored by TUNEL staining, Fig. 3C). Activation of the apoptotic pathway in aneuploidy-induced senescent cells was reflected by strong upregulation of genes involved in apoptosis, including the pro-apoptotic genes *hid* and *reaper*, the initiator caspase *Dronc*, and the effector caspases *Drice* and *Dcp-1* (Fig. 3A). The increase in *hid* expression was confirmed by a *hid-lacZ* reporter (Fig. 3B). Interestingly, the activity levels of effector Caspases, monitored by the GC3Ai activity reporter (Schott et al, 2017), were relatively high in aneuploidy-induced senescent cells when compared to control tissues (Fig. 3B) despite the expression of the p35 baculovirus protein, a potent inhibitor of these Caspases (Hay et al, 1994). However, there were no pyknotic nuclei in the tissue suggesting that apoptosis execution is indeed actively blocked in these cells. In our search for candidate pathways with well-known pro-survival activity, we observed that many elements of the Hippo/Yorkie signalling cascade, including *crumbs* (*crb*), *fat* (*ft*), *Merlin* (*Mer*), *Ajuba* (*jub*), *kibra*, *hippo* (*hpo*), and *yorkie* (*yki*), were transcriptionally activated in aneuploidy-induced senescent cells (Fig. 3A). The activation of Yorkie in these cells was confirmed with the use of GFP-protein traps of Ajuba (Jub-GFP, an upstream negative regulator of Hippo (Das Thakur et al, 2010)) and Yorkie (Fig. 3C,F), transcriptional reporter and GFP-protein trap of *Diap1* (an E3 ubiquitin ligase regulated by Yorkie and involved in inhibiting Dronc, (Meier et al, 2000; Tenev et al, 2005), Figs. 3E and EV3), and transcriptional reporters of *bantam* (a miRNA regulated by Yorkie and involved in inhibiting *hid* (Thompson and Cohen, 2006; Nolo et al, 2006)), and *expanded* [an upstream regulator of the Hippo pathway and Yorkie target (Hamaratoglu et al, 2006), Figs. 3D and EV3]. Yorkie has been previously shown to be activated by JNK (Sun and Irvine, 2013), which is highly active in CIN-induced aneuploid cells and plays a pivotal role in defining their senescent state (Joy et al, 2021). Consistently, the expression levels of Yorkie targets *Diap1* and *bantam* were significantly reduced upon blockage of the JNK pathway by expression of a dominant negative version of JNK (Bsk in *Drosophila* (Sluss et al, 1996), Fig. 3D,E, quantified in Fig. 3D’,E’). Given the well-known anti-apoptotic role of the Yorkie pathway, we addressed whether Yorkie depletion had any impact on the survival of CIN tissues. To this end, and based on the developmental role of Yorkie in regulating growth and survival of epithelial tissues (Kowalczyk et al, 2022), we searched for a mild *UAS-yorkie-RNAi* transgene. Interestingly, depletion of Yorkie (with the use of this mild transgene) did not have a major effect on the size or survival of p35-overexpressing tissues but did have a mild effect on the size and survival of otherwise wild-type tissues (Fig. 4A,C, left panels, and quantified in Fig. 4B,D). In contrast, the use of this transgene induced a strong negative impact on the size of CIN tissue, and this reduction was accompanied by a significant increase in the number of apoptotic cells (Fig. 4A,C, right panels, and quantified in Fig. 4B,D). In experimental conditions where apoptosis was not blocked, the CIN tissue collapsed and was transformed into isolated islands of dying cell populations (Fig. 4C, right panel). Taken together, these results indicate that the Hippo/Yorkie pathway acts as an intrinsic anti-apoptotic pathway that contributes to dampening the deleterious effects of CIN and maintaining senescent cells alive despite the activation of the apoptotic pathway. Consistent with the proposal of this intrinsic anti-apoptotic pathway, we observed that by halving the doses of the pro-apoptotic genes *reaper*, *hid* and *grim* (in *Df(H99)/+* larvae), the levels of cell death observed in CIN tissues were reduced (Fig. 4E, and quantified in Fig. 4I), and aneuploid cells, labelled by the expression of MMP1, were maintained in the tissue even in the absence of p35 (Fig. 4E,F). These cells were negative for apoptotic markers (Fig. 4G) and were not the result of loss of Df(H99) heterozygosity caused by CIN, as they expressed the *bantam-lacZ* reporter located in the homologous chromosome (Fig. 4H). Thus, aneuploid cells were not maintained in the tissue in this experimental setting as a trivial consequence of inheriting the two chromosomes bearing the *Df(H99)* deficiency. Most interestingly, under these circumstances, the tissue also overgrew (Fig. 4E,F, and quantified in Fig. 4I).

**Figure EV3:**
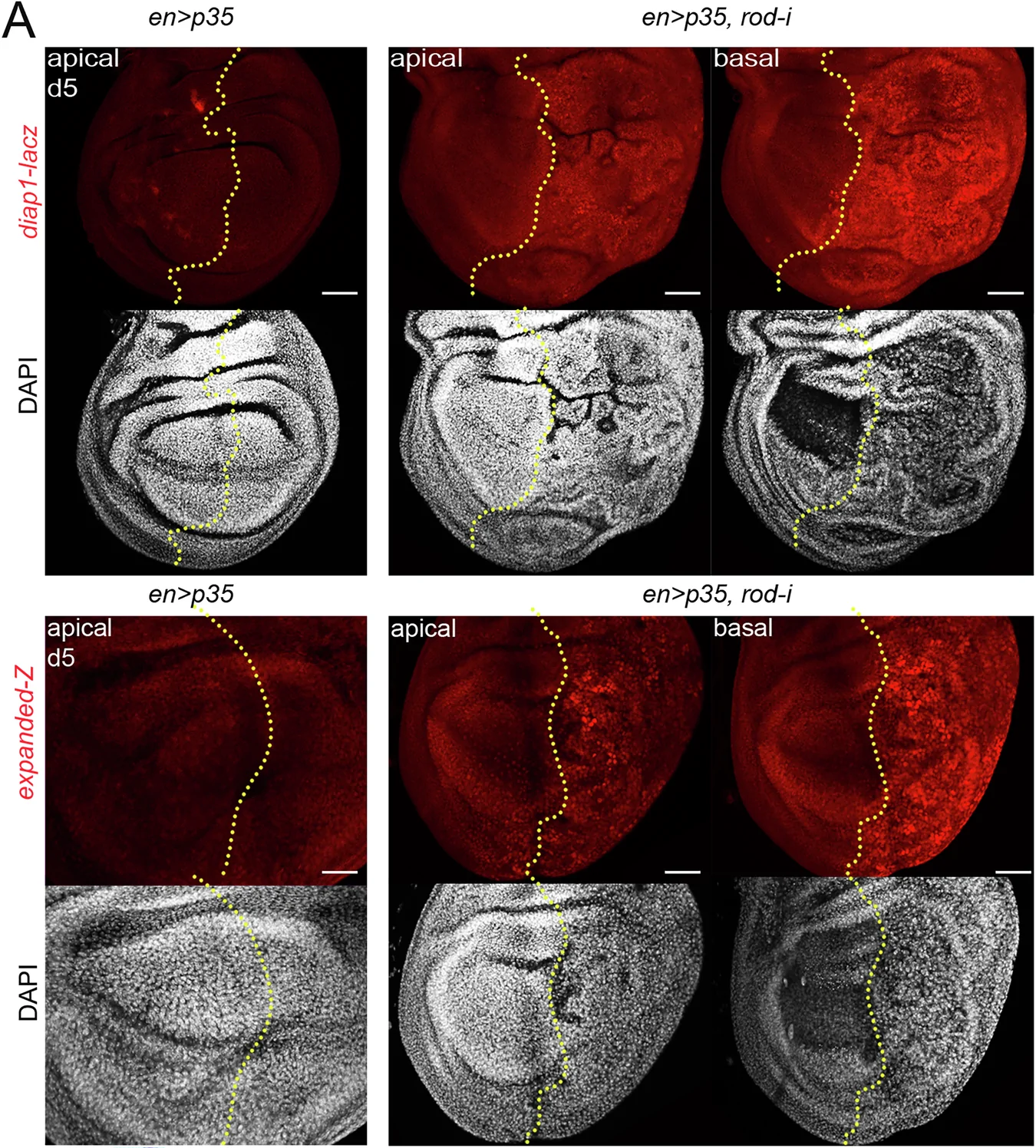
Yorkie targets are activated in aneuploidy-induced senescent cells (related to Fig. 3). (**A**) Control wing discs (first column) and wing discs subjected to CIN (remaining columns), stained to visualize *DIAP1-lacZ* (red, **A**), *expanded-lacZ* (red, **A**), and DAPI (white in **A**), and expressing the indicated transgenes in the P compartment under the control of the *en-gal4* driver. Scale bars, 50 µm. Discs were dissected 5 days AEL.

### A negative effect of CIN tumours on growth and survival of neighbouring cells

**Figure EV4:**
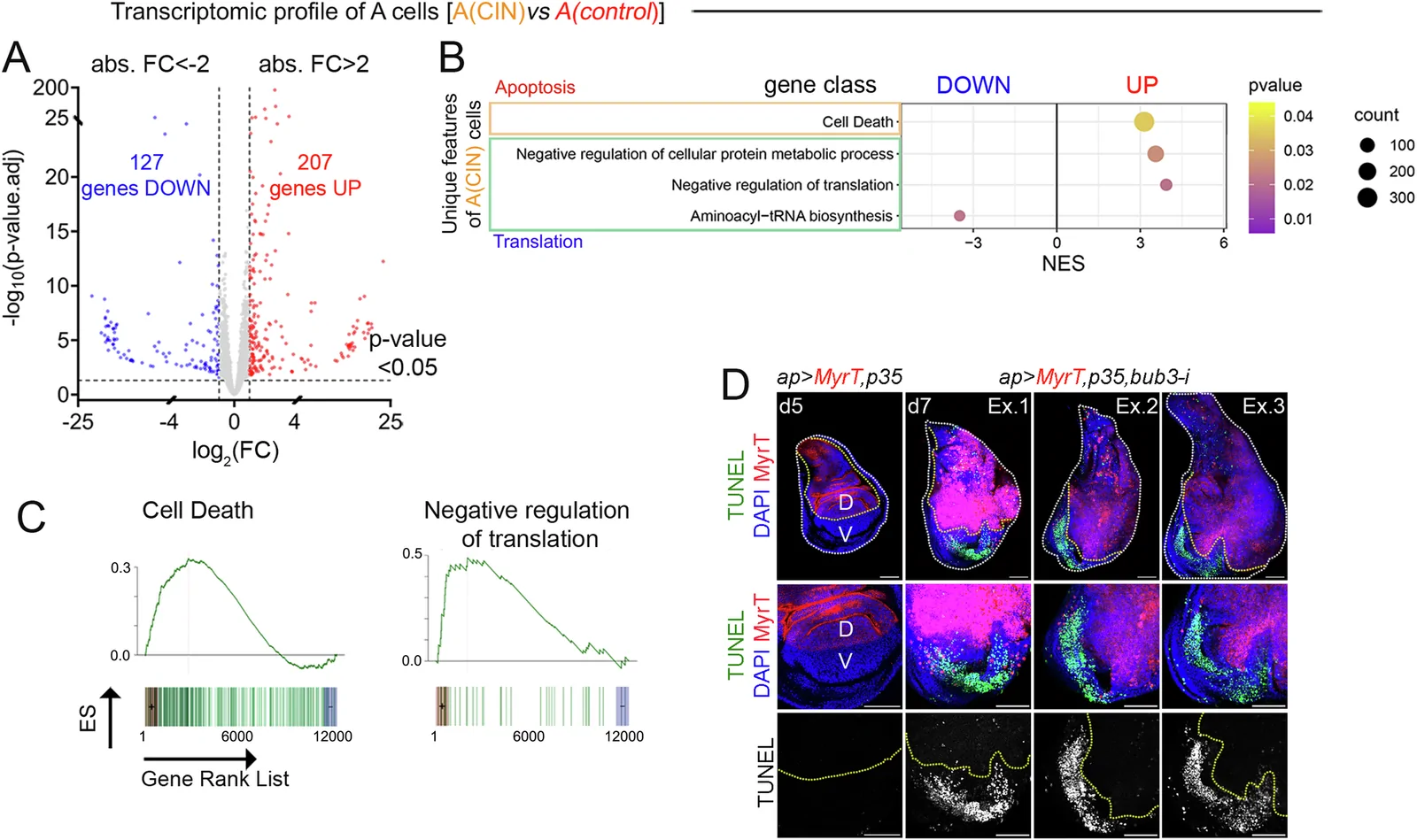
A non-autonomous effect of CIN tumours on the transcriptomic landscape of the neighbouring tissue (related to Fig. 3). (**A**) Volcano plot of all genes that are non-autonomously up- (red dots, FC > 2 and *p*-value < 0.05) or down-regulated (blue dots, FC < −2 and *p*-value < 0.05), or not differentially expressed (grey dots, *p*-value > 0.05 or 2 > FC > −2) in the A compartment of wing discs bearing CIN tumours in the P compartment when compared to A cells of control discs. Fold changes and *p*-values were calculated using the DESeq2 R package. *p*-values were adjusted for multiple comparisons with the Benjamini-Hochberg algorithm. (**B**) Bubble plot representing Gene Ontology (GO) enrichment analyses of the A compartment of wing discs bearing CIN tumours in the P compartment when compared to P cells of control discs. Statistics from the roastgsa enrichment algorithm of selected pathways are shown, where X axis corresponds to the normalized enrichment score (NES), size of the bubble represents the number of measured genes and colour-scale represents the associated *p*-value. See the methods section for details on methodology. In (**A**, **B**), *n* (number of technical replicates per genotype) = 4. (**C**) Mountain plots representing Enrichment Score (ES) upon Gene Set Enrichment Analysis (GSEA) of the A compartment of wing discs bearing CIN tumours in the P compartment when compared to A cells of control discs. GO classes: Cell death - GO0008219, Negative regulation of translation - GO0017148. (**D**) Control wing disc (first column) and three examples of wing discs subjected to CIN (remaining columns), and expressing the indicated transgenes in the dorsal (D) compartment under the control of the *ap-gal4* driver, stained for MyrT (red), TUNEL (green or white) and DAPI (blue). Scale bars, 50 µm. Days of dissection after egg laying are as indicated.

**Figure 5:**
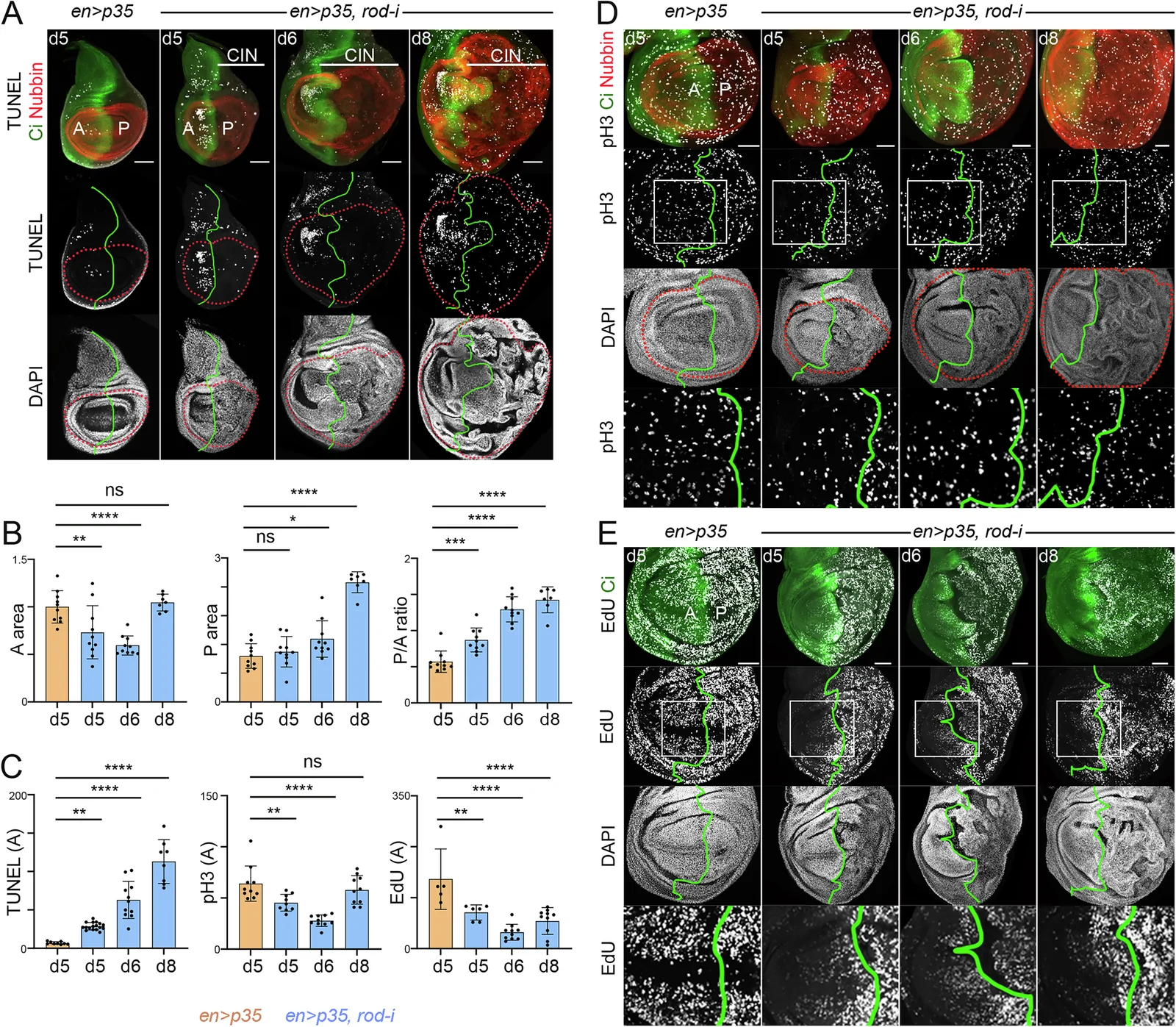
A non-autonomous effect of CIN tumours on the proliferation, growth, and survival of the neighbouring tissue. (**A**) Control wing discs (first column) and wing discs subjected to CIN (remaining columns), and expressing p35 in the P compartment under the control of the *en-gal4* driver, stained for Ci (green, to label the A compartment), DAPI (white), Nubbin (red, to label the wing pouch cells), and TUNEL (white). The tissue subjected to CIN is marked. Note the non-autonomous induction of cell death in A cells. (**B**, **C**) Histograms plotting in (**B**) the normalized size of the A and P compartments and the size ratio between these compartments, and in (**C**) the amount of TUNEL, pH3, and EdU incorporation in the A wing pouch compartment (normalized to the size of the corresponding compartment) of wing discs expressing the indicated transgenes in the P compartment under the control of the *en-gal4* driver. Graphs represent mean. *p < 0.05, **p < 0.01, ***p < 0.001, ****p < 0.0001, ns, not significant. One-way ANOVA with Dunnett’s multiple comparison test, *n* = number of biological replicates. Sample size and *p* value: (**B**, A area): *en* > *p35* d5 (*n* = 10) vs *en* > *p35, rod-i* d5 (*n* = 10) (*p* = 6.85 × 10^−3^); *en* > *p35* d5 (*n* = 10) vs *en* > *p35, rod-i* d6 (*n* = 10) (*p* = 5.77 × 10^−5^); and *en* > *p35, rod-i* d5 (*n* = 10) vs *en* > *p35, rod-i* d8 (*n* = 7) (*p* = 0.92); (**B**, P area): *en* > *p35* d5 (*n* = 10) vs *en* > *p35, rod-i* d5 (*n* = 10) (*p* = 0.86); *en* > *p35* d5 (*n* = 10) vs *en* > *p35, rod-i* d6 (*n* = 10) (*p* = 0.04); and *en* > *p35, rod-i* d5 (*n* = 10) vs *en* > *p35, rod-i* d8 (*n* = 7) (*p* = 2.91 × 10^−11^); (**B**, P/A ratio): *en* > *p35* d5 (*n* = 10) vs *en* > *p35, rod-i* d5 (*n* = 10) (*p* = 8.86 × 10^−4^); *en* > *p35* d5 (*n* = 10) vs *en* > *p35, rod-i* d6 (*n* = 10) (*p* = 8.80 × 10^−11^); and *en* > *p35, rod-i* d5 (*n* = 10) vs *en* > *p35, rod-i* d8 (*n* = 7) (*p* = 1.56 × 10^−11^); (**C**, TUNEL (A)): *en* > *p35* d5 (*n* = 9) vs *en* > *p35, rod-i* d5 (*n* = 17) (*p* = 8.90 × 10^−3^); *en* > *p35* d5 (*n* = 9) vs *en* > *p35, rod-i* d6 (*n* = 11) (*p* = 1.69 × 10^−8^); and *en* > *p35, rod-i* d5 (*n* = 9) vs *en* > *p35, rod-i* d8 (*n* = 8) (*p* = 2.22 × 10^−16^); (**C**, pH3 (A)): *en* > *p35* d5 (*n* = 10) vs *en* > *p35, rod-i* d5 (*n* = 9) (*p* = 6.04 × 10^−3^); *en* > *p35* d5 (*n* = 10) vs *en* > *p35, rod-i* d6 (*n* = 10) (*p* = 3.61 × 10^−7^); and *en* > *p35, rod-i* d5 (*n* = 10) vs *en* > *p35, rod-i* d8 (*n* = 10) (*p* = 0.54); (**C**, EdU (A)): *en* > *p35* d5 (*n* = 5) vs *en* > *p35, rod-i* d5 (*n* = 6) (*p* = 2.88 × 10^−3^); *en* > *p35* d5 (*n* = 5) vs *en* > *p35, rod-i* d6 (*n* = 10) (*p* = 1.85 × 10^−6^); and *en* > *p35, rod-i* d5 (*n* = 5) vs *en* > *p35, rod-i* d8 (*n* = 10) (*p* = 6.84 × 10^−5^). (**D**, **E**) Control wing discs (first column) and wing discs subjected to CIN (remaining columns), and expressing p35 in the P compartment under the control of the *en-gal4* driver, stained for Ci (green), DAPI (white), Nubbin (red), pH3 (white, **D**), and EdU (white, **E**). High magnification of the squared regions is shown in the lower panels. Note the non-autonomous block of cell proliferation in A cells. In (**A**, **D**, **E**), scale bars, 50 µm, days of dissection after egg laying are as indicated, and the boundary between A and P cells is labelled by a green line. See also Fig. EV4 and Dataset EV3. Source data are available online for this figure.

We noticed that the transcriptional profile of the neighbouring cell population (cells located in the nearby A compartment and not subject to CIN, Fig. 1B) showed upregulation of genes involved in cell death and a reduction in translation activity (Fig. EV4A–C). We next examined the response of this cell population (A compartment) to the nearby hyperplastic tissue (P compartment) subject to CIN and additional blockage of the apoptotic pathway at different time points of development. As anticipated, the P compartment showed a steady increase in size, whereas the size of the A compartment was significantly reduced compared with controls (Fig. 5A, quantified in Fig. 5B). After eight days of development, the A compartment reached the expected size of a control A compartment, while the P compartment continued to grow. The differential growth dynamics of CIN and neighbouring cell populations were also observed when analysing the ratio of their corresponding sizes [P/A ratio; Fig. 5B]. Interestingly, the negative impact of the CIN tissue on the growth dynamics of the A compartment was accompanied by an increase in the number of dying cells labelled by TUNEL staining (Fig. 5A, quantified in Fig. 5C), a reduction in mitotic activity labelled with pH3 antibody (Fig. 5D, quantified in Fig. 5C), and a decrease in EdU incorporation (Fig. 5E, quantified in Fig. 5C). Non-autonomous cell death was also observed upon induction of CIN in other regions of the wing primordium (Fig. EV4D).

**Figure 6:**
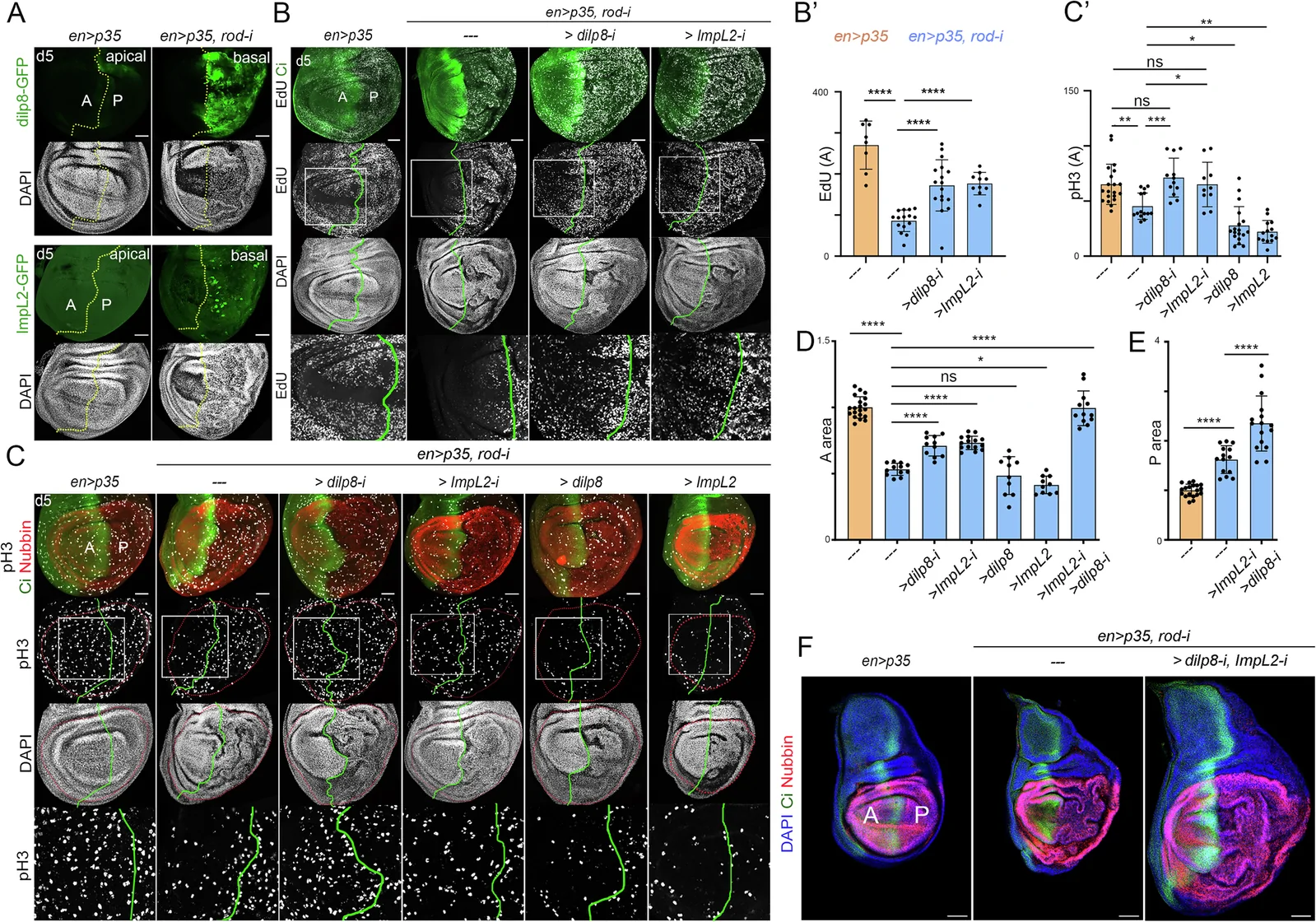
A growth suppressor role of Dilp8/Relaxin and ImpL2/IGFBP7 in CIN tumours. (**A**–**C**) Control wing discs (first column) and wing discs subjected to CIN (remaining columns), stained to visualize Dilp8-GFP (green, **A**), ImpL2-GFP (green, **A**), EdU (white, **B**), pH3 (white, **C**), DAPI (white), Nubbin (red, to label the wing pouch cells, **C**), and Ci (green, to label the A compartment, **B**, **C**), and expressing the indicated transgenes in the P compartment under the control of the *en-gal4* driver. High magnifications of the squared regions are shown in the lower panels in (**B**, **C**). The boundary between A and P cells is labelled by a green line. (**B’**, **C’**,** D**,** E**) Histograms plotting the amount of EdU incorporation and pH3 in the A wing pouch compartment (normalized to the size of the corresponding compartment, **B’**, **C’**), and the normalized size of the A (**D**) and P (**E**) wing pouch compartments of wing discs expressing the indicated transgenes in the P compartment under the control of the *en-gal4* driver. Graphs represent mean and standard deviation. *p < 0.05, **p < 0.01, ***p < 0.001, ****p < 0.0001, ns, not significant. One-way ANOVA with Šídák’s multiple comparisons test, *n* = number of biological replicates. Sample size and *p* value: (**B’**, EdU(A)): *en* > *p35* (*n* = 8) vs *en* > *p35, rod-i* (*n* = 15) (*p* = 4,28 × 10^−11^); *en* > *p35, rod-i* (*n* = 15) vs *en* > *p35, rod-i, dilp8-i* (*n* = 17) (*p* = 1.62 × 10^−5^); and *en* > *p35, rod-i* (*n* = 15) vs *en* > *p35, rod-i, ImpL2-i* (*n* = 10) (*p* = 7.28 × 10^−5^); (**C’**, pH3(A)): *en* > *p35* (*n* = 20) vs *en* > *p35, rod-i* (*n* = 14) (*p* = 5.50 × 10^−3^); *en* > *p35, rod-i* (*n* = 14) vs *en* > *p35, rod-i, dilp8-i* (*n* = 12) (*p* = 7.93 × 10^−4^); *en* > *p35, rod-i* (*n* = 14) vs *en* > *p35, rod-i, ImpL2-i* (*n* = 10) (*p* = 0.03); *en* > *p35, rod-i* (*n* = 14) vs *en* > *p35, rod-i, dilp8* (*n* = 18) (*p* = 0.02); *en* > *p35, rod-i* (*n* = 14) vs *en* > *p35, rod-i, ImpL2* (*n* = 14) (*p* = 2.02 × 10^−3^) ; *en* > *p35* (*n* = 20) vs *en* > *p35, rod-i, dilp8-i* (*n* = 12) (*p* = 0.92); *en* > *p35* (*n* = 20) vs *en* > *p35, rod-i, ImpL2-i* (*n* = 10) (*p* = 1)); (**D**, A area): *en* > *p35* (*n* = 20) vs *en* > *p35, rod-i* (*n* = 12) (*p* = 1.41 × 10^−23^); *en* > *p35, rod-i* (*n* = 12) vs *en* > *p35, rod-i, dilp8-i* (*n* = 11) (*p* = 3.82 × 10^−5^); vs *en* > *p35, rod-i, ImpL2-i* (*n* = 15) (*p* = 6.20 × 10^−7^); vs *en* > *p35, rod-i, dilp8* (*n* = 10) (*p* = 0.78); vs *en* > *p35, rod-i, ImpL2* (*n* = 10) (*p* = 1.63 × 10^−2^); and vs *en* > *p35, rod-i, ImpL2-i, dilp8-i* (*n* = 12) (*p* = 1.76 × 10^−20^); (**E**, P area): *en* > *p35* (*n* = 19) vs *en* > *p35, rod-i* (*n* = 14) (*p* = 2.32 × 10^−5^); and *en* > *p35, rod-i* (*n* = 14) vs *en* > *p35, rod-i, ImpL2-i, dilp8-i* (*n* = 16) (*p* = 2.86 × 10^−6^). (**F**) Control wing disc (first column) and wing discs subjected to CIN (remaining columns), stained to visualize DAPI (blue), Nubbin (red), and Ci (green), and expressing the indicated transgenes in the P compartment under the control of the *en-gal4* driver. In (**A**–**C**, **F**), scale bars, 50 µm, and discs were dissected 5 days after egg laying. See also Fig. EV5 and Dataset EV3. Source data are available online for this figure.

**Figure EV5:**
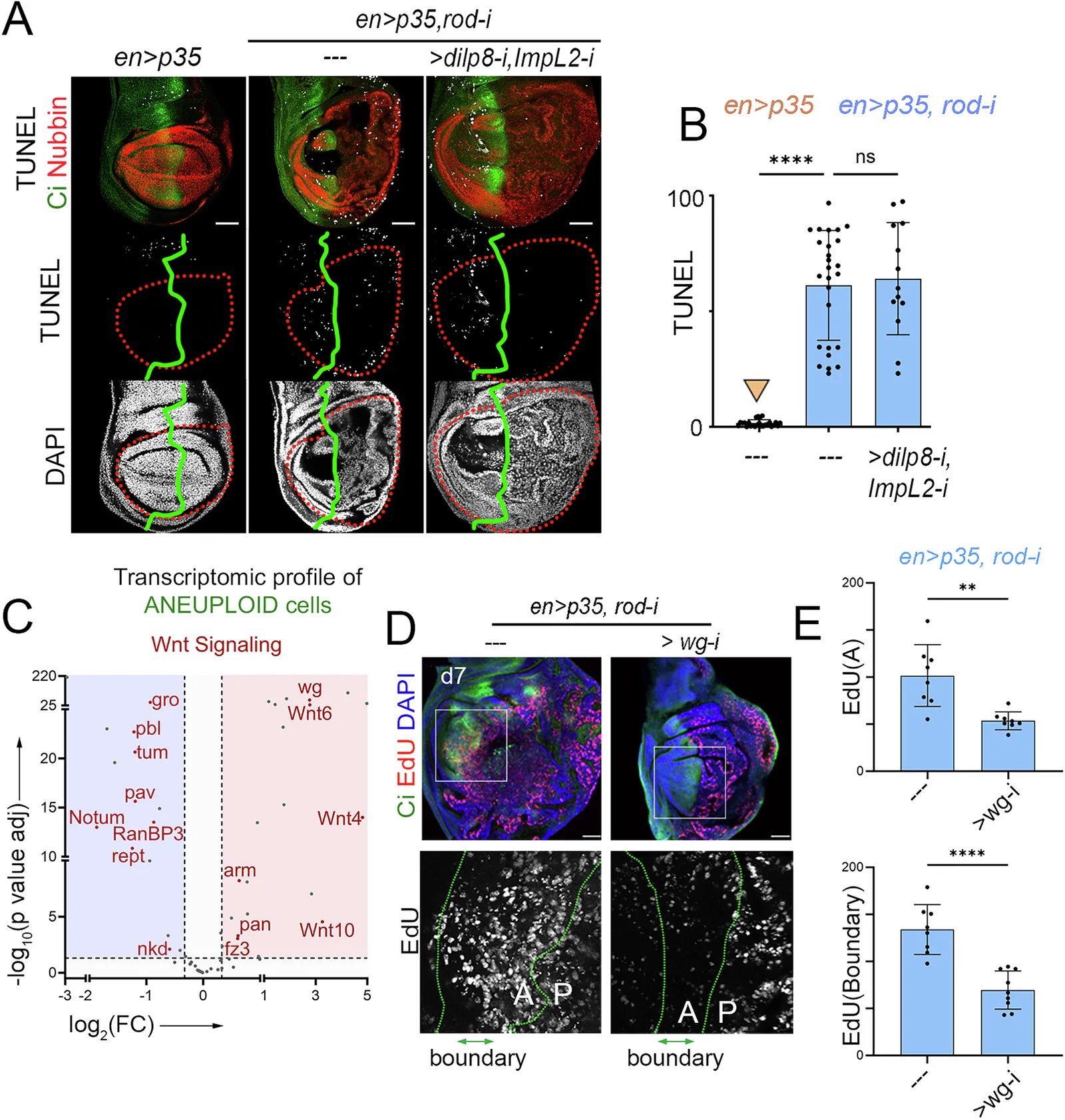
Contribution of Dilp8/Relaxin, ImpL2/IGFBP7 and Wingless to the non-autonomous effects of CIN tumours on cell death and proliferation (related to Fig. 6). (**A**) Wing discs subjected to CIN, stained to visualize TUNEL (white), DAPI (white), Nubbin (red, to label the wing pouch cells), Ci (green, to label the A compartment), and expressing the indicated transgenes in the P compartment under the control of the *en-gal4* driver. The boundary between A and P cells is labelled by a green line. (**B**, **E**) Histograms plotting the amount of TUNEL (**B**) or EdU signal (**E**) in the A wing pouch compartments or wing pouch AP boundary (normalized to the size of the corresponding territories) of wing discs expressing the indicated transgenes in the P compartment under the control of the *en-gal4* driver. Graphs represent mean and standard deviation. *p < 0.05, **p < 0.01, ***p < 0.001, ****p < 0.0001, ns, not significant. One-way ANOVA with Šídák’s multiple comparisons test (**B**) and Unpaired t test (**E**), *n* = number of biological replicates. Sample size and *p* value: (**B**, TUNEL (A)): *en* > *p35* (*n* = 26) vs *en* > *p35, rod-i* (*n* = 26) (*p* = 6.71 × 10^−17^); *en* > *p35, rod-i* (*n* = 26) *vs en* > *p35, rod-i, dilp8-i, ImpL2-i* (*n* = 13) (*p* = 0.87); (**E**, EdU (A)): *en* > *p35, rod-i* (*n* = 8) vs *en* > *p35, rod-i, wg-i* (*n* = 8) (*p* = 1.37 × 10^−3^); (**E**, EdU (Boundary)): *en* > *p35, rod-i* (*n* = 8) vs *en* > *p35, rod-i, wg-i* (*n* = 9) (*p* = 4.66 × 10^−5^). (**C**) Volcano plot representing selected genes in ‘Wnt Signalling - GO0060070 and GO0090090’ classes that are, differentially upregulated (red area, FC > 1.3 and *p*-value < 0.05), downregulated (blue area, FC < −1.3 and *p*-value < 0.05), or not differentially expressed (grey area, *p*-value > 0.05 or 1.3 > FC > −1.3) in aneuploid cells when compared to P cells of control discs. (**D**) Wing discs subjected to CIN, stained to visualize TUNEL EdU (red or white), DAPI (blue), Ci (green), and expressing the indicated transgenes in the P compartment under the control of the *en-gal4* driver. High magnifications of the squared regions are shown in the lower panels. Note that the EdU incorporation of the anterior stripe abutting the P compartment in CIN tissues, demarcated by two green lines and double-headed arrow, is not observed upon targeted depletion of *wingless* in the P compartment. In (**A**, **D**), scale bars, 50 µm and discs were dissected 5 days AEL. See also Dataset EV3.

We next addressed whether the observed response of the A compartment to the nearby CIN tissue was driven by any of the signalling molecules secreted by aneuploidy-induced senescent cells (Table 1). Interestingly, Dilp8, a molecule secreted by damaged tissues and well known to block the production of Ecdysone (Sanchez et al, 2019; Boulan et al, 2019), a systemic hormone driving the larval to pupal transition and promoting proliferative growth, was among the most upregulated secreted proteins in aneuploidy-induced senescent cells (Fig. 2A and Table 1). Using a GFP protein trap reporter, we confirmed the expression of Dilp8 in aneuploidy-induced senescent cells (Fig. 6A). Depletion of *dilp8* in the CIN tissue (with a *UAS-RNAi*) restored mitotic activity and EdU incorporation levels, but not the number of apoptotic cells, in the adjacent A compartment (Fig. 6B,C, quantified in Figs. 6B’,C’ and EV5A,B). Interestingly, Dilp8 overexpression reduced mitotic activity and the size of the A compartment even further (Fig. 6C, quantified in Fig. 6C’). Since experimental manipulation of Dilp8 levels in the P compartment was unable to have a significant effect on the size of the nearby A compartment (Fig. 6D), we searched for additional genes expressed in aneuploidy-induced senescent cells with a known role in inhibiting proliferative growth and found ImpL2, an orthologue of IGFBP7 that binds to and inhibits the function of *Drosophila* insulin-like peptides (Arquier et al, 2008; Honegger et al, 2008) and consequently the growth-promoting PI3K signalling pathway (Table 1), an upregulation that was confirmed with a GFP protein trap reporter (Fig. 6A). Depletion of *ImpL2* in the CIN tissue (with a *UAS-RNAi*) restored mitotic activity and EdU incorporation levels (Fig. 6B,C, quantified in Fig. 6B’,C’) in the adjacent A compartment but not the number of apoptotic cells (Fig. EV5A,B), and ImpL2 overexpression reduced mitotic activity even further (Fig. 6C, quantified in Fig. 6C’). As observed in the case of Dilp8, experimental manipulation of ImpL2 levels had no significant impact on the size of the A compartment either (Fig. 6D). Interestingly, co-depletion of both *dilp8* and *impL2* was able to dampen the reduction in the size of the A compartment caused by the nearby CIN tissue (Fig. 6D). However, we noticed that the size of the P compartment was also dramatically increased upon *dilp8* and *impL2* co-depletion (Fig. 6E), thus resulting in visibly bigger tumours (Fig. 6F). These results are consistent with the previously reported systemic effects of these two secreted factors (Honegger et al, 2008; Boulan et al, 2019; Sanchez et al, 2019), and point to an additive tumour-suppressive role of these two systemic molecules, most probably by interfering with Ecdysone and dILPs, two growth-promoting systemic hormones. The reduction in the proliferative activity of the A compartment was not observed in those cells abutting the CIN tissue (Figs. 5E and EV5D), thus pointing to a signal originating in the P compartment and counteracting the negative effects of Dilp8 and ImpL2 on proliferation. Indeed, the proliferative activity of these cells relies on the activity of the mitogenic molecule Wg produced by senescent cells (Table 1, (Gracia-Latorre et al, 2022), see also Fig. EV5C), as Wg depletion reduced the levels of EdU incorporation in these cells (Fig. EV5D,E).

### A feed-forward loop driving tumour growth

**Figure 7:**
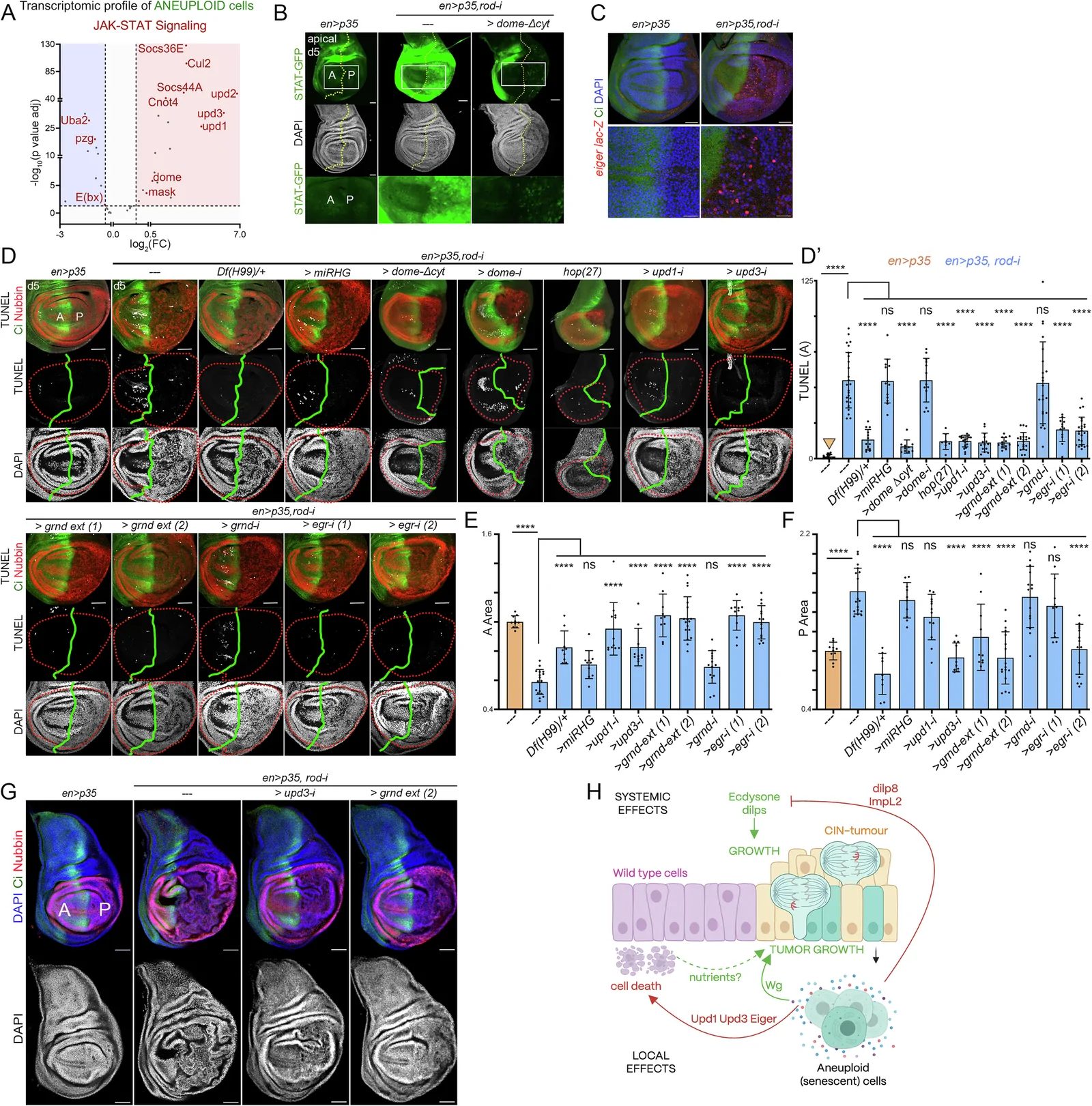
A feed-forward loop involved in the growth of CIN tumours. (**A**) Volcano plot representing selected genes in JAK-STAT Signalling - GO0097696 class that are differentially upregulated (red area, FC > 1.3 and *p*-value < 0.05), downregulated (blue area, FC < −1.3 and *p*-value < 0.05), or not differentially expressed (white area, *p*-value > 0.05 or 1.3 > FC > −1.3) in aneuploid cells when compared to P cells of control discs. Fold changes and *p*-values were calculated using the DESeq2 R package. *p*-values were adjusted for multiple comparisons with the Benjamini-Hochberg algorithm. *n* (number of technical replicates per genotype) = 4. (**B**,** C**, **D**,** G**) Control wing discs (first column) and wing discs subjected to CIN (remaining columns) stained to visualize STAT-GFP (green, **B**), *eiger-lacZ* (red, **C**), TUNEL (white, **D**), Nubbin (red, to label the wing pouch cells, **D**, **G**), and Ci (green, to label the A compartment, **C**,** D**, **G**), and DAPI (white in **B**, **D**, blue in **C**, **G**) and expressing the indicated transgenes in the P compartment under the control of the *en-gal4* driver. Scale bars, 50 µm. Discs were dissected 5 days AEL. The boundary between A and P cells is labelled by a green or a yellow dashed line, or by Ci staining. High magnification of the squared regions are shown in the lower panels in (**B**). (**D’**,** E**,** F**) Histograms plotting the amount of TUNEL (**D’**), and the size of the A (**E**) and P (**F**) wing pouch compartments of wing discs expressing the indicated transgenes in the P compartment under the control of the *en-gal4* driver. Graphs represent mean and standard deviation *p < 0.05, **p < 0.01, ***p < 0.001, **** *p* < 0.0001, ns, not significant. One-way ANOVA with Šídák’s multiple comparisons test, *n* = number of biological replicates. Sample size and *p* value: (**D’**, TUNEL(A)): *en* > *p35* (*n* = 19) vs *en* > *p35, rod-i* (*n* = 25) (*p* = 2.00 × 10^−28^); *en* > *p35, rod-i* (*n* = 25) *vs en* > *p35, rod-Ii; Df(H99)/+* (*n* = 13) (*p* = 5.02 × 10^−16^); *en* > *p35, rod-i* (*n* = 25) *vs en* > *p35, rod-i, miRHG* (*n* = 13) (*p* = *1*); *en* > *p35, rod-i* (*n* = 25) *vs en* > *p35, rod-i, dome-Δcyt* (*n* = 10) (*p* = 1.79 × 10^−16^); *en* > *p35, rod-i* (*n* = 25) *vs en* > *p35, rod-i, dome-i* (*n* = 13) (*p* = *1*); *en* > *p35, rod-i* (*n* = 25) *vs hop(27)/Y; en* > *p35, rod-i*, (*n* = 8) (*p* = 1.43 × 10^−12^); *en* > *p35, rod-i* (*n* = 25) *vs en* > *p35, rod-i, upd1-i* (*n* = 20) (*p* = 1.58 × 10^−20^); *en* > *p35, rod-i* (*n* = 25) *vs en* > *p35, rod-i, upd3-i* (*n* = 14) (*p* = 6.34 × 10^−18^); *en* > *p35, rod-i* (*n* = 25) *vs en* > *p35, rod-i, grnd-ext (1)* (*n* = 13) (*p* = 2.66 × 10^−17^); *en* > *p35, rod-i* (*n* = 25) *vs en* > *p35, rod-i, grnd-ext (2)* (*n* = 21) (*p* = 5.50 × 10^−21^); *en* > *p35, rod-i* (*n* = 25) *vs en* > *p35, rod-i, grnd-i* (*n* = 22) (*p* = 1)*; en* > *p35, rod-i* (*n* = 25) *vs en* > *p35, rod-i, egr-i (1)* (*n* = 12) (*p* = 3.86 × 10^−11^); and *en* > *p35, rod-i* (*n* = 25) *vs en* > *p35, rod-i, egr-i (2)* (*n* = 23) (*p* = 3.40 × 10^−16^); (**E**, A area): *en* > *p35* (*n* = 10) vs *en* > *p35, rod-i* (*n* = 17) (*p* = 3.96 × 10^−13^); *en* > *p35, rod-i* (*n* = 17) *vs en* > *p35, rod-i; Df(H99)/+* (*n* = 9) (*p* = 5.27 × 10^−5^); *vs en* > *p35, rod-i, miRHG* (*n* = 10) (*p* = 1.393393e^−1^); *vs en* > *p35, rod-i, upd1-i* (*n* = 12) (*p* = 8.187640e^−12^); *en* > *p35, rod-i* (*n* = 17) *vs en* > *p35, rod-i, upd3-i* (*n* = 10) (*p* = 2.38 × 10^−5^); *en* > *p35, rod-i* (*n* = 17) *vs en* > *p35, rod-i, UAS grnd-ext (1)* (*n* = 10) (*p* = 2.72 × 10^−15^); *en* > *p35, rod-i* (*n* = 17) *vs en* > *p35, rod-i, grnd-ext (2)* (*n* = 17) (*p* = 8.64 × 10^−18^); *en* > *p35, rod-i* (*n* = 17) *vs en* > *p35, rod-i, grnd-i* (*n* = 14) (*p* = 0.18); *en* > *p35, rod-i* (*n* = 17) *vs en* > *p35, rod-i, egr-i (1)* (*n* = 11) (*p* = 5.13 × 10^−16^); *and en* > *p35, rod-i* (*n* = 17) *vs en* > *p35, rod-i, egr-i (2)* (*n* = 14) (*p* = 4.30 × 10^−15^); (**F**, P area): *en* > *p35* (*n* = 10) vs *en* > *p35, rod-i* (*n* = 17) (*p* = 1.11 × 10^−7^); *en* > *p35, rod-i* (*n* = 17) *vs en* > *p35, rod-i; Df(H99)/+* (*n* = 9) (*p* = 2.95 × 10^−12^); *en* > *p35, rod-i* (*n* = 17) *vs en* > *p35, rod-i, miRHG* (*n* = 10) (*p* = 0.99); *en* > *p35, rod-i* (*n* = 17) *vs en* > *p35, rod-i, upd1-i* (*n* = 12) (*p* = 5.846245 × 10^−2^); *en* > *p35, rod-i* (*n* = 17) *vs en* > *p35, rod-i, upd3-i* (*n* = 10) (*p* = 4.49 × 10^−9^); *en* > *p35, rod-i* (*n* = 17) *vs en* > *p35, rod-i, grnd-ext (1)* (*n* = 10) (*p* = 6.51 × 10^−5^); *en* > *p35, rod-i* (*n* = 17) *vs en* > *p35, rod-i, grnd-ext (2)* (*n* = 17) (*p* = 1.10 × 10^−11^); *en* > *p35, rod-i* (*n* = 17) *vs en* > *p35, rod-i, grnd-i* (*n* = 14) (*p* = 0.99); *en* > *p35, rod-i* (*n* = 17) *vs en* > *p35, rod-i, egr-i (1)* (*n* = 11) (*p* = 0.73); and *en* > *p35, rod-i* (*n* = 17) *vs en* > *p35, rod-i, egr-i (2)* (*n* = 14) (*p* = 1.30 × 10^−8^). (**H**) Model: in epithelial tissues subjected to restricted induction of CIN in the P cell population (yellow), aneuploid cells (green) delaminate basally and enter a senescent state. Members of the SASP impact on the neighbouring wild type cell population (the A cell population depicted in purple) locally of systemically to induce cell death and regulate cell proliferation and growth. Non-autonomous cell death feeds back to the tumour to enhance its growth. Model created in BioRender. Barrio, L. (2026) https://BioRender.com/bjr8gmi. See also Dataset EV1 and EV3. Source data are available online for this figure.

The non-autonomous induction of cell death and reduction in tissue size caused by CIN tumours was, at least in part, a consequence of activation of the apoptotic pathway as halving the doses of the pro-apoptotic genes *reaper*, *hid* and *grim* (in *Df(H99)/+* larvae) caused a reduction in the levels of non-autonomous cell death (Fig. 7D,D’) and restored the size of the nearby A compartment (Fig. 7E). We noticed that targeted depletion of these three genes with the use of a synthetic micro-RNA [*UAS-miRHG*, (Siegrist et al, 2010)] only in the P compartment did not have any effect on the levels of cell death and on the size of the A compartment (Fig. 7D,D’,E). In search of secreted molecules involved in the non-autonomous induction of the apoptotic pathway (Table 1), we noticed that aneuploidy-induced senescent cells showed upregulation, on the one hand, of elements of the JAK/STAT pathway, including ligands (cytokines Upd1, Upd2, and Upd3, Table 1) and the receptor Domeless (Dome, Fig. 7A), and, on the other hand, the TNF-α ligand Eiger (Fig. 2A and Table 1), which activates the JNK signalling pathway through its receptor Grindelwald (Grnd, (Andersen et al, 2015)). Both JNK and JAK/STAT signalling pathways are known in CIN tissues to induce apoptosis through transcriptional induction of pro-apoptotic genes (Barrio et al, 2023). We confirmed activation of the JAK/STAT pathway with a GFP transcriptional reporter (STAT-GFP (Bach et al, 2007)), and, most interestingly, we observed that it was also activated in the nearby A compartment (Fig. 7B). We confirmed induction of *eiger* expression with a transcriptional reporter (Fig. 7C, see also (Muzzopappa et al, 2017)). We next addressed the potential contribution of Upd ligands, the JAK/STAT pathway and Eiger to the effects of CIN tissues on the nearby wild-type cell population.

**Figure EV6:**
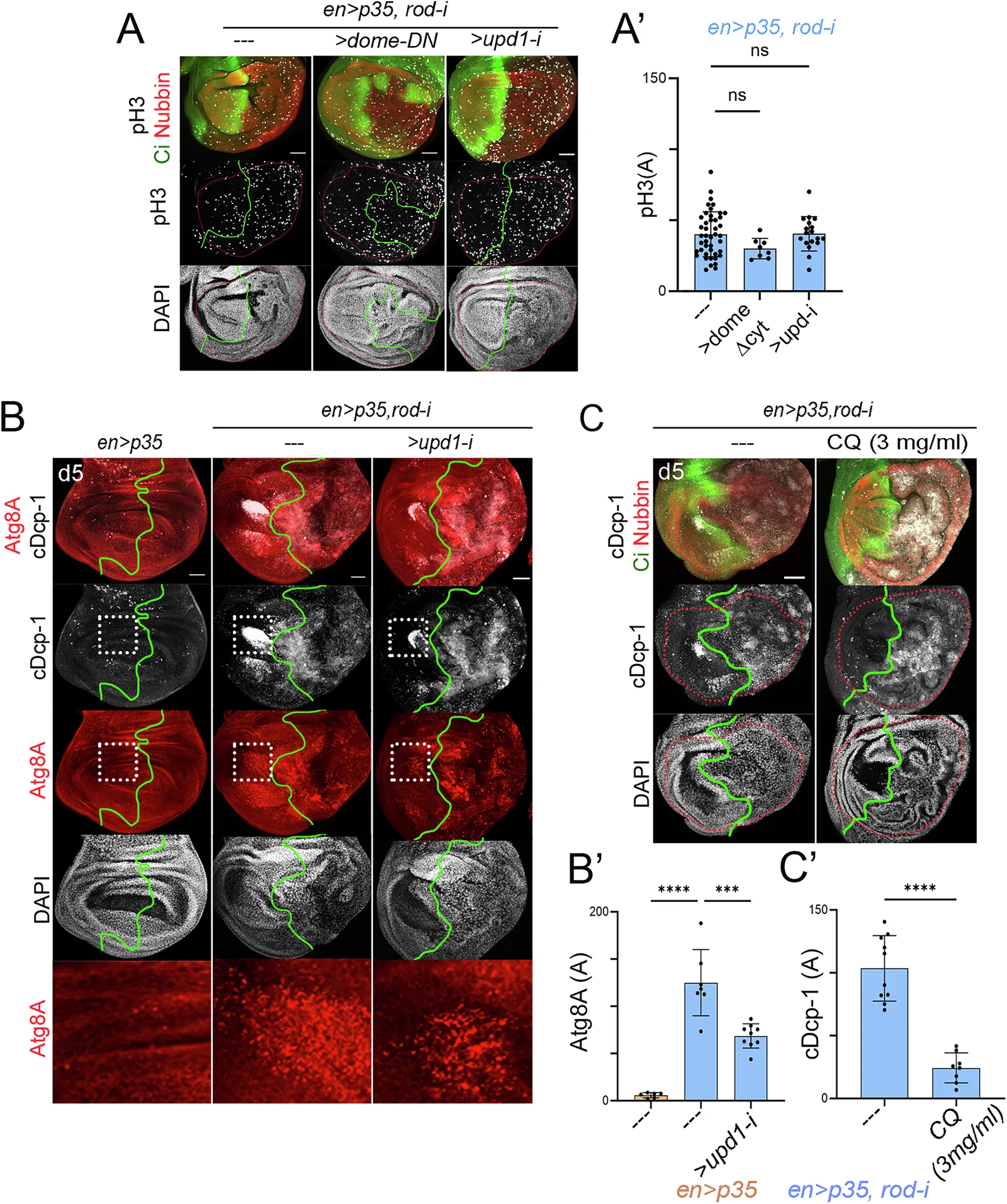
Contribution of Upd cytokines to the non-autonomous effects of CIN tumours on cell proliferation (related to Fig. 7). (**A**–**C**) Control wing discs (first column in **A**–**C**) and wing discs subjected to CIN (remaining columns) stained for pH3 (white, **A**), cDcp-1 (red and white, **B**; white, **C**), mCherry-Atg8 (red, **B**), –Nubbin (red, to label the wing pouch cells, **A**, **C**), and Ci (green, to label the A compartment, **A**, **C**), and expressing the indicated transgenes in the P compartment under the control of the *en-gal4* driver. Larvae in **C** were subjected to chloroquine (CQ) feeding for 72 h. High magnification of the squared regions in (**B**) are shown in the lower panels. Scale bars, 50 µm. Discs were dissected 5 days AEL. The boundary between A and P cells is labelled by a green line. (**A’**–**C’**) Histograms plotting the amount of pH3 signal (**A’**), mCherry-Atg8 signal (**B’**) or cDcp-1 signal (**C’**) in the A wing pouch compartment (normalized to the size of the corresponding compartment) of wing discs expressing the indicated transgenes in the P compartment under the control of the *en-gal4* driver. Graphs represent mean and standard deviation. *p < 0.05, **p < 0.01, ***p < 0.001, ****p < 0.0001, ns, not significant. One-way ANOVA with Šídák’s multiple comparisons test (**A’**, **B’**), Unpaired t-test (**C’**), *n*= number of biological replicates. Sample size and *p* value: (**A’**, pH3 (A)): *en* > *p35, rod-i* (*n* = 44) vs *en* > *p35, rod-i, dome-Δcyt* (*n* = 8) (*p* = 0.181); and *en* > *p35, rod-i* (*n* = 44) *vs en* > *p35, rod-i, upd1-i* (*n* = 18) (*p* = 0.99); (**B’**, Atg8 (A)): *en* > *p35* (*n* = 6) vs *en* > *p35, rod-i* (*n* = 7) (*p* = 1.07 × 10^−8^); and *en* > *p35, rod-i* (*n* = 7) *vs en* > *p35, rod-i, upd1-i* (*n* = 9) (*p* = 9.70 × 10^−5^); (**C’**, cDcp1 (A)): *en* > *p35, rod-i* (*n* = 10) *vs CQ treated en* > *p35, rod-i* (*n* = 8) (*p* = 6.24 × 10^−7^). See also Dataset EV3.

Expression of a truncated form of receptor Domeless lacking the intracellular domain in the CIN tissue (Dome-ΔCYT) led to a marked reduction in the levels of STAT-GFP signalling, not only cell autonomously but also in the nearby A compartment (Fig. 7B). This finding is consistent with the proposed role of Dome-ΔCYT in blocking JAK/STAT signalling by trapping the Upd ligands and competing with the endogenous receptor (Romão et al, 2021). Interestingly, the non-autonomous reduction in STAT-GFP levels caused by Dome-ΔCYT was accompanied by a decrease in the number of apoptotic cells (Fig. 7D, quantified in Fig. 7D’). In contrast, depletion of Dome by RNAi, which is expected to block ligand-dependent JAK/STAT signalling in a cell autonomous manner without trapping the Upd ligands, did not affect the neighbouring A compartment (Fig. 7D, quantified in Fig. 7D’). We found that both transgenes (*UAS-dome-ΔCYT* and *UAS-dome-RNAi*) caused a similar reduction in the size of the P compartment, most probably caused by the developmental role of the JAK/STAT pathway in regulating the growth of this compartment (Recasens-Alvarez et al, 2017). The non-autonomous reduction in the number of apoptotic cells caused by Dome-ΔCYT was also observed in tissues mutant for *hop* (the *Drosophila* JAK orthologue) and upon depletion of the cytokines *upd1 or upd3* by RNAi in CIN and aneuploid cells (Fig. 7D, quantified in Fig. 7D’). We observed that neither *upd1* depletion nor Dome-ΔCYT expression in CIN tissue ameliorated the non-autonomous reduction in proliferation dynamics (Fig. EV6A, quantified in Fig. EV6A’).

In a similar manner, depletion of *eiger* or expression of a dominant negative version of its receptor Grnd (Grnd-extra) in CIN tissues, known to trap Eiger ligand thus blocking its function (Andersen et al, 2015), reduced the number of apoptotic cells in the nearby A compartment (Fig. 7D, quantified in Fig. 7D’). Depletion of *grnd* by RNAi, which is expected to block Eiger-dependent JNK activation in a cell autonomous manner without trapping the Eiger ligand, did not affect the apoptotic levels in the neighbouring A compartment (Fig. 7D, quantified in Fig. 7D’). Most interestingly, depletion of Upd1, Upd3 or Eiger rescued the reduction in the size of the A compartment (Fig. 7E). Similar results were observed with Grnd-extra (Fig. 7E). All these results point to an additive role of multiple molecules secreted from aneuploidy-induced senescent cells and acting in nearby wild-type tissues to induce cell death and compromise growth.

We next analysed whether the non-autonomous cell death had any impact on the growth of the tumour itself. Interestingly, the reduction in the levels of non-autonomous cell death and the restoration of the size of the A compartment caused by depletion of Eiger, the Upd1 and Upd3 cytokines or expression of their truncated receptors was in all cases accompanied by a reduction in the size of the hyperplastic tissue (P compartment) subject to CIN and additional blockage of the apoptotic pathway (Fig. 7F,G). We noticed that depletion of *grindelwald* by RNAi did not have any impact on the size of the P compartment, thus ruling out an autocrine role of Eiger in CIN tumours [Fig. 7F, see also (Muzzopappa et al, 2017)]. Depletion of several genes of the JAK/STAT signalling pathway by RNAi was previously shown to have no impact on the size of CIN tumours either, thus ruling out also an autocrine role of Upd cytokines (Barrio et al, 2023). These results indicate that several SASP members act in an additive manner on neighbouring tissues to reduce growth through cell death and that these non-autonomous effects feed back to the tumour to reinforce its growth.

Although the classical apoptotic pathway appears to play a major role in the non-autonomous induction of cell death and reduction in tissue size exerted by Upd cytokines and Eiger, as cell death and tissue size were both largely rescued by halving the doses of the pro-apoptotic genes (Fig. 7D,D’,E), non-apoptotic cell death might also contribute to the observed effects on tissue size. On the one hand, Eiger and the JNK pathway have been reported to induce also non-apoptotic cell death through the production of ROS (Kanda et al, 2011). On the other hand, Upd cytokines and the JAK/STAT pathway, well known to induce autophagy in CIN tissues (Joy et al, 2021), might also cause cell death through autophagy. Consistent with this proposal, we noticed that in those regions within the nearby A compartment with the highest levels of apoptotic activity (monitored with an antibody to detect the cleaved version of the effector caspase Dcp1, cDcp1), autophagy was also induced, as reflected by the accumulation in puncta of a mCherry-tagged Atg8a under the control of the endogenous Atg8a promoter (Atg8a; Fig. EV6B). This induction was largely dependent on Upd1 derived from CIN tissue, as *upd1* depletion led to a significant reduction in the amount of Atg8a signal in the A compartment (Fig. EV6B,B’). Compromising autophagic flux, by supplementing the larval food with chloroquine, led to a significant reduction in the extent of cell death observed in the A compartment (Fig. EV6C,C’). We noticed that caspase activation in CIN cells, which play a non-apoptotic role by inducing cell motility in aneuploid cells, was not reduced with this treatment, which is consistent with their reported activation through transcriptional induction of pro-apoptotic genes by JNK and JAK/STAT signalling pathways (Barrio et al, 2023). Overall, these observations point to the potential contribution of different forms of cell death in the observed feed-forward loop involved in the growth of CIN tumours.

## Discussion

### The transcriptome of aneuploidy-induced senescence

From flies to mammals, CIN is widely recognized as a driver of cellular senescence, primarily through the generation of highly aneuploid karyotypes (Macedo et al, 2018; Barroso-Vilares et al, 2020; Santaguida et al, 2017; Joy et al, 2021), and cellular senescence and its associated senescence-associated secretory phenotype (SASP) are major triggers of inflammation and tumorigenesis (Gorgoulis et al, 2019). Here we have characterized the transcriptional profile of aneuploidy-induced senescence in an epithelial model of CIN in *Drosophila* that recapitulates most emerging cellular behaviours observed in mammalian epithelial tissues upon CIN (Rowald et al, 2016; Santaguida et al, 2017; Levine et al, 2017; Bakhoum et al, 2018; Benhra et al, 2018; Clemente-Ruiz et al, 2014, 2016; Dekanty et al, 2012; Morais da Silva et al, 2013; Muzzopappa et al, 2017). Thus, in fly epithelial tissues subjected to CIN and upon additional blockade of the apoptotic pathway, aneuploid cells are extruded from the epithelium and enter a state of senescence characterized by cell cycle arrest in G2, increased motility, and a highly secretory phenotype that drives the hyperplastic growth of the epithelium in a non-autonomous manner (Fusari et al, 2025). Consequently, CIN tissues acquire a tumour-like, unlimited growth potential and highly invasive behaviour. All these processes rely on the activity of the JNK signalling pathway. As it occurs from yeast to mammals, aneuploidy activates autophagy as the primary quality control mechanism for maintaining proteostasis in response to gene dosage imbalance and proteotoxic stress (Stingele et al, 2013; Santaguida et al, 2015). Saturated use of the autophagy machinery in these cells leads to the accumulation of dysfunctional mitochondria as a result of compromised mitophagy, and these mitochondria become active sources of ROS (Joy et al, 2021), which ultimately activate JNK via Ask1 (Muzzopappa et al, 2017). The use of the GAL4/UAS system to trigger CIN in only one half of the growing wing epithelium and a GFP transcriptional reporter of JNK (MMP1-GFP, (Uhlirova and Bohmann, 2006)) to track aneuploid cells has allowed us to characterize the transcriptional profile of aneuploidy-induced senescent cells, the epithelial layer subjected to CIN that enters a hyperplastic mode of growth, and the nearby wild-type population. Remarkably, most cellular responses to aneuploidy and senescence are robustly reflected at the transcriptional level, as shown by the up- or down-regulation of a large number of genes involved in any of these responses (Figs. 1 and 2). Thus, cell cycle progression is locked in aneuploid cells due to the downregulation of most genes involved in DNA replication (including all subunits of the DNA polymerase, and Orc and Mcm complexes) and G2/M transition (including *Dcdc25*/*stg*, *cdk1*, *cycA*, and *cycB*). The induction of autophagy, a process that delivers its cargo to lysosomes for degradation, is accompanied by the upregulation of many autophagy (*atg4a*, *atg8a*, *atg9*, *atg17*, and *atg18b*) and lysosomal genes (e.g., *Lamp1*, *Vps13D*, *Vps16A, Vamp7*, and *lqf*). Similarly, the reported ability of aneuploid cells to become motile through modulation of the actin cytoskeleton is accompanied by the upregulation of many actin cytoskeleton proteins, including Sqh, Cortactin, Rok, Fak, and Arpc2-5. One of the most remarkable properties of senescent cells is the SASP (Wang et al, 2024). Our transcriptomic analysis has unravelled a significant upregulation of genes involved in secretion (e.g., *Tango 5, 11, Sec 5, 6, 33, 24CD*, and *Rab 1, 2, 6, 7, 10*, and *35*), reinforced with the use of cellular reporters to unveil an enlarged secretory apparatus, and has allowed us to identify all the elements of the SASP, where 10% of the most upregulated genes in these cells encode for secreted proteins (Dataset EV2). Remarkably, many of the cellular behaviours observed in our fly model of CIN have been observed in recent years in mammalian tissues, thus reinforcing its translational impact. Thus, mammalian cells with complex karyotypes caused by chromosome segregation defects are also extruded from the epithelium (Rowald et al, 2016), and they are cell cycle-arrested, exhibit features of senescence, and produce pro-inflammatory signals (Santaguida et al, 2017). Most interestingly, CIN also drives tumorigenesis in mammals [e.g. (Levine et al, 2017)] and promotes metastasis (Bakhoum et al, 2018).

### An anti-apoptotic role of Yorkie in senescent cells

*Drosophila* CIN tissues are exposed to the activation of pro-apoptotic pathways JNK and JAK/STAT that lead to the elimination of aneuploid cells in the tissue (Dekanty et al, 2012; Barrio et al, 2023). It is the blockage of the apoptotic pathway by the expression of the baculovirus protein p35 what unravels a subversive role of JNK in driving cellular senescence, and JAK/STAT in promoting cell motility. However, the apoptotic pathway is not completely blocked under these conditions as shown by the high activity levels of effector caspases in the tissue, which have been shown to contribute in a non-apoptotic manner to the invasive behaviour of aneuploid cells (Barrio et al, 2023). These results point to the presence of an endogenous pathway that counteracts the pro-apoptotic activities of JNK and JAK/STAT. Similar to how senescent cells in mammals resist normal apoptotic pathways through expression of the Bcl2 anti-apoptotic protein (Martin et al, 2023), our results indicate that the Hippo/Yorkie pathway plays an analogous role in senescent fly cells. Activation of this pathway is most probably the result of JNK activation and cellular tension positively regulating the cortical localization of Ajuba expression to sequester Warts, thus allowing Yorkie to translocate into the nucleus and drive gene expression (Sun and Irvine, 2013; Rauskolb et al, 2014). Although DIAP1 (a negative regulator of the initiator Caspase Dronc (Meier et al, 2000)) or bantam miRNA (which acts negatively on the hid 3’UTR (Brennecke et al, 2003)) could be two potential target genes of Yorkie that block the death of senescent cells, the finding that effector caspases show high activity in these cells suggests that Yorkie negatively impinges on the apoptotic pathway even at the level of cell death execution. These experimental data contribute to the identification of Yorkie as a potential therapeutic strategy to block CIN-induced tumorigenesis by targeting senescent cells.

### The SASP mediates a tumour-host feed-forward loop: a case of super-competition

In our fly epithelial model of CIN, senescent cells show a remarkable ability to act at a paracrine, autocrine, and systemic level to drive tumour growth, invasiveness, and malignancy, respectively (Joy et al, 2024). They exert their action through elements of the SASP, including mitogenic molecules Wg and Wnt6, Matrix metalloproteinase MMP1, and cytokines such as Upd3. Here we identify a new axis of interaction between senescent cells with the nearby wild type cell population that contribute to tumour growth (Fig. 7H). While the CIN tissue enters a hyperplastic mode of growth in response to the mitogenic molecules Wg and Wnt6 secreted by senescent cells, the nearby wild type cell population shows a reduction in growth rates, which is accompanied by a reduction in proliferative activity and an increase in the number of apoptotic cells. We identified several components of the SASP driving the observed non-autonomous effects: the Relaxin-like molecule Dilp8, the Insulin-like growth factor antagonist ImpL2 (the fly ortholog of IGFBP-7), the Upd1 and Upd3 cytokine and the TNF-α-orthologue Egr. While Dilp8 and ImpL2 were shown to be required and sufficient to drive the non-autonomous reduction in proliferative activity, Upd1, Upd3 and Eiger were found to be responsible for the reduction in growth and increase in apoptotic levels of the neighbouring cell population. We present evidence that Upd1 and Upd3 exerts its action by triggering the JAK/STAT signalling pathway and by inducing cell death. Consistent with the reported activities of Dilp8 and ImpL2 as systemic signals blocking the production or activity of those systemic hormones required for organismal growth [e.g. ecdysone and dILPs, (Figueroa-Clarevega and Bilder, 2015; Kwon et al, 2015; Boulan et al, 2019; Sanchez et al, 2019)], their negative impact on the proliferative activity was found to be extended to the tumour itself and not just to neighbouring tissues. These data expand the repertoire of signalling activities exerted by senescent cells and provide another example of non-cell-autonomous induction of cell death in the tumour host (Katheder et al, 2017). Most interestingly, we present evidence that the non-cell-autonomous induction of cell death induced by Upd cytokines and Eiger is required to feed back to the tumour to enhance its growth, either as a consequence of the liberation of nutrients and metabolites, or the expression of signalling molecules by dying cells before being captured by circulating macrophages (Fig. 7H). These tumour-host interactions parallel interface interactions observed during super-competition, an aggressive form of cell competition where a cell acquiring a specific mutation (often oncogenic) becomes “super-fit” and actively eliminates neighbouring, healthy wild-type cells to expand its territory (Nagata et al, 2022; Moreno and Basler, 2004; Moreno et al, 2019).

## Methods

**Table:** Reagents and tools table

Reagent/Resource	Reference or Source	Identifier or Catalog Number
**Experimental models**		
*ap-GAL4*	Bloomington Drosophila Stock Center	RRID: BDSC_3041
*en-GAL4*	Bloomington Drosophila Stock Center	RRID: BDSC_1973
*UAS-bub3RNAi*	Vienna Drosophila Resource Center	RRID: VDRC_21037
*UAS-rodRNAi*	Vienna Drosophila Resource Center	RRID: VDRC_19152
*UAS-p35*	Bloomington Drosophila Stock Center	RRID: BDSC_5072
*UAS-myristoylated-Tomato (myrT)*	Bloomington Drosophila Stock Center	RRID: BDSC_32222
*UAS-Golgi-GFP*	Bloomington Drosophila Stock Center	RRID: BDSC_30902
*UAS-Sec61β-RFP*	Bloomington Drosophila Stock Center	RRID: BDSC_64747
*UAS-CD63-GFP*	Bloomington Drosophila Stock Center	RRID: BDSC_91390
*UAS-bsk-DN*	Bloomington Drosophila Stock Center	RRID: BDSC_6405
*bantam-lacZ*	Bloomington Drosophila Stock Center	RRID: BDSC_10154
*diap1-GFP4.3* (*diap1-GFP* in the text)	(Zhang et al, 2008)	
*yorkie-GFP*	(Borreguero-Muñoz et al, 2019)	
*Jub-GFP*	Bloomington Drosophila Stock Center	RRID:BDSC_56806
*diap1-lacZ*	(Wu et al, 2003)	
*expanded-lacZ*	(Hamaratoglu et al, 2006)	
*UAS-YkiRNAi*	Vienna Drosophila Resource Center	RRID: VDRC_40497
*UAS-GC3Ai*	Bloomington Drosophila Stock Center	RRID: BDSC_84343
*hid-lacZ*	Bloomington Drosophila Stock Center	RRID: BDSC_38010
*MMP1-GFP*	(Uhlirova and Bohmann, 2006)	
*Df(H99)*	Bloomington Drosophila Stock Center	RRID:BDSC_1576
*UAS-dilp8RNAi*	Vienna Drosophila Resource Center	RRID: VDRC_102604
*UAS-ImpL2RNAi*	Bloomington Drosophila Stock Center	RRID: BDSC_55855
*UAS-dilp8*	(Colombani et al, 2012)	
*UAS-ImpL2*	(Figueroa-Clarevega and Bilder, 2015)	
*dilp8mimicGFP (dilp8-GFP)*	Bloomington Drosophila Stock Center	RRID: BDSC_33079
*impL2mimicGFP (ImpL2-GFP)*	Bloomington Drosophila Stock Center	RRID: BDSC_350068
*UAS-wgRNAi*	Vienna Drosophila Resource Center	RRID: VDRC_104579
*STATGFP10x (II)*	(Bach et al, 2007)	
*UAS-domeDN (III) (Cyt)*	(Brown et al, 2001)	
*UAS-domeRNAi*	Vienna Drosophila Resource Center	RRID: VDRC_106071
*hop 27*	Bloomington Drosophila Stock Center	RRID: BDSC_8493
*egr-lacZ*	(Muzzopappa et al, 2017)	
*UAS-grnd-ext (1)*	(Andersen et al, 2015)	
*UAS-grnd-ext (2)*	(Andersen et al, 2015)	
*UAS-grndRNAi*	Vienna Drosophila Resource Center	RRID: VDRC_104538
*UAS-egrRNAi (1)*	Vienna Drosophila Resource Center	RRID: VDRC_108814
*UAS-egrRNAi (2)*	Gifted by Tatsushi Igaki	-
*3xmCherry-Atg8a*	(Hegedűs et al, 2016)	3xmCherry-Atg8a
**Recombinant DNA**		
**Antibodies**		
Mouse anti-MMP1 (14A3D2))	Developmental Studies Hybridoma bank	RRID: AB_579782
Mouse anti-β-Gal (40.1a)	Developmental Studies Hybridoma bank	RRID: AB_528100
Rabbit anti-c-Dcp-1 (9578S)	Cell Signaling Technology	RRID: AB_2721060
Rat anti-Ci (2A1)	Developmental Studies Hybridoma bank	RRID: AB_2109711
Mouse anti-Nubbin (nub2D4)	Developmental Studies Hybridoma bank	RRID: AB_2722119
Rabbit anti-phospho-Histone H3 (pH3)	Cell Signaling	RRID: AB_331535
Alexa fluor 488-conjugated AffiniPure Donkey Anti-Mouse IgG (H + L)	Jackson ImmunoResearch	RRID: AB_2341099
Cy2 AffiniPure Donkey Anti-Rabbit IgG (H + L)	Jackson ImmunoResearch	RRID: AB_2307385
Cy3 AffiniPure Donkey Anti-Mouse IgG (H + L)	Jackson ImmunoResearch	RRID: AB_2315777
Cy3 AffiniPure Donkey Anti-Rabbit IgG (H + L)	Jackson ImmunoResearch	RRID: AB_2307443
Cy5 AffiniPure Donkey Anti-Rat IgG (H + L)	Jackson ImmunoResearch	RRID: AB_2340671
Donkey anti-Chicken IgY (H + L) Highly Cross Adsorbed Secondary Antibody, Alexa Fluor™ 647	Invitrogen	RRID: AB_2921074
Chicken Anti-beta Galactosidase Polyclonal Antibody, Unconjugated	Abcam	Abcam
**Oligonucleotides and other sequence-based reagents**		
**Chemicals, Enzymes and other reagents**		
Click-iT™ Plus EdU Alexa Fluor™ 647 Imaging Kit	Invitrogen	Code: C10640
DAPI	Sigma-Aldrich	Code: 28718-90-3
In Situ Cell Death Detection Kit, Fluorescein (TUNEL)	Roche	Code:11684 795910
Chloroquine	Sigma-Aldrich	Code: C6628
**Software**		
Fiji	Fiji	https://fiji.sc/
Excel	Microsoft Excel 2021	N/A
GraphPad Prism 10.0.3	GraphPad	RRID:SCR_002798
Biorender	Biorender	https://www.biorender.com
**Other**		

### Fly maintenance, husbandry and transgene expression

Strains of *Drosophila melanogaster* were maintained on standard medium (4% glucose, 55 g/L yeast, 0.65% agar, 28 g/L wheat flour, 4 ml/L propionic acid, and 1.1 g/L nipagin) at 25 °C in light/dark cycles of 12 h. The resulting larvae after the egg laying (AEL) at 25 °C were either transferred to 29 °C, or maintained at 25 °C for specific experiments as mentioned in the figures, and were dissected on the day AEL as mentioned in the figures. The sex of experimental larvae was not considered relevant to this study and was not determined. All strains used in this work are listed in the Reagents and Tools Table.

### Standard induction of CIN, and endogenous labelling of different tissue subpopulations for transcriptomic analysis

Virgin female flies carrying *UAS-myrT, MMP1-GFP* transgenes were crossed with males carrying either *en-GAL4, UAS-p35* (Control) or *en-GAL4, UAS-p35, UAS-rod*^*RNAi*^ (CIN) transgenes. Fertilized females were allowed to lay eggs at 25 °C, and the resulting larvae were switched to 29 °C 24 h AEL. Wing imaginal discs were then dissected after either 5 days (in case of control) or 7 days (in case of CIN) of induction at 29 °C. Wing imaginal discs (40–50 in case of controls, and 30–40 in case of CIN) were cleaned in ice-cold 1X PBS and were incubated in 10X trypsin-EDTA (Sigma-Aldrich) at room temperature (RT) for 1 h, with regular nutation, and gentle pipetting. The single cell suspension was then subjected to centrifugation at 2500 × *g* for 2.5 min. The supernatant was gently removed, and the cells were stained with 1 ml of 1 μg/ml of DAPI solution for 10 min at 4 °C in low-light conditions, after resuspending the cell pellet. The cells were immediately transferred to flow-tubes, and FACS sorting was carried out on the different tissue subpopulations.

### FACS sorting of different tissue subpopulations

Flow cytometry experiments were carried out using a FACS Aria Fusion sorter (Beckton Dickinson, San Jose, California) equipped with a 4-laser configuration (405–488–561–640 nm). Single cells were gated according to scatter parameters, using FSC width to avoid doublets. Dead cells were excluded using DAPI as a viability marker; only live single cells were analysed, and these were sorted on the basis of their fluorescence pattern. For sorting different CIN subpopulations from CIN tissues, P(Aneuploid) cells were distinguished as GFP + RFP+ cells and P(CIN) cells as GFP- RFP+ cells, and the remaining GFP- RFP- cells were labelled as A(Aneuploid) cells. Similarly, for sorting the two subpopulations from the control tissues, RFP+ cells were attributed as P(control), while the RFP- cells were attributed as A(control). Fluorescence of GFP was detected using blue (488 nm) laser excitation and collecting green fluorescence using a 530/30 BP filter. RFP fluorescence was obtained using green-yellow laser excitation (561 nm) and collecting fluorescence using a 610/20 BP filter. A dot plot combining GFP and RFP emission on single live cells was used to select them for sorting into 1.5 ml Eppendorf tubes. A 70 µm nozzle was used to minimize the sorted volume into collection vials. During the entire experiment, the cells were under 300 RPM agitation and were maintained at 4 °C. For each subpopulation, 30,000 cells were collected in RNase-free 1.5 ml microcentrifuge tubes containing 10 µl of freshly prepared 2X Lysis Buffer following the methods from (Gonzalez-Roca et al, 2010).

### RNA purification, library preparation, and bulk RNA-sequencing

Total RNA from a total of 4 replicates of 5 distinct subpopulations was then purified at the IRB Barcelona Functional Genomics Core Facility, as described in (Gonzalez-Roca et al, 2010). mRNA was isolated from total RNA using the NEBNext Poly(A) mRNA Magnetic Isolation Module Kit (New England Biolabs). NGS libraries were prepared from the purified mRNA using the NEBNext Ultra II RNA Library Prep Kit for Illumina (New England Biolabs). A total of 18 cycles of PCR amplification were applied to all libraries. The final libraries were quantified using the Qubit dsDNA HS assay (Invitrogen) and quality controlled with the Bioanalyzer 2100 DNA HS assay (Agilent). An equimolar pool was prepared with the 20 libraries and submitted for sequencing at the *Centro Nacional de Análisis Genómico* (CNAG). A final quality control by qPCR was performed by the sequencing provider before paired-end 150 nt sequencing on a NovaSeq6000 S4 (Illumina). More than 275 Gbp of reads were produced, with a minimum of 39 million paired-end reads per sample.

### Data processing

Adapters from reads were removed using trim galore (v.0.6.7) (https://github.com/FelixKrueger/TrimGalore) with default parameters. Resulting reads were aligned to the dm6 *D. melanogaster* genome version using STAR (v.2.7.10a) (Dobin et al, 2013). The count matrix was generated in R (https://www.R-project.org/) through the function “featureCounts” of the Rsubread package (Liao et al, 2019).

### Differential expression

Differential expression was performed using the DESeq2 package (Love et al, 2014). For plotting purposes and generating principal components, the count matrix was normalized using the “vst” function.

### Gene set enrichment analysis

Gene set enrichment analysis was performed using the roastgsa package (Caballé-Mestres et al, 2023). GO and KEGG pathways were downloaded using the org.Dm.eg.db package.

### Immunohistochemistry

Larvae were selected according to the day AEL mentioned in the individual figures, and wing imaginal discs were dissected in phosphate-buffered saline (PBS), fixed for 20 min in 4% formaldehyde in PBS, and stained with antibodies in PBS with 0.3% BSA, 0.2% Triton X-100. All antibodies and chemicals used in this work are listed in the Reagents and Tools Table.

### DNA synthesis

The Click-iT™ Plus EdU Alexa Fluor™ 647 Imaging Kit from Invitrogen (C10640, see Reagents and Tools Table) was used to measure DNA synthesis (S phase) in proliferating cells, following the manufacturer’s instructions. EdU (5-ethynyl-2´-deoxyuridine) provided in the kit is a nucleoside analogue of thymidine and is incorporated into DNA during active DNA synthesis. Time of incubation with EdU: 15 min.

### TUNEL

Larvae were dissected in cold 1X PBS and fixed in 4% FA for 20 min. After fixation, three fast washes with PBT 0.3% Triton (PBS 1%, Triton X-100 0.3%) followed by four 15-min washes with PBT 0.3% Triton were performed. The samples were blocked with BBT (0.3% BSA, 0.3% Triton X-100) for 1 h rotating at RT and then incubated overnight with the primary antibody at 4 °C. The next day, samples were subjected to three fast washes with PBT 0.3% Triton, and then they were washed four more times with PBT 0.3% Triton for 20 min each, followed by incubation with secondary antibodies diluted in BBT for 90 min rotating in the dark at RT. After incubation, the samples were subjected to three rounds of fast washes with PBT 0.3% Triton and four 20-min washes with PBT 0.3%. Samples were kept in BBT blocking overnight at 4 °C. The following day, samples were permeabilized with 495 μl of 100 mM Na-Citrate and 2.5 μl of 20% TritonX for 30 min at 65 °C and then subjected to three fast washes with PBT 0.3% Triton. They were then incubated twice for 5 min at RT with 50 μl of Equilibration buffer. Then 51 μl of TUNEL reaction mix (50 μl of TUNEL reaction buffer Component A, 1 μl TdT Enzyme, Biotium, see Reagents and Tools Table) was added and the samples were incubated for 2 h at RT. After incubation, the samples were subjected to three fast washes with PBT 0.3% Triton, followed by a 30-min incubation with DAPI at RT. Finally, three fast washes and three 10-min washes with PBT 0.3% Triton were performed. Stained tissues were kept in mounting media.

### Confocal microscopy

Samples were analysed using the following confocal microscopes: STELLARIS Confocal Platform with White Light Laser (Leica Microsystems), LSM 780 Zeiss, and LSM880 Zeiss fitted with a Fast Airyscan module (Carl Zeiss). Z-stacks were acquired using either the laser scanning confocal mode or the High-Resolution mode (Airyscan). Image acquisition was done with 20x, 25x, 40x, 63x, and 100x lenses. The most representative image is shown in all experiments.

### Chloroquine treatment

Flies of desired genotypes were crossed and set up at 25 °C and shifted to 29 °C 8 h AEL. After 48 h at 29 °C, a working concentration of 3 mg/ml of chloroquine (with water as controls, see Reagents and Tools Table) was added to the food. Larvae were processed 72 h thereafter. Experimental flies and control individuals were grown in parallel.

### Tissue size, cell death and mitotic activity

The sizes of the whole wing disc (based on DAPI staining), the anterior (A) and posterior (P) compartments (based on Ci expression, in Figs. 3–5) and the anterior (A) and posterior (P) areas of the wing pouch (labelled by Nubbin expression, in Figs. 6 and 7) were measured manually using Fiji Software (NIH, USA) on apical sections (epithelium) of the wing disc obtained using a Zeiss LSM780 confocal microscope. A and P areas were normalized to the size of the respective control compartments. The left column of each graph represents the genotype which was considered as the control. The area of the signal to be quantified (TUNEL, cDcp-1, EdU, pH3, and mCh-Atg8) was set by manually adjusting the threshold and normalized to the area of the indicated larval territory (A or P compartments within the wing pouch) after making a maximum intensity Z-Projection.

### Statistical analysis

Statistical analysis was performed by either One-way ANOVA when comparing more than two experimental groups or an unpaired equal-variance two-tailed Student’s t-test when comparing the difference in means of a single experimental group with a control. Differences were considered significant when *p* values were less than 0.0001 (****), 0.001 (***), 0.01 (**), or 0.05 (*). All genotypes included in each histogram or scatter plot were subjected to the same experimental conditions (temperature and time of transgene induction) and analysed in parallel. Mean values and standard deviations were calculated, and the corresponding statistical analysis and graphical representations were carried out with GraphPad Prism 10.0 statistical software. No blinding was done.

### Graphics

Figure 7H and synopsis image were created with BioRender.com.

## Supplementary information

Peer Review File

Dataset EV1

Dataset EV2

Dataset EV3

Source data Fig. 1

Source data Fig. 2

Source data Fig. 3

Source data Fig. 4

Source data Fig. 5

Source data Fig. 6

Source data Fig. 7

Expanded View Figures

## Data Availability

Materials generated for the study are available from the corresponding author on request. Source data are provided with this paper. The raw and processed RNAseq data have been deposited in the GEO database under accession number GSE306996. The source data of this paper are collected in the following database record: biostudies:S-SCDT-10_1038-S44319-026-00811-7.

## References

[CR1] Andersen DS, Colombani J, Palmerini V, Chakrabandhu K, Boone E, Rothlisberger M, Toggweiler J, Basler K, Mapelli M, Hueber AO et al (2015) The Drosophila TNF receptor Grindelwald couples loss of cell polarity and neoplastic growth. Nature 522:482–486 25874673 10.1038/nature14298

[CR2] Arquier N, Géminard C, Bourouis M, Jarretou G, Honegger B, Paix A, Léopold P (2008) Drosophila ALS regulates growth and metabolism through functional interaction with insulin-like peptides. Cell Metab 7:333–338 18396139 10.1016/j.cmet.2008.02.003

[CR3] Bach EA, Ekas LA, Ayala-Camargo A, Flaherty MS, Lee H, Perrimon N, Baeg GH (2007) GFP reporters detect the activation of the Drosophila JAK/STAT pathway in vivo. Gene Expr Patterns 7:323–331 17008134 10.1016/j.modgep.2006.08.003

[CR4] Bakhoum SF, Ngo B, Laughney AM, Cavallo J-A, Murphy CJ, Ly P, Shah P, Sriram RK, Watkins TBK, Taunk NK et al (2018) Chromosomal instability drives metastasis through a cytosolic DNA response. Nature 553:467–472 29342134 10.1038/nature25432 PMC5785464

[CR5] Barrio L, Gaspar A-E, Muzzopappa M, Ghosh K, Romao D, Clemente-Ruiz M, Milán M (2023) Chromosomal instability-induced cell invasion through caspase-driven DNA damage. Curr Biol 33:4446–4457.e5 37751744 10.1016/j.cub.2023.09.004

[CR6] Barroso-Vilares M, Macedo JC, Reis M, Warren JD, Compton D, Logarinho E (2020) Small-molecule inhibition of aging-associated chromosomal instability delays cellular senescence. EMBO Rep 21:e49248 32134180 10.15252/embr.201949248 PMC7202060

[CR7] Benhra N, Barrio L, Muzzopappa M, Milán M (2018) Chromosomal instability induces cellular invasion in epithelial tissues. Dev Cell 47:161–174.e4 30245154 10.1016/j.devcel.2018.08.021

[CR8] Borreguero-Muñoz N, Fletcher GC, Aguilar-Aragon M, Elbediwy A, Vincent-Mistiaen ZI, Thompson BJ (2019) The Hippo pathway integrates PI3K–Akt signals with mechanical and polarity cues to control tissue growth. PLOS Biol 17:e3000509 31613895 10.1371/journal.pbio.3000509 PMC6814241

[CR9] Boulan L, Andersen D, Colombani J, Boone E, Léopold P (2019) Inter-organ growth coordination is mediated by the Xrp1-Dilp8 axis in Drosophila. Dev Cell 49:811–818.e4 31006647 10.1016/j.devcel.2019.03.016

[CR10] Brennecke J, Hipfner DR, Stark A, Russell RB, Cohen SM (2003) bantam encodes a developmentally regulated microRNA that controls cell proliferation and regulates the proapoptotic gene hid in Drosophila. Cell 113:25–36 12679032 10.1016/s0092-8674(03)00231-9

[CR11] Brown S, Hu N, Hombria JC (2001) Identification of the first invertebrate interleukin JAK/STAT receptor, the Drosophila gene domeless. Curr Biol 11:1700–1705 11696329 10.1016/s0960-9822(01)00524-3

[CR12] Caballé-Mestres A, Berenguer-Llergo A, Stephan-Otto Attolini C (2023) Roastgsa: a comparison of rotation-based scores for gene set enrichment analysis. BMC Bioinforma 24:408 10.1186/s12859-023-05510-x PMC10617084 37904108

[CR13] Clemente-Ruiz M, Murillo-Maldonado JM, Benhra N, Barrio L, Perez L, Quiroga G, Nebreda AR, Milan M, Pérez L, Quiroga G et al (2016) Gene dosage imbalance contributes to chromosomal instability-induced tumorigenesis. Dev Cell 36:290–302 26859353 10.1016/j.devcel.2016.01.008

[CR14] Clemente-Ruiz M, Muzzopappa M, Milán M, Milan M (2014) Tumor suppressor roles of CENP-E and Nsl1 in Drosophila epithelial tissues. Cell Cycle 13:1450–1455 24626182 10.4161/cc.28417 PMC4050142

[CR15] Colombani J, Andersen DSDS, Leopold P, Léopold P (2012) Secreted peptide Dilp8 coordinates Drosophila tissue growth with developmental timing. Science 336:582–585 22556251 10.1126/science.1216689

[CR16] Das Thakur M, Feng Y, Jagannathan R, Seppa MJ, Skeath JB, Longmore GD (2010) Ajuba LIM proteins are negative regulators of the Hippo signaling pathway. Curr Biol 20:657–662 20303269 10.1016/j.cub.2010.02.035 PMC2855397

[CR17] Davoli T, Xu AW, Mengwasser KE, Sack LM, Yoon JC, Park PJ, Elledge SJ (2013) Cumulative haploinsufficiency and triplosensitivity drive aneuploidy patterns and shape the cancer genome. Cell 155:948–962 24183448 10.1016/j.cell.2013.10.011 PMC3891052

[CR18] Dekanty A, Barrio L, Muzzopappa M, Auer H, Milan M (2012) Aneuploidy-induced delaminating cells drive tumorigenesis in Drosophila epithelia. Proc Natl Acad Sci 109:20549–20554 23184991 10.1073/pnas.1206675109 PMC3528526

[CR19] Dobin A, Davis CA, Schlesinger F, Drenkow J, Zaleski C, Jha S, Batut P, Chaisson M, Gingeras TR (2013) STAR: ultrafast universal RNA-seq aligner. Bioinformatics 29:15–21 23104886 10.1093/bioinformatics/bts635 PMC3530905

[CR20] Figueroa-Clarevega A, Bilder D (2015) Malignant Drosophila tumors interrupt insulin signaling to induce cachexia-like wasting. Dev Cell 33:47–55 25850672 10.1016/j.devcel.2015.03.001 PMC4390765

[CR21] Fusari E, Muzzopappa M, Gracia J, Milán M (2025) Depletion of aneuploid cells is shaped by cell-to-cell interactions. Cell Genomics 5:100894 40466636 10.1016/j.xgen.2025.100894 PMC12366656

[CR22] Garelli A, Gontijo AM, Miguela V, Caparros E, Dominguez M (2012) Imaginal discs secrete insulin-like peptide 8 to mediate plasticity of growth and maturation. Science 336:579–582 22556250 10.1126/science.1216735

[CR23] Godek KM, Venere M, Wu Q, Mills KD, Hickey WF, Rich JN, Compton DA (2016) Chromosomal instability affects the tumorigenicity of glioblastoma tumor-initiating cells. Cancer Discov 6:532–545 27001151 10.1158/2159-8290.CD-15-1154 PMC4854766

[CR24] Gonzalez-Roca E, Garcia-Albéniz X, Rodriguez-Mulero S, Gomis RR, Kornacker K, Auer H (2010) Accurate expression profiling of very small cell populations. PLoS ONE 5:e14418 21203435 10.1371/journal.pone.0014418 PMC3010985

[CR25] Gorgoulis V, Adams PD, Alimonti A, Bennett DC, Bischof O, Bishop C, Campisi J, Collado M, Evangelou K, Ferbeyre G et al (2019) Cellular senescence: defining a path forward. Cell 179:813–827 31675495 10.1016/j.cell.2019.10.005

[CR26] Gracia-Latorre E, Pérez L, Muzzopappa M, Milán M (2022) A single WNT enhancer drives specification and regeneration of the Drosophila wing. Nat Commun 13:4794 35995781 10.1038/s41467-022-32400-2 PMC9395397

[CR27] Hamaratoglu F, Willecke M, Kango-Singh M, Nolo R, Hyun E, Tao C, Jafar-Nejad H, Halder G (2006) The tumour-suppressor genes NF2/Merlin and Expanded act through Hippo signalling to regulate cell proliferation and apoptosis. Nat Cell Biol 8:27–36 16341207 10.1038/ncb1339

[CR28] Hay BA, Wolff T, Rubin GM (1994) Expression of baculovirus P35 prevents cell death in Drosophila. Development 120:2121–2129 7925015 10.1242/dev.120.8.2121

[CR29] Hegedűs K, Takáts S, Boda A, Jipa A, Nagy P, Varga K, Kovács AL, Juhász G (2016) The Ccz1-Mon1-Rab7 module and Rab5 control distinct steps of autophagy. Mol Biol Cell 27:3132–3142 27559127 10.1091/mbc.E16-03-0205 PMC5063620

[CR30] Honegger B, Galic M, Köhler K, Wittwer F, Brogiolo W, Hafen E, Stocker H (2008) Imp-L2, a putative homolog of vertebrate IGF-binding protein 7, counteracts insulin signaling in Drosophila and is essential for starvation resistance. J Biol 7:10 18412985 10.1186/jbiol72 PMC2323038

[CR31] Ippolito MR, Martis V, Martin S, Tijhuis AE, Hong C, Wardenaar R, Dumont M, Zerbib J, Spierings DCJJ, Fachinetti D et al (2021) Gene copy-number changes and chromosomal instability induced by aneuploidy confer resistance to chemotherapy. Dev Cell 56:2440–2454.e6 34352223 10.1016/j.devcel.2021.07.006

[CR32] Joy J, Barrio L, Santos-Tapia C, Romão D, Giakoumakis NN, Clemente-Ruiz M, Milán M (2021) Proteostasis failure and mitochondrial dysfunction leads to aneuploidy-induced senescence. Dev Cell 56:2043–2058.e7 34216545 10.1016/j.devcel.2021.06.009

[CR83] Joy J, Fusari E, Milán M (2024) Aneuploidy-induced cellular behaviors: Insights from *Drosophila*. Dev Cell 59:295-307 38320484 10.1016/j.devcel.2023.12.009

[CR33] Kanda H, Igaki T, Okano H, Miura M (2011) Conserved metabolic energy production pathways govern Eiger/TNF-induced nonapoptotic cell death. Proc Natl Acad Sci USA 108:18977–18982 22065747 10.1073/pnas.1103242108 PMC3223446

[CR34] Katheder NS, Khezri R, O’Farrell F, Schultz SW, Jain A, Rahman MM, Schink KO, Theodossiou TA, Johansen T, Juhasz G et al (2017) Microenvironmental autophagy promotes tumour growth. Nature 541:417–420 28077876 10.1038/nature20815 PMC5612666

[CR35] Kowalczyk W, Romanelli L, Atkins M, Hillen H, Bravo González-Blas C, Jacobs J, Xie J, Soheily S, Verboven E, Moya IM et al (2022) Hippo signaling instructs ectopic but not normal organ growth. Science 378:eabg3679 10.1126/science.abg3679 36395225

[CR36] Kwon Y, Song W, Droujinine IA, Hu Y, Asara JM, Perrimon N (2015) Systemic organ wasting induced by localized expression of the secreted insulin/IGF antagonist ImpL2. Dev Cell 33:36–46 25850671 10.1016/j.devcel.2015.02.012 PMC4437243

[CR37] Lee AJX, Endesfelder D, Rowan AJ, Walther A, Birkbak NJ, Futreal PA, Downward J, Szallasi Z, Tomlinson IPM, Howell M et al (2011) Chromosomal instability confers intrinsic multidrug resistance. Cancer Res 71:1858–1870 21363922 10.1158/0008-5472.CAN-10-3604 PMC3059493

[CR38] Levine MS, Bakker B, Boeckx B, Moyett J, Lu J, Vitre B, Spierings DC, Lansdorp PM, Cleveland DW, Lambrechts D et al (2017) Centrosome amplification is sufficient to promote spontaneous tumorigenesis in mammals. Dev Cell 40:313–322.e5 28132847 10.1016/j.devcel.2016.12.022 PMC5296221

[CR39] Li J, Duran MA, Dhanota N, Chatila WK, Bettigole SE, Kwon J, Sriram RK, Humphries MP, Salto-Tellez M, James JA et al (2021) Metastasis and immune evasion from extracellular cGAMP hydrolysis. Cancer Discov 11:1212–1227 33372007 10.1158/2159-8290.CD-20-0387 PMC8102348

[CR40] Li J, Hubisz MJ, Earlie EM, Duran MA, Hong C, Varela AA, Lettera E, Deyell M, Tavora B, Havel JJ et al (2023) Non-cell-autonomous cancer progression from chromosomal instability. Nature 620:1080–1088 37612508 10.1038/s41586-023-06464-z PMC10468402

[CR41] Li R, Zhu J (2022) Effects of aneuploidy on cell behaviour and function. Nat Rev Mol Cell Biol 23:250–265 10.1038/s41580-021-00436-9 34987171

[CR42] Liao Y, Smyth GK, Shi W (2019) The R package Rsubread is easier, faster, cheaper and better for alignment and quantification of RNA sequencing reads. Nucleic Acids Res 47:e47 30783653 10.1093/nar/gkz114 PMC6486549

[CR43] Love MI, Huber W, Anders S (2014) Moderated estimation of fold change and dispersion for RNA-seq data with DESeq2. Genome Biol 15:550 25516281 10.1186/s13059-014-0550-8 PMC4302049

[CR44] Lukow DA, Sausville EL, Suri P, Chunduri NK, Wieland A, Leu J, Smith JC, Girish V, Kumar AA, Kendall J et al (2021) Chromosomal instability accelerates the evolution of resistance to anti-cancer therapies. Dev Cell 56:2427–2439.e4 34352222 10.1016/j.devcel.2021.07.009 PMC8933054

[CR45] Macedo JC, Vaz S, Bakker B, Ribeiro R, Bakker PL, Escandell JM, Ferreira MG, Medema R, Foijer F, Logarinho E (2018) FoxM1 repression during human aging leads to mitotic decline and aneuploidy-driven full senescence. Nat Commun 9:2834 30026603 10.1038/s41467-018-05258-6 PMC6053425

[CR46] Martin N, Popgeorgiev N, Ichim G, Bernard D (2023) BCL-2 proteins in senescence: beyond a simple target for senolysis? Nat Rev Mol Cell Biol 24:517–518 36869150 10.1038/s41580-023-00594-y

[CR47] Meier P, Silke J, Leevers SJ, Evan GI (2000) The Drosophila caspase DRONC is regulated by DIAP1. EMBO J 19:598–611 10675329 10.1093/emboj/19.4.598 PMC305598

[CR48] Monserrat J, Morales Torres C, Richardson L, Wilson TS, Patel H, Domart M-C, Horswell S, Song O-R, Jiang M, Crawford M et al (2021) Disruption of the MSL complex inhibits tumour maintenance by exacerbating chromosomal instability. Nat Cell Biol 23:401–412 33837287 10.1038/s41556-021-00657-2 PMC7610593

[CR49] Morais da Silva S, Moutinho-Santos T, Sunkel CE (2013) A tumor suppressor role of the Bub3 spindle checkpoint protein after apoptosis inhibition. J Cell Biol 201:385–393 23609535 10.1083/jcb.201210018 PMC3639401

[CR50] Moreno E, Basler K (2004) dMyc transforms cells into super-competitors. Cell 117:117–129 15066287 10.1016/s0092-8674(04)00262-4

[CR51] Moreno E, Valon L, Levillayer F, Levayer R (2019) Competition for space induces cell elimination through compaction-driven ERK downregulation. Curr Biol 29:23–34.e8 30554899 10.1016/j.cub.2018.11.007 PMC6331351

[CR52] Muzzopappa M, Murcia L, Milan M, Milán M, Milan M (2017) Feedback amplification loop drives malignant growth in epithelial tissues. Proc Natl Acad Sci USA 114:E7291–E7300 28808034 10.1073/pnas.1701791114 PMC5584413

[CR53] Nagata R, Akai N, Kondo S, Saito K, Ohsawa S, Igaki T (2022) Yorkie drives supercompetition by non-autonomous induction of autophagy via bantam microRNA in Drosophila. Curr Biol 32:1064–1076.e4 35134324 10.1016/j.cub.2022.01.016

[CR54] Nguyen B, Fong C, Luthra A, Smith SA, DiNatale RG, Nandakumar S, Walch H, Chatila WK, Madupuri R, Kundra R et al (2022) Genomic characterization of metastatic patterns from prospective clinical sequencing of 25,000 patients. Cell 185:563–575.e11 35120664 10.1016/j.cell.2022.01.003 PMC9147702

[CR55] Nolo R, Morrison CM, Tao C, Zhang X, Halder G (2006) The bantam microRNA is a target of the hippo tumor-suppressor pathway. Curr Biol 16:1895–1904 16949821 10.1016/j.cub.2006.08.057

[CR56] Rauskolb C, Sun S, Sun G, Pan Y, Irvine KD (2014) Cytoskeletal tension inhibits Hippo signaling through an Ajuba-Warts complex. Cell 158:143–156 24995985 10.1016/j.cell.2014.05.035 PMC4082802

[CR57] Recasens-Alvarez C, Ferreira A, Milán M, Milan M (2017) JAK/STAT controls organ size and fate specification by regulating morphogen production and signalling. Nat Commun 8:13815 28045022 10.1038/ncomms13815 PMC5216089

[CR58] Romão D, Muzzopappa M, Barrio L, Milán M (2021) The Upd3 cytokine couples inflammation to maturation defects in Drosophila. Curr Biol 31:1780–1787.e6 33609452 10.1016/j.cub.2021.01.080

[CR59] Rowald K, Mantovan M, Passos J, Buccitelli C, Mardin BR, Korbel JO, Jechlinger M, Sotillo R (2016) Negative selection and chromosome instability induced by Mad2 overexpression delay breast cancer but facilitate oncogene-independent outgrowth. Cell Rep 15:2679–2691 27292643 10.1016/j.celrep.2016.05.048 PMC4920917

[CR60] Sanchez JA, Mesquita D, Ingaramo MC, Ariel F, Milán M, Dekanty A (2019) Eiger/TNFα-mediated Dilp8 and ROS production coordinate intra-organ growth in Drosophila. PLOS Genet 15:e1008133 31425511 10.1371/journal.pgen.1008133 PMC6715248

[CR61] Santaguida S, Amon A (2015) Short- and long-term effects of chromosome mis-segregation and aneuploidy. Nat Rev Mol Cell Biol 16:473–485 26204159 10.1038/nrm4025

[CR62] Santaguida S, Richardson A, Iyer DR, M’Saad O, Zasadil L, Knouse KA, Wong YL, Rhind N, Desai A, Amon A (2017) Chromosome mis-segregation generates cell-cycle-arrested cells with complex karyotypes that are eliminated by the immune system. Dev Cell 41:638–651.e5 28633018 10.1016/j.devcel.2017.05.022 PMC5536848

[CR63] Santaguida S, Vasile E, White E, Amon A (2015) Aneuploidy-induced cellular stresses limit autophagic degradation. Genes Dev 29:2010–2021 26404941 10.1101/gad.269118.115 PMC4604343

[CR64] Schott S, Ambrosini A, Barbaste A, Benassayag C, Gracia M, Proag A, Rayer M, Monier B, Suzanne M (2017) A fluorescent toolkit for spatiotemporal tracking of apoptotic cells in living Drosophila tissues. Development 144:3840–3846 28870988 10.1242/dev.149807

[CR65] Siegrist SE, Haque NS, Chen CH, Hay BA, Hariharan IK (2010) Inactivation of both Foxo and Reaper promotes long-term adult neurogenesis in Drosophila. Curr Biol 20:643–648 20346676 10.1016/j.cub.2010.01.060 PMC2862284

[CR66] Silk AD, Zasadil LM, Holland AJ, Vitre B, Cleveland DW, Weaver BA (2013) Chromosome missegregation rate predicts whether aneuploidy will promote or suppress tumors. Proc Natl Acad Sci USA 110:E4134–E4141 24133140 10.1073/pnas.1317042110 PMC3816416

[CR67] Sluss HK, Han Z, Barrett T, Goberdhan DC, Wilson C, Davis RJ, Ip YT (1996) A JNK signal transduction pathway that mediates morphogenesis and an immune response in Drosophila. Genes Dev 10:2745–2758 8946915 10.1101/gad.10.21.2745

[CR68] Song W, Kir S, Hong S, Hu Y, Wang X, Binari R, Tang H-W, Chung V, Banks AS, Spiegelman B et al (2019) Tumor-derived ligands trigger tumor growth and host wasting via differential MEK activation. Dev Cell 48:277–286.e6 30639055 10.1016/j.devcel.2018.12.003 PMC6368352

[CR69] Stephens PJ, Greenman CD, Fu B, Yang F, Bignell GR, Mudie LJ, Pleasance ED, Lau KW, Beare D, Stebbings LA et al (2011) Massive genomic rearrangement acquired in a single catastrophic event during cancer development. Cell 144:27–40 21215367 10.1016/j.cell.2010.11.055 PMC3065307

[CR70] Stingele S, Stoehr G, Storchova Z (2013) Activation of autophagy in cells with abnormal karyotype. Autophagy 9:246–248 23108329 10.4161/auto.22558 PMC3552891

[CR71] Sun G, Irvine KD (2013) Ajuba family proteins link JNK to Hippo signaling. Sci Signal 6:ra81 24023255 10.1126/scisignal.2004324 PMC3830546

[CR72] Tenev T, Zachariou A, Wilson R, Ditzel M, Meier P (2005) IAPs are functionally non-equivalent and regulate effector caspases through distinct mechanisms. Nat Cell Biol 7:70–77 15580265 10.1038/ncb1204

[CR73] Thompson BJ, Cohen SM (2006) The Hippo pathway regulates the bantam microRNA to control cell proliferation and apoptosis in Drosophila. Cell 126:767–774 16923395 10.1016/j.cell.2006.07.013

[CR74] Thompson SL, Compton DA (2010) Proliferation of aneuploid human cells is limited by a p53-dependent mechanism. J Cell Biol 188:369–381 20123995 10.1083/jcb.200905057 PMC2819684

[CR75] Uhlirova M, Bohmann D (2006) JNK- and Fos-regulated Mmp1 expression cooperates with Ras to induce invasive tumors in Drosophila. EMBO J 25:5294–5304 17082773 10.1038/sj.emboj.7601401 PMC1636619

[CR76] van Dijk E, van den Bosch T, Lenos KJ, El Makrini K, Nijman LE, van Essen HFB, Lansu N, Boekhout M, Hageman JH, Fitzgerald RC et al (2021) Chromosomal copy number heterogeneity predicts survival rates across cancers. Nat Commun 12:3188 34045449 10.1038/s41467-021-23384-6 PMC8160133

[CR77] Wang B, Han J, Elisseeff JH, Demaria M (2024) The senescence-associated secretory phenotype and its physiological and pathological implications. Nat Rev Mol Cell Biol 25:958–978 38654098 10.1038/s41580-024-00727-x

[CR78] Wang RW, Viganò S, Ben-David U, Amon A, Santaguida S (2021) Aneuploid senescent cells activate NF-κB to promote their immune clearance by NK cells. EMBO Rep 22:e52032 34105235 10.15252/embr.202052032 PMC8339690

[CR79] Weaver BAA, Silk AD, Montagna C, Verdier-Pinard P, Cleveland DW (2007) Aneuploidy acts both oncogenically and as a tumor suppressor. Cancer Cell 11:25–36 17189716 10.1016/j.ccr.2006.12.003

[CR80] Wu S, Huang J, Dong J, Pan D (2003) Hippo encodes a Ste-20 family protein kinase that restricts cell proliferation and promotes apoptosis in conjunction with Salvador and Warts. Cell 114:445–456 12941273 10.1016/s0092-8674(03)00549-x

[CR81] Zhang L, Ren F, Zhang Q, Chen Y, Wang B, Jiang J (2008) The TEAD/TEF family of transcription factor scalloped mediates Hippo signaling in organ size control. Dev Cell 14:377–387 18258485 10.1016/j.devcel.2008.01.006 PMC2292673

[CR82] Zhu J, Tsai H-J, Gordon MR, Li R (2018) Cellular stress associated with aneuploidy. Dev Cell 44:420–431 29486194 10.1016/j.devcel.2018.02.002 PMC6529225

