# Supplementary material for: A tumour-host feed-forward loop contributes to the growth of chromosomal instability-induced tumours
Source: EMBO Rep. 2026 Jun 5;27(16):4694–720. doi: 10.1038/s44319-026-00811-7 (PMC13503916; doi:10.1038/s44319-026-00811-7)
Supplement: Supplementary file 1 [file 44319_2026_811_MOESM12_ESM.pdf]

Expanded View Figures

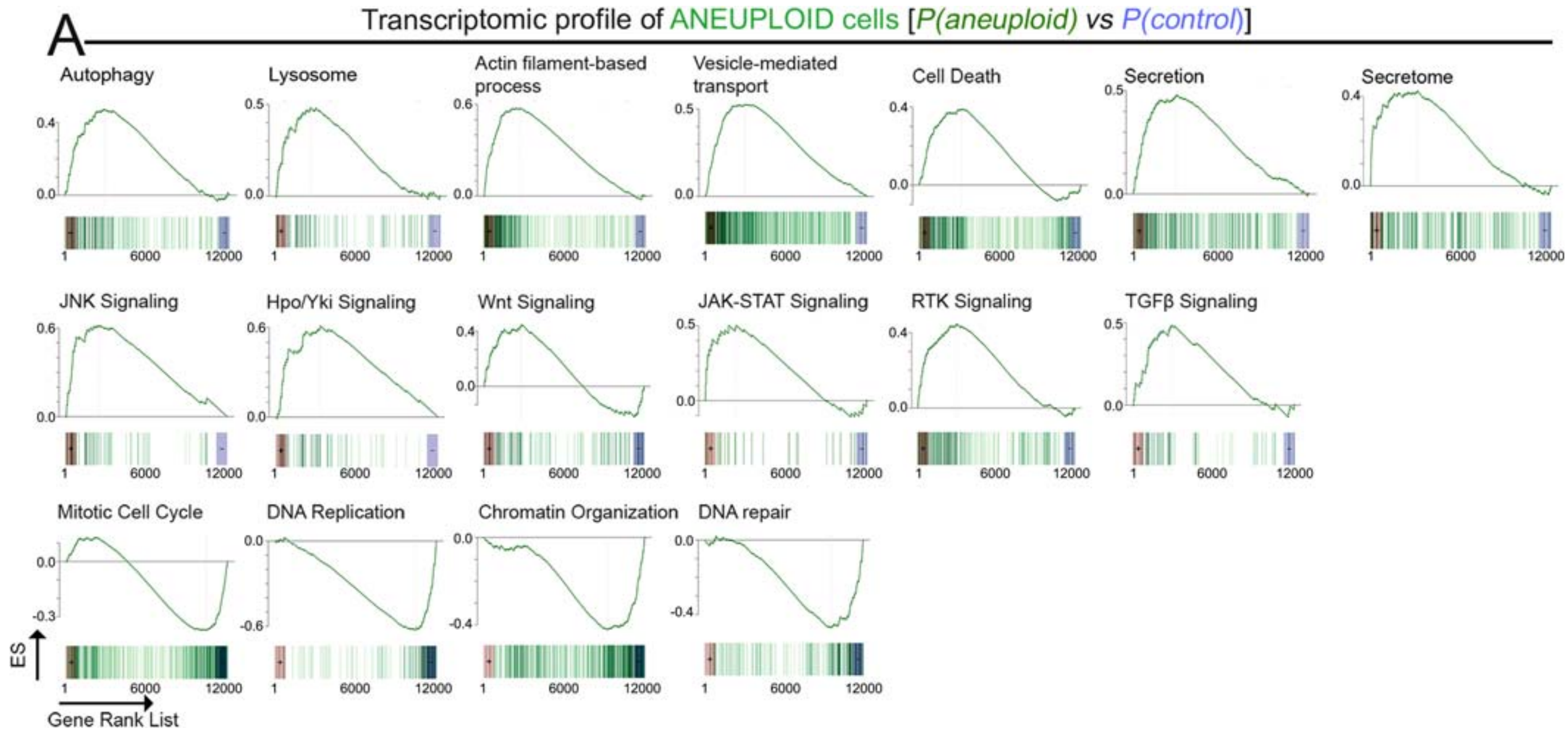

Figure EV1. Transcriptomic landscape of aneuploidy-induced senescent cells (related to Fig. 1).

(**A**) Mountain plots representing Enrichment Score (ES) upon Gene Set Enrichment Analysis (GSEA) comparing P(Aneuploid) with P(control) cells. Top row - up-regulated cellular processes: Autophagy - GO0006914, Lysosome - GO0005764, Actin filament-based process - GO0030029, Vesicle-mediated transport - GO0016192, Cell death - GO0008219, Secretion - GO0046903, Secretome - Custom Gene Set. Middle row - up-regulated signalling pathways: JNK Signalling - GO0007254, Hpo/Yki Signalling - GO0035329, Wnt Signalling - GO0198738, JAK-STAT Signalling - GO0097696, RTK Signalling - GO0007169, TGFβ Signalling - GO0030509. Bottom row - down-regulated cellular processes: Mitotic cell cycle - GO0000278, DNA Replication - GO0006260, Chromatin Organization - GO0006325, DNA repair - GO0006281. See also Dataset EV1.

 © The Author(s)

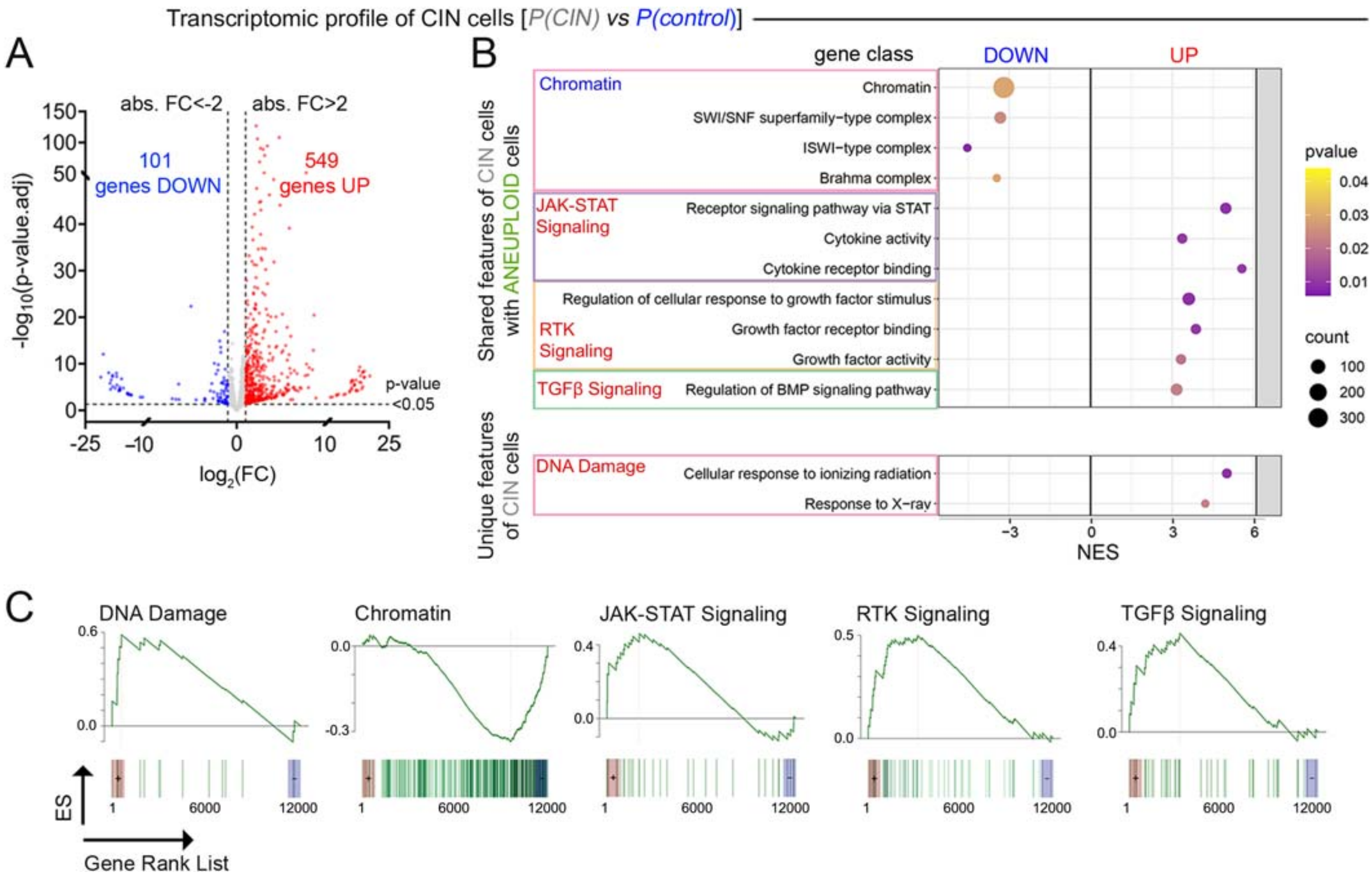

Figure EV2. Transcriptomic landscape of proliferating epithelium subjected to CIN (related to Fig. 1).

(**A**) Volcano plot representing all genes that are differentially up- (red dots, FC > 2 and p-value < 0.05) or down-regulated (blue dots, FC < −2 and p-value < 0.05), or not differentially expressed (grey dots, p-value > 0.05 or 2 > FC > −2) in proliferating cells subjected to CIN when compared to P cells of control discs. Fold changes and p-values were calculated using the DESeq2 R package. p-values were adjusted for multiple comparisons with the Benjamini-Hochberg algorithm. (**B**) Bubble plot representing Gene Ontology (GO) enrichment analyses of proliferating epithelium subjected to CIN when compared to P cells of control discs. On the top, GOs shared with aneuploid cells. On the bottom, those GOs uniquely upregulated in the proliferating epithelium subjected to CIN. Statistics from the roastgsa enrichment algorithm of selected pathways are shown, where X axis corresponds to the normalized enrichment score (NES), size of the bubble represents the number of measured genes and colour-scale represents the associated p-value. See the methods section for details on methodology. (**C**) Mountain plots representing Enrichment Score (ES) upon Gene Set Enrichment Analysis (GSEA) of proliferating cells subjected to CIN when compared to P cells of control discs. GO classes: DNA Damage - GO0071479, Chromatin - GO0000785, JAK-STAT Signalling - GO0097696, RTK Signalling - GO0090287, TGFβ Signalling - GO0030510. n (number of technical replicates per genotype) = 4. See also Dataset EV1.

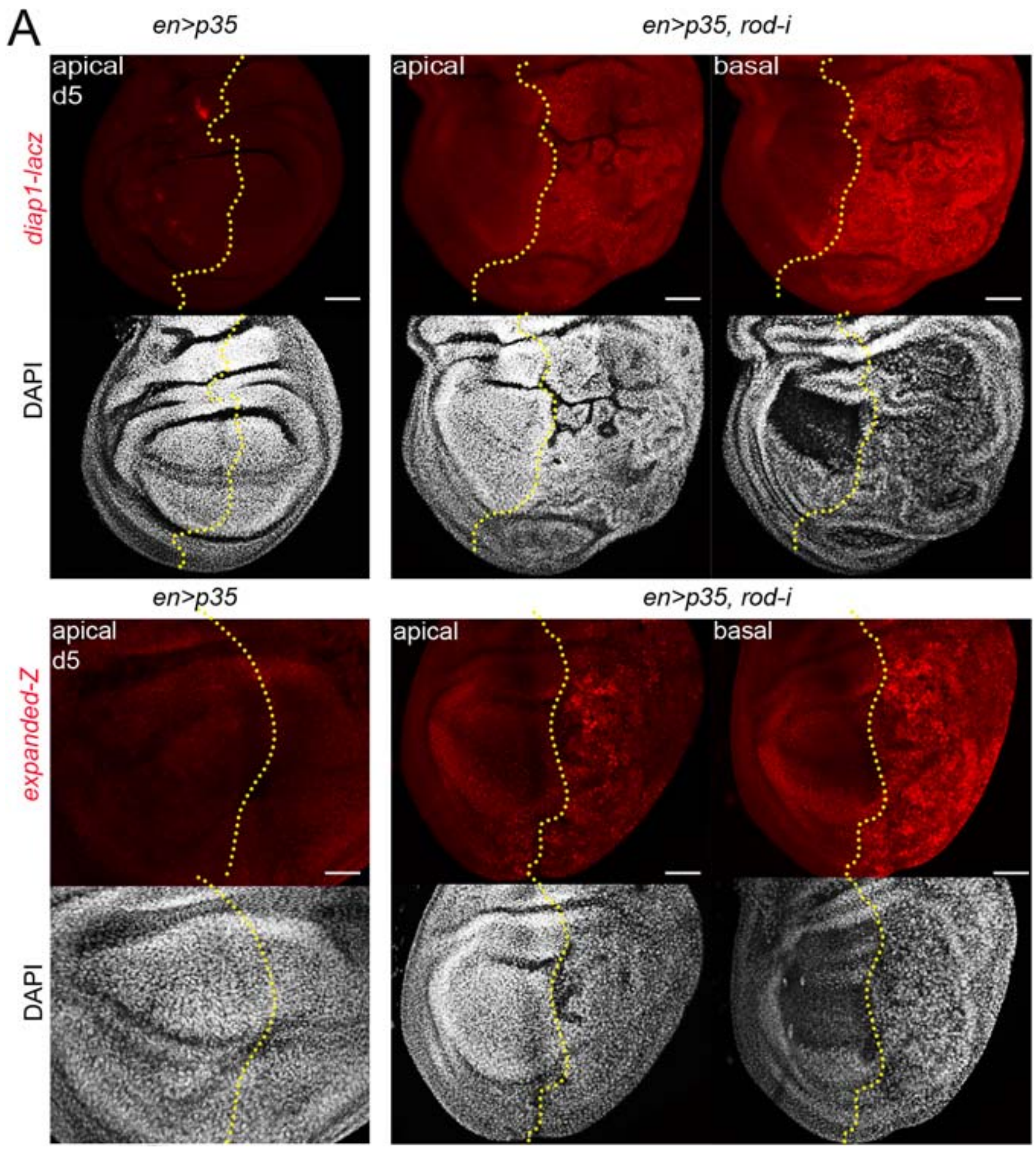

Figure EV3. Yorkie targets are activated in aneuploidy-induced senescent cells (related to Fig. 3).

(**A**) Control wing discs (first column) and wing discs subjected to CIN (remaining columns), stained to visualize *DIAP1-lacZ* (red, **A**), *expanded-lacZ* (red, **A**), and DAPI (white in **A**), and expressing the indicated transgenes in the P compartment under the control of the *en-gal4* driver. Scale bars, 50 µm. Discs were dissected 5 days AEL.

 © The Author(s)

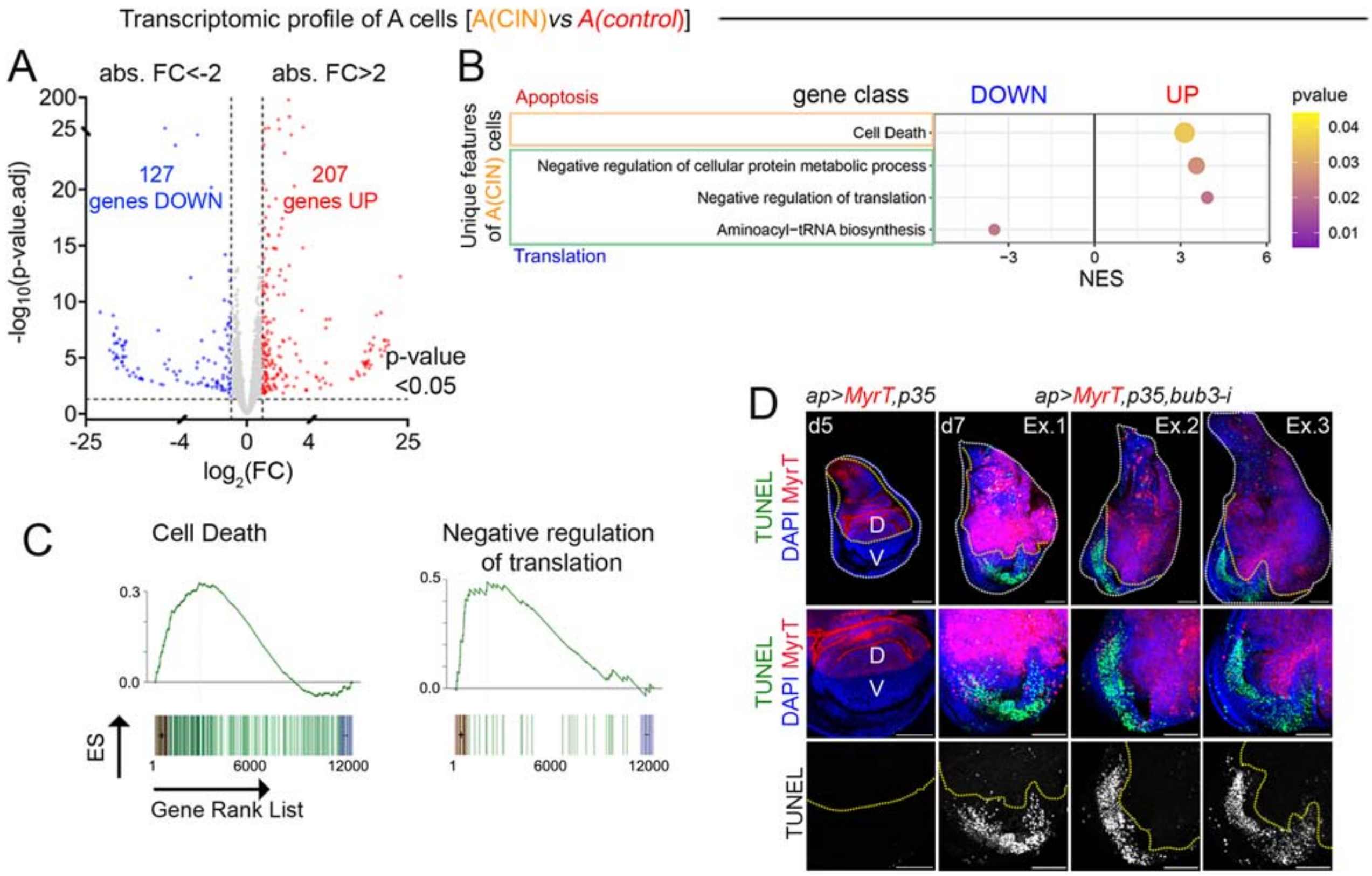

Figure EV4. A non-autonomous effect of CIN tumours on the transcriptomic landscape of the neighbouring tissue (related to Fig. 3).

(**A**) Volcano plot of all genes that are non-autonomously up- (red dots, FC > 2 and *p*-value < 0.05) or down-regulated (blue dots, FC < −2 and *p*-value < 0.05), or not differentially expressed (grey dots, *p*-value > 0.05 or 2 > FC > −2) in the A compartment of wing discs bearing CIN tumours in the P compartment when compared to A cells of control discs. Fold changes and *p*-values were calculated using the DESeq2 R package. *p*-values were adjusted for multiple comparisons with the Benjamini-Hochberg algorithm. (**B**) Bubble plot representing Gene Ontology (GO) enrichment analyses of the A compartment of wing discs bearing CIN tumours in the P compartment when compared to P cells of control discs. Statistics from the roastgsa enrichment algorithm of selected pathways are shown, where X axis corresponds to the normalized enrichment score (NES), size of the bubble represents the number of measured genes and colour-scale represents the associated *p*-value. See the methods section for details on methodology. In (**A**, **B**), *n* (number of technical replicates per genotype) = 4. (**C**) Mountain plots representing Enrichment Score (ES) upon Gene Set Enrichment Analysis (GSEA) of the A compartment of wing discs bearing CIN tumours in the P compartment when compared to A cells of control discs. GO classes: Cell death - GO0008219, Negative regulation of translation - GO0017148. (**D**) Control wing disc (first column) and three examples of wing discs subjected to CIN (remaining columns), and expressing the indicated transgenes in the dorsal (D) compartment under the control of the *ap-gal4* driver, stained for MyrT (red), TUNEL (green or white) and DAPI (blue). Scale bars, 50 µm. Days of dissection after egg laying are as indicated.

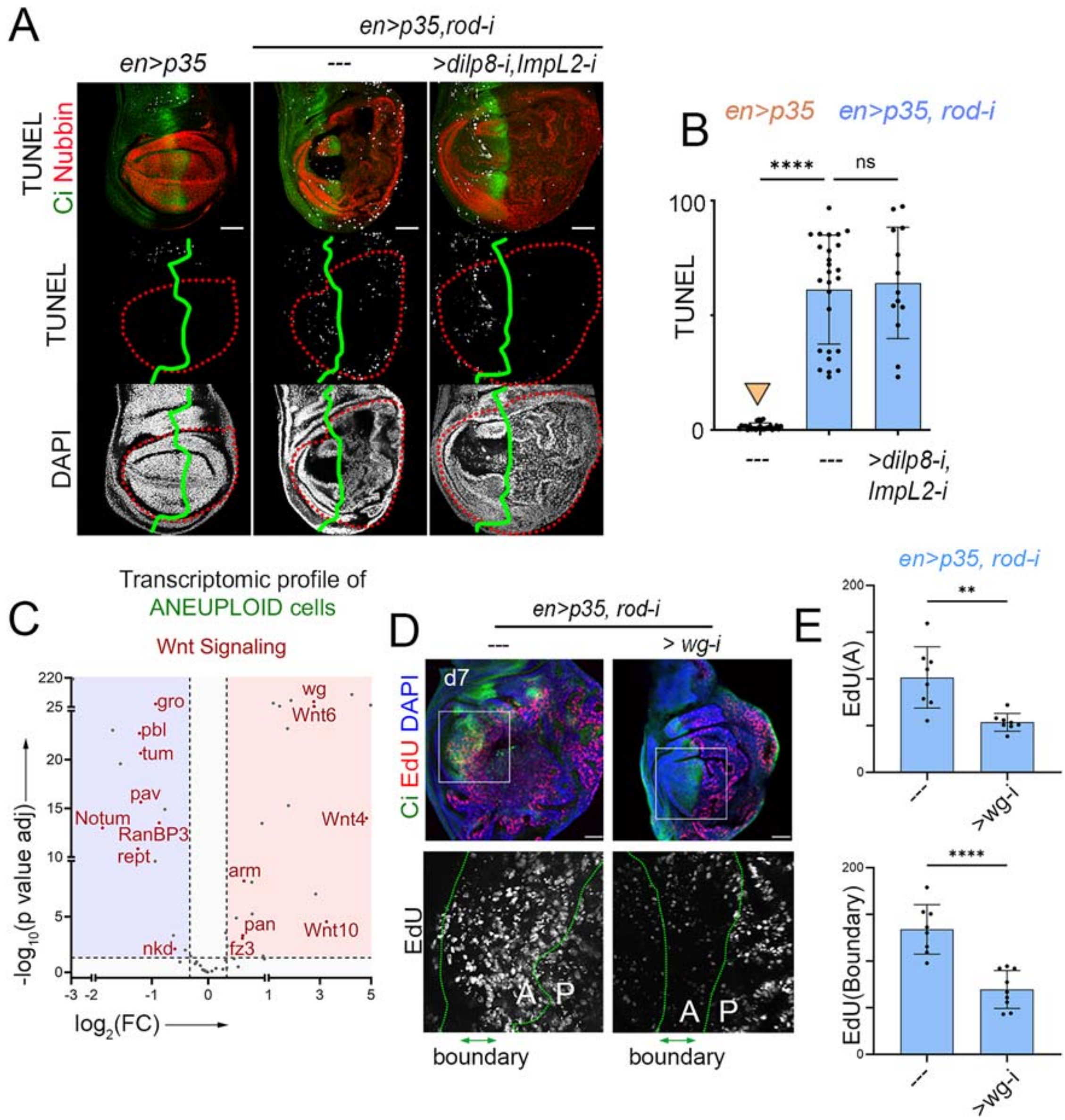

Figure EV5. Contribution of Dilp8/Relaxin, ImpL2/IGFBP7 and Wingless to the non-autonomous effects of CIN tumours on cell death and proliferation (related to Fig. 6).

(**A**) Wing discs subjected to CIN, stained to visualize TUNEL (white), DAPI (white), Nubbin (red, to label the wing pouch cells), Ci (green, to label the A compartment), and expressing the indicated transgenes in the P compartment under the control of the *en-gal4* driver. The boundary between A and P cells is labelled by a green line. (**B**, **E**) Histograms plotting the amount of TUNEL (**B**) or EdU signal (**E**) in the A wing pouch compartments or wing pouch AP boundary (normalized to the size of the corresponding territories) of wing discs expressing the indicated transgenes in the P compartment under the control of the *en-gal4* driver. Graphs represent mean and standard deviation. $^*p < 0.05$, $^{**}p < 0.01$, $^{***}p < 0.001$, $^{****}p < 0.0001$, ns, not significant. One-way ANOVA with Šídák's multiple comparisons test (**B**) and Unpaired t test (**E**), n = number of biological replicates. Sample size and p value: (**B**, TUNEL (A)): *en > p35* ($n = 26$) vs *en > p35, rod-i* ($n = 26$) ($p = 6.71 \times 10^{-17}$); *en > p35, rod-i* ($n = 26$) *vs en > p35, rod-i, dilp8-i, ImpL2-i* ($n = 13$) ($p = 0.87$); (**E**, EdU (A)): *en > p35, rod-i* ($n = 8$) vs *en > p35, rod-i, wg-i* ($n = 8$) ($p = 1.37 \times 10^{-3}$); (**E**, EdU (Boundary)): *en > p35, rod-i* ($n = 8$) vs *en > p35, rod-i, wg-i* ($n = 9$) ($p = 4.66 \times 10^{-5}$). (**C**) Volcano plot representing selected genes in 'Wnt Signalling - GO0060070 and GO0090090' classes that are, differentially upregulated (red area, FC > 1.3 and p-value < 0.05), downregulated (blue area, FC < −1.3 and p-value < 0.05), or not differentially expressed (grey area, p-value > 0.05 or 1.3 > FC > −1.3) in aneuploid cells when compared to P cells of control discs. (**D**) Wing discs subjected to CIN, stained to visualize TUNEL EdU (red or white), DAPI (blue), Ci (green), and expressing the indicated transgenes in the P compartment under the control of the *en-gal4* driver. High magnifications of the squared regions are shown in the lower panels. Note that the EdU incorporation of the anterior stripe abutting the P compartment in CIN tissues, demarcated by two green lines and double-headed arrow, is not observed upon targeted depletion of *wingless* in the P compartment. In (**A**, **D**), scale bars, 50 µm and discs were dissected 5 days AEL. See also Dataset EV3.

 © The Author(s)

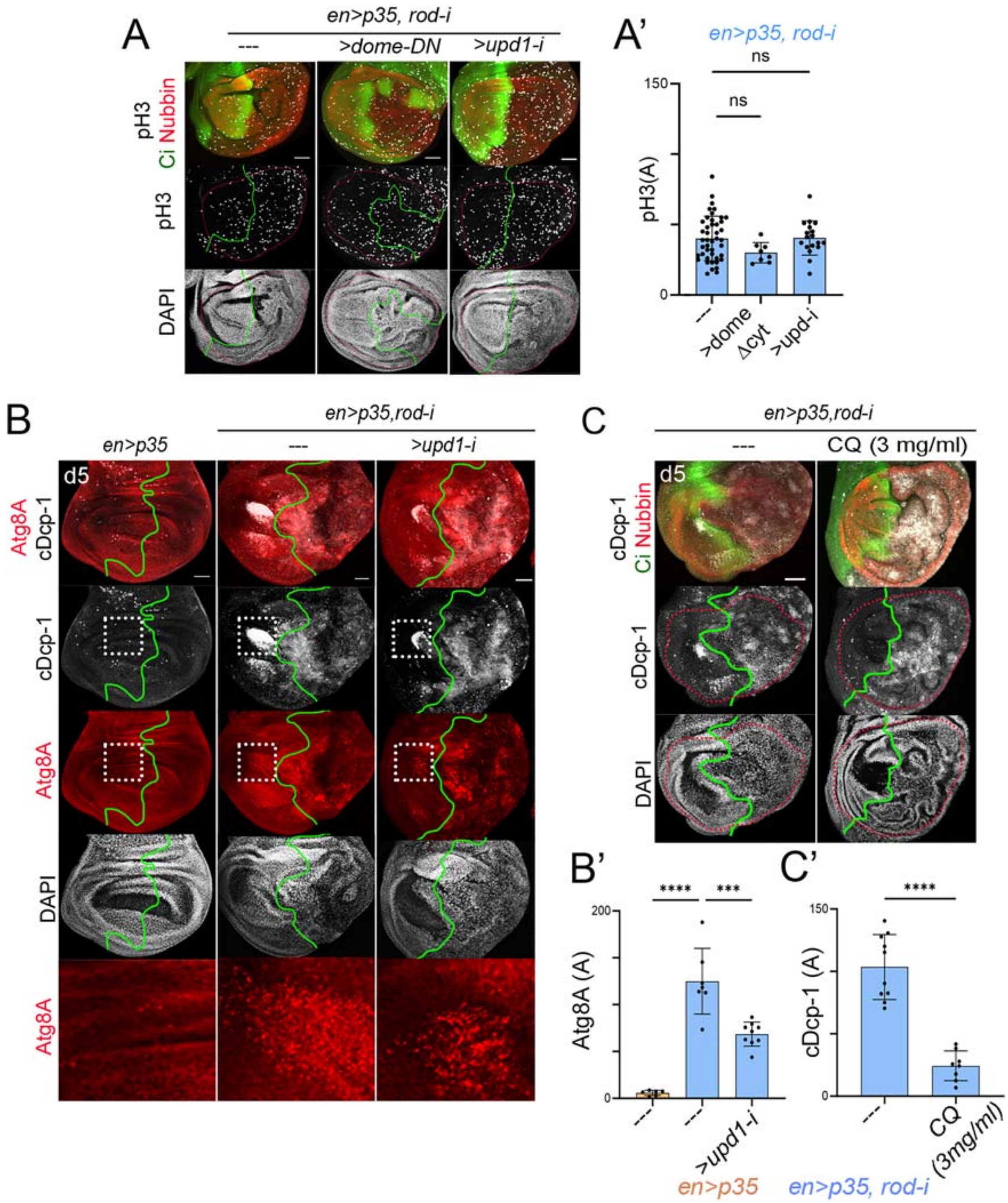

Figure EV6. Contribution of Upd cytokines to the non-autonomous effects of CIN tumours on cell proliferation (related to Fig. 7).

(**A–C**) Control wing discs (first column in **A–C**) and wing discs subjected to CIN (remaining columns) stained for pH3 (white, **A**), cDcp-1 (red and white, **B**; white, **C**), mCherry-Atg8 (red, **B**), –Nubbin (red, to label the wing pouch cells, **A**, **C**), and Ci (green, to label the A compartment, **A**, **C**), and expressing the indicated transgenes in the P compartment under the control of the *en-gal4* driver. Larvae in **C** were subjected to chloroquine (CQ) feeding for 72 h. High magnification of the squared regions in (**B**) are shown in the lower panels. Scale bars, 50 µm. Discs were dissected 5 days AEL. The boundary between A and P cells is labelled by a green line. (**A′–C′**) Histograms plotting the amount of pH3 signal (**A′**), mCherry-Atg8 signal (**B′**) or cDcp-1 signal (**C′**) in the A wing pouch compartment (normalized to the size of the corresponding compartment) of wing discs expressing the indicated transgenes in the P compartment under the control of the *en-gal4* driver. Graphs represent mean and standard deviation. **p* < 0.05, ***p* < 0.01, ****p* < 0.001, *****p* < 0.0001, ns, not significant. One-way ANOVA with Šídák's multiple comparisons test (**A′**, **B′**), Unpaired t-test (**C′**), *n* = number of biological replicates. Sample size and *p* value: (**A′**, pH3 (A)): *en > p35, rod-i* ($n = 44$) vs *en > p35, rod-i, dome-Δcyt* ($n = 8$) ($p = 0.181$); and *en > p35, rod-i* ($n = 44$) *vs en > p35, rod-i, upd1-i* ($n = 18$) ($p = 0.99$); (**B′**, Atg8 (A)): *en > p35* ($n = 6$) vs *en > p35, rod-i* ($n = 7$) ($p = 1.07 \times 10^{-8}$); and *en > p35, rod-i* ($n = 7$) *vs en > p35, rod-i, upd1-i* ($n = 9$) ($p = 9.70 \times 10^{-5}$); (**C′**, cDcp1 (A)): *en > p35, rod-i* ($n = 10$) *vs CQ treated en > p35, rod-i* ($n = 8$) ($p = 6.24 \times 10^{-7}$). See also Dataset EV3.
