# Supplementary material for: A tumour-host feed-forward loop contributes to the growth of chromosomal instability-induced tumours
Source: EMBO Rep. 2026 Jun 5;27(16):4694–720. doi: 10.1038/s44319-026-00811-7 (PMC13503916; doi:10.1038/s44319-026-00811-7)
Supplement: Supplementary file 2 [file 44319_2026_811_MOESM1_ESM.pdf]

EMBO reports

# A tumour-host feed-forward loop contributes to the growth of chromosomal instability-induced tumours

Kaustuv Ghosh, Aishwarya Kunchur, and Marco Milan

*Corresponding author(s): Marco Milan (marco.milan@irbbarcelona.org)*



## Review Timeline:

| | |
|---|---|
| Transfer Date: | 23rd Mar 26 |
| Editorial Decision: | 9th Apr 26 |
| Revision Received: | 24th Apr 26 |
| Editorial Decision: | 6th May 26 |
| Revision Received: | 11th May 26 |
| Accepted: | 20th May 26 |

*Editor: Achim Breiling*

## Transaction Report: This manuscript was transferred to EMBO Reports after peer review at another journal.

(Note: With the exception of the correction of typographical or spelling errors that could be a source of ambiguity, letters and reports are not edited. Depending on transfer agreements, referee reports obtained elsewhere may or may not be included in this compilation. Referee reports are anonymous unless the Referee chooses to sign their reports.)

Dear Prof. Milan,

Thank you for the transfer of your revised manuscript to EMBO reports. I have now received the reports from the arbitrator that I asked to evaluate the study, you will find below. As you will see, the arbitrator supports the publication of the study in EMBO reports.

The arbitrator has some minor points and suggestions I ask you to address in a final revised manuscript. Please provide a p-b-p-response to the arbitration report. The manuscript now needs also formatting according to our journal style. Please carefully review the instructions that follow below.

PLEASE NOTE THAT upon resubmission revised manuscripts are subjected to an initial quality control prior to exposition to re-review. Upon failure in the initial quality control, the manuscripts are sent back to the authors, which may lead to delays. Frequent reasons for such a failure are the lack of the data availability section (please see below) and the presence of statistics based on n=2 (the authors are then asked to present scatter plots or provide more data points).

When submitting your revised manuscript, we will require:

1) a .docx formatted version of the final manuscript text (including legends for main figures, EV figures and tables), but without the figures included. Figure legends should be compiled at the end of the manuscript text.

2) individual production quality figure files as .eps, .tif, .jpg (one file per figure), of main figures and EV figures. Please upload these as separate, individual files upon re-submission.

The Expanded View format, which will be displayed in the main HTML of the paper in a collapsible format, has replaced the Supplementary information. You can submit up to 6 images as Expanded View. Please follow the nomenclature Figure EV1, Figure EV2 etc. The figure legend for these should be included in the main manuscript document file in a section called Expanded View Figure Legends after the main Figure Legends section. Additional Supplementary material should be supplied as a single pdf file labeled Appendix. The Appendix should have page numbers and needs to include a table of content on the first page (with page numbers) and legends for all content. Please follow the nomenclature Appendix Figure Sx, Appendix Table Sx etc. throughout the text, and also label the figures and tables according to this nomenclature.

For more details, please refer to our guide to authors:
https://link.springer.com/journal/44319/submission-guidelines#cms-Revised-submissions

Please consult our guide for figure preparation:
https://media.springernature.com/original/springer-cms/rest/v1/content/27825798/data/v1
3) a complete author checklist, which you can download from our author guidelines (https://link.springer.com/journal/44319/submission-guidelines#cms-Revised-submissions). Please insert page numbers in the checklist to indicate where the requested information can be found in the manuscript. The completed author checklist will also be part of the RPF.

Please also follow our guidelines for the use of living organisms, and the respective reporting guidelines:
https://link.springer.com/partners/embo-press/editorial-policies#Animal%20and%20human%20studies

4) that primary datasets produced in this study (e.g. RNA-seq, ChIP-seq, structural and array data) are deposited in an appropriate public database. If no primary datasets have been deposited, please also state this in a dedicated section (e.g. 'No primary datasets have been generated and deposited'), see below.

See also: https://link.springer.com/partners/embo-press/editorial-policies#Data%20deposition

Please remember to provide a reviewer password if the datasets are not yet public.

The accession numbers and database should be listed in a formal "Data Availability" section that follows the model below. This is now mandatory (like the COI statement). Please note that the Data Availability Section is restricted to new primary data that are part of this study. This section is mandatory. As indicated above, if no primary datasets have been deposited, please state this in this section

# Data availability

The datasets produced in this study are available in the following databases:

- RNA-Seq data: Gene Expression Omnibus GSE46843 (https://www.ncbi.nlm.nih.gov/geo/query/acc.cgi?acc=GSE46843)

- [data type]: [name of the resource] [accession number/identifier/doi] ([URL or identifiers.org/DATABASE:ACCESSION])

*** Note - All links should resolve to a page where the data can be accessed. ***

See also: https://link.springer.com/partners/embo-press/editorial-policies#Data%20availability%20statement

Moreover, I have these editorial requests:

5) We now request the publication of original source data with the aim of making primary data more accessible and transparent to the reader. You will receive a separate email with instructions for providing source data with your revised manuscript, including information how to upload and organize the files.

6) Our journal encourages inclusion of *data citations in the reference list* to directly cite datasets that were re-used and obtained from public databases. Data citations in the article text are distinct from normal bibliographical citations and should directly link to the database records from which the data can be accessed. In the main text, data citations are formatted as follows: "Data ref: Smith et al, 2001" or "Data ref: NCBI Sequence Read Archive PRJNA342805, 2017". In the Reference list, data citations must be labeled with "[DATASET]". A data reference must provide the database name, accession number/identifiers and a resolvable link to the landing page from which the data can be accessed at the end of the reference. Further instructions are available at: https://link.springer.com/journal/44319/submission-guidelines#cms-Reference-guidelines

7) Regarding data quantification and statistics, please make sure that the number "n" for how many independent experiments were performed, their nature (biological versus technical replicates), the bars and error bars (e.g. SEM, SD) and the test used to calculate p-values is indicated in the respective figure legends (also for EV and Appendix figures). Please also check that all the p-values are explained in the legend, and that these fit to those shown in the figure. Please provide statistical testing where applicable. Please avoid the phrase 'independent experiment' but clearly state if these were biological or technical replicates. Please also indicate (e.g. with n.s.) if testing was performed, but the differences are not significant. In case n=2, please show the data as separate datapoints without error bars and statistics. See also: https://link.springer.com/journal/44319/submission-guidelines#cms-Figure-and-data-presentation

If n<5, please show single datapoints for diagrams.

8) Please add scale bars of similar style and thickness to all microscopic images, using clearly visible black or white bars (depending on the background). Please place these in the lower right corner of the images themselves. Please do not write on or near the bars in the image but define the size in the respective figure legend.

9) Please also note our reference format:
https://link.springer.com/journal/44319/submission-guidelines#cms-Reference-guidelines

10) We updated our journal's competing interests policy in January 2022 and request authors to consider both actual and perceived competing interests. Please review the policy https://www.embopress.org/competing-interests and update your competing interests if necessary. Please name this section 'Disclosure and Competing Interests Statement' and put it after the Acknowledgements section.
See: https://link.springer.com/partners/embo-press/editorial-policies#Competing%20interest%20disclosures

11) We now use CRediT to specify the contributions of each author in the journal submission system. CRediT replaces the author contribution section. Please use the free text box to provide more detailed descriptions and do NOT provide your final manuscript text file with an author contributions section. See also our guide to authors (section 'Author contributions'): https://link.springer.com/journal/44319/submission-guidelines#cms-Revised-submissions

12) All Materials and Methods need to be described in the main text using our 'Structured Methods' format, which is required for all research articles. According to this format, the Methods section should include a Reagents and Tools Table (listing key reagents, experimental models, software, and relevant equipment and including their sources and relevant identifiers), uploaded as separate file, and a Methods section in which we encourage the authors to describe their methods using a step-by-step protocol format with bullet points, to facilitate the adoption of the methodologies across labs. More information on how to adhere to this format as well as downloadable templates (.doc) for the Reagents and Tools Table can be found in our author guidelines (section 'Structured Methods'):

https://link.springer.com/journal/44319/submission-guidelines#cms-Manuscript-organisation-and-formatting

Please provide all antibody or primer information in the Reagents & Tools Table.

13) Please order the manuscript sections like this, using only these names:
Title page - Abstract (max. 175 words) - Keywords (up to five) - Introduction - Results - Discussion - Methods - Data availability section - Acknowledgements (please include here all the funding information) - Disclosure and Competing Interests Statement -

References - Figure legends - Expanded View Figure legends

14) Please make sure that all the funding information is also entered into the online submission system and that it is complete and similar to the one in the acknowledgement section of the manuscript text file.

In addition, I would need from you:
- a short, two-sentence summary of the manuscript (not more than 35 words).
- three to four short (!) one sentence bullet points highlighting the key findings of your study.
- a schematic summary figure (synopsis image) in jpeg or tiff format with the exact width of 550 pixels and a height of exactly 300 pixels that can be used as a visual synopsis on our website.

We would also welcome the submission of cover suggestions, or motifs to be used by our Graphics Illustrator in designing a cover.

As part of the EMBO publication's Transparent Editorial Process, EMBO reports publishes online a Review Process File (RPF) to accompany accepted manuscripts. This File will be published in conjunction with your paper and will include the referee reports, your point-by-point response and all pertinent correspondence relating to the manuscript.

You are able to opt out of this by letting the editorial office know (emboreports@embo.org). If you do opt out, the Review Process File link will point to the following statement: "No Review Process File is available with this article, as the authors have chosen not to make the review process public in this case."

I look forward to seeing a revised form of your manuscript when it is ready.

Please let me know if you have questions or comments regarding the revision.

Yours sincerely,

Achim Breiling
Senior Editor
EMBO Reports

---------------
Arbitrator:

The Drosophila wing disc has proved a powerful system in which to study the mechanisms of regeneration and the response to aneuploidy in a mitotically active epithelial tissue. In both cases, a powerful growth response is orchestrated by a population of senescent cells with a Senescence-Associated Secretory Phenotype (SASP). Despite its relevance to mammalian tumour behaviour, the SASP response to chromosomal instability has not been thoroughly explored. The authors performed a careful transcriptional comparison aneuploidy-induced SASP cells, their proliferative non-aneuploid neighbours, and cells from the neighbouring non-CIN compartment, showing that the seemingly chaotic response to aneuploidy is underpinned by a remarkably ordered transcriptional programme. They then go on to rigorously dissect the cell-autonomous (secretory phenotype, protection of SASP cells from cell death by Yki activation), tissue autonomous (Wg secretion, death of non-CIN cells fuelling tumour growth via cell competition) and systemic (Dilp8 and ImpL2 secretion) consequences of aneuploidy-induced SASP. The manuscript provides a holistic understanding of the different levels of crosstalk between aneuploid cells and their neighbours, and will therefore be of interest to many researchers interested in growth in developmental, regenerative and pathological contexts.

Looking through the previous reviewers' comments, I find that the manuscript has been much improved by the revision process, both in terms of the mechanistic questions of the reviewers, the clarity of the presentation and the relation of the work with mammalian cancer. I am happy to recommend publication in EMBO reports and have a few very minor changes for the authors to consider.

Minor comments:
1. "Diap1 (an E3 ubiquitin ligase regulated by Yorkie and involved in inhibiting Dronc...)
Note that Diap1 has also been reported to antagonise the effector caspases Drice and Dcp-1 via sequestration rather than catalytic inactivation (see for example PMID: 15580265).

2. "Since experimental manipulation of Dilp8 levels in the P compartment was unable to have a significant effect on the size of the nearby A compartment (Figure 6D)...". This statement is confusing because there are four asterisks above the dilp8-i column, although it is not specified in the legend what comparison this refers to. To allow the reader to follow the logical flow of the text without going through all the statistics table S3, I suggest the authors put horizontal lines specifying the comparisons to which the asterisks refer to, as they have done for Figure 6B', C' and E. At least if a comparison is referred to in the main text, it

should appear in the figure, even if there is no space to put all possible combinations individually on the panel.

3. "However, the apoptotic pathway is not completely blocked under these conditions as shown by the high activity levels of effector caspases in the tissue, which have been shown to contribute in a non-apoptotic manner to the invasive behaviour of aneuploid cells." Please add references for this statement.

Arbitrator:

We appreciate Arbitrator's support of our ms and we are very thankful for his/her thorough analysis of the paper, reviewers' comments, responses and corresponding changes in the ms.

The Drosophila wing disc has proved a powerful system in which to study the mechanisms of regeneration and the response to aneuploidy in a mitotically active epithelial tissue. In both cases, a powerful growth response is orchestrated by a population of senescent cells with a Senescence-Associated Secretory Phenotype (SASP). Despite its relevance to mammalian tumour behaviour, the SASP response to chromosomal instability has not been thoroughly explored. The authors performed a careful transcriptional comparison aneuploidy-induced SASP cells, their proliferative non-aneuploid neighbours, and cells from the neighbouring non-CIN compartment, showing that the seemingly chaotic response to aneuploidy is underpinned by a remarkably ordered transcriptional programme. They then go on to rigorously dissect the cell-autonomous (secretory phenotype, protection of SASP cells from cell death by Yki activation), tissue autonomous (Wg secretion, death of non-CIN cells fuelling tumour growth via cell competition) and systemic (Dilp8 and ImpL2 secretion) consequences of aneuploidy-induced SASP. The manuscript provides a holistic understanding of the different levels of crosstalk between aneuploid cells and their neighbours, and will therefore be of interest to many researchers interested in growth in developmental, regenerative and pathological contexts.

*Looking through the previous reviewers' comments, I find that the manuscript has been much improved by the revision process, both in terms of the mechanistic questions of the reviewers, the clarity of the presentation and the relation of the work with mammalian cancer. I am happy to recommend publication in EMBO reports and have a few very minor changes for the authors to consider.*

We appreciate Reviewer's support of our ms.

Minor comments:

*1. "Diap1 (an E3 ubiquitin ligase regulated by Yorkie and involved in inhibiting Dronc...)*

*Note that Diap1 has also been reported to antagonise the effector caspases Drice and Dcp-1 via sequestration rather than catalytic inactivation (see for example PMID: 15580265).*

The suggested reference has been included.

*2. "Since experimental manipulation of Dilp8 levels in the P compartment was unable to have a significant effect on the size of the nearby A compartment (Figure 6D)...".*

*This statement is confusing because there are four asterisks above the dilp8-i column, although it is not specified in the legend what comparison this refers to. To allow the reader to follow the logical flow of the text without going through all the statistics table S3, I suggest the authors put horizontal lines specifying the comparisons to which the asterisks refer to, as they have done for Figure 6B', C' and E. At least if a comparison is referred to in the main text, it should appear in the figure, even if there is no space to put all possible combinations individually on the panel.*

As suggested, we have added horizontal lines in all figures to mark the comparisons associated to the indicated p values.

*3. "However, the apoptotic pathway is not completely blocked under these conditions as shown by the high activity levels of effector caspases in the tissue, which have been shown to contribute in a non-apoptotic manner to the invasive behaviour of aneuploid cells." Please add references for this statement.*

As suggested, we have added a reference to this sentence (Barrio et al, 2023).

## 1st Revision - Editorial Decision **6th May 2026**

Dear Prof. Milan,

Thank you for the submission of your further revised manuscript to our editorial offices. I now went through this and your final p-b-p-response and consider the points of the arbitrator as adequately addressed.

Before we can proceed with formal acceptance, I have the editorial requests below I ask you to address in a final revised manuscript. Please also provide a final p-b-p-response to the editorial requests.

Editorial requests:

- On the title page a lead contact and a corresponding author is indicated. Though none of the authors is marked as lead contact (4). If the lead contact and the corresponding author are identical, please just indicate the corresponding author ('Correspondence should be addressed to: ...').

- Please remove the running title, number of figures, number of tables, number of characters and the author ORCIDs from the title page. Please make sure that the ORCIDs are linked to the author names in the submission system.

- Please check again that the number "n" for how many independent experiments were performed, their nature (biological versus technical replicates), the bars and error bars (e.g. SEM, SD) and the test used to calculate p-values is indicated in the respective figure legends (main and EV figures). Please also check that all the p-values are explained in the legend, and that this fit to those shown in the figure. Please provide statistical testing where applicable. Please avoid the phrase 'independent experiment' but clearly state if these were biological or technical replicates. Please also indicate (e.g. with n.s.) if testing was performed, but the differences are not significant. In case n=2, please show the data as separate datapoints without error bars and statistics. See also:

https://link.springer.com/journal/44319/submission-guidelines#cms-Figure-and-data-presentation

If n<5, please show single datapoints for diagrams. Moreover:
- Please note that the legends for figures 1, 4, 5, 6, EV-5; are not provided in the sequential manner (legend for figure 1G is provided before legend of figure 1F; legend for figure 4C is provided before legend of figure 4B; legend for figure 5D,E are provided before legend of figure 5B,C; legend for figure 6F is provided before legend of figure 6E; legend for figure EV-5D is provided before legend of figure EV-5C). This needs to be rectified.
- Please note that the exact p values are not provided in the legends of 3D',E'; 4B,D,I; 5B,C; 6B'C'D,E; 7D'E,F; EV-5B,E; Appendix Figures S1-A'B'.
- Please indicate the statistical test used for data analysis in the legends of figures 1E,F,G; 2A,B; 3A; EV-2A,B; EV-4A,B.
- Please note that information related to n is missing in the legends of 1F; 2A,B; 3A; 7A; EV-4A.

- Please remove now the referee access (token) from the data availability section and add a final direct links to the dataset. Please make sure the dataset is public latest upon online publication of the paper.

- Please make sure that all the funding information is also entered into the online submission system and that it is complete and similar to the one in the acknowledgement section of the manuscript text file. Presently, the institutional funding from the MICIU through the Centres of Excellence Severo Ochoa Award and from the CERCA Programme of the Catalan Government is not listed in the submission system. Please check.

- Tables EV1 and EV2 are called out in the manuscript text but are not uploaded. Please check.

- Table 1 seems also to be part of Appendix Table S2. Please decide where this data should be shown. If this remains Table 1, please add the table to the main manuscript text file with its legend between the main and EV figure legends (and remove the TAB from Table S2). Check that the callouts are correct.

- Please name the main figure legend just 'Figure Legends'.

- Tables S1, S2 and S3 are datasets. Please upload these as dataset files using the nomenclature 'Dataset EVx' and with their legends on the first TAB of the excel file. Please update all callouts and remove their legends form the Appendix file.

- Please add the data shown in Appendix Figure S1 to the main or EV figures. Then, we do not need an Appendix. Finally, please update the callouts and remove the Appendix from the submission.

- Please upload the Reagents & Tools Table only as separate file and remove the table from the manuscript text file. Please add callouts to the table where appropriate.

I look forward to seeing the further revised version of your manuscript when it is ready. Please let me know if you have questions regarding the revision.

Best,

Achim Breiling
Senior Editor
EMBO Reports

The authors have addressed all minor editorial requests.

Prof. Marco Milan
IRB Barcelona
Mechanisms of Disease Programme
Baldiri i Reixac, 10
Barcelona 08028
Spain

Dear Prof. Milan,

I am very pleased to accept your manuscript for publication in the next available issue of EMBO reports. Thank you for your contribution to our journal.

Your manuscript will be processed for publication by EMBO Press. It will be copy edited and you will receive page proofs prior to publication. Please note that you will be contacted by Springer Nature Author Services to complete licensing and payment information.

You may qualify for financial assistance for your publication charges - either via a Springer Nature fully open access agreement or an EMBO initiative. Check your eligibility: https://link.springer.com/journal/44319/how-to-publish-with-us

Should you be planning a Press Release on your article, please get in contact with embo_production@springernature.com as early as possible in order to coordinate publication and release dates.

If you have any questions, please do not hesitate to contact the Editorial Office. Thank you for your contribution to EMBO Reports.

Yours sincerely,

Achim Breiling
Senior Editor
EMBO Reports

------------------------------------------------

>>> Please note that it is EMBO Reports policy for the transcript of the editorial process (containing referee reports and your response letter) to be published as an online supplement to each paper. If you do NOT want this, you will need to inform the Editorial Office via email immediately. More information is available here: https://link.springer.com/partners/embo-press/editorial-policies#Peer%20review

## EMBO Press Author Checklist

| | |
|---|---|
| Corresponding Author Name: Marcpo Milán | |
| Journal Submitted to: EMBO Reports | |
| Manuscript Number: EMBOR-2026-64088-T | |

**USEFUL LINKS FOR COMPLETING THIS FORM**
The EMBO Journal - Author Guidelines
EMBO Reports - Author Guidelines
Molecular Systems Biology - Author Guidelines
EMBO Molecular Medicine - Author Guidelines

**Reporting Checklist for Life Science Articles (updated January**
This checklist is adapted from Materials Design Analysis Reporting (MDAR) Checklist for Authors. MDAR establishes a minimum set of requirements in transparent reporting in the life sciences (see Statement of Task: 10.31222/osf.io/9sm4x). Please follow the journal's guidelines in preparing your
**Please note that a copy of this checklist will be published alongside your article.**

**Abridged guidelines for figures**
**1. Data**
The data shown in figures should satisfy the following conditions:
- the data were obtained and processed according to the field's best practice and are presented to reflect the results of the experiments in an accurate and unbiased manner.
- ideally, figure panels should include only measurements that are directly comparable to each other and obtained with the same assay.
- plots include clearly labeled error bars for independent experiments and sample sizes. Unless justified, error bars should not be shown for technical
- if n<5, the individual data points from each experiment should be plotted. Any statistical test employed should be justified.
- Source Data should be included to report the data underlying figures according to the guidelines set out in the authorship guidelines on Data

**2. Captions**
Each figure caption should contain the following information, for each panel where they are relevant:
- a specification of the experimental system investigated (eg cell line, species name).
- the assay(s) and method(s) used to carry out the reported observations and measurements.
- an explicit mention of the biological and chemical entity(ies) that are being measured.
- an explicit mention of the biological and chemical entity(ies) that are altered/varied/perturbed in a controlled manner.
- the exact sample size (n) for each experimental group/condition, given as a number, not a range;
- a description of the sample collection allowing the reader to understand whether the samples represent technical or biological replicates (including how many animals, litters, cultures, etc.).
- a statement of how many times the experiment shown was independently replicated in the laboratory.
- definitions of statistical methods and measures:
  - common tests, such as t-test (please specify whether paired vs. unpaired), simple χ2 tests, Wilcoxon and Mann-Whitney tests, can be unambiguously identified by name only, but more complex techniques should be described in the methods section;
  - are tests one-sided or two-sided?
  - are there adjustments for multiple comparisons?
  - exact statistical test results, e.g., P values = x but not P values < x;
  - definition of 'center values' as median or average;
  - definition of error bars as s.d. or s.e.m.

**Please complete ALL of the questions below.**
**Select "Not Applicable" only when the requested information is not relevant for your study.**

**Materials**

| Newly Created Materials | Information included in the manuscript? | In which section is the information available? (Reagents and Tools Table, Materials and Methods, Figures, Data Availability Section) |
|---|---|---|
| New materials and reagents need to be available; do any restrictions apply? | Not Applicable | |

| Antibodies | Information included in the manuscript? | In which section is the information available? (Reagents and Tools Table, Materials and Methods, Figures, Data Availability Section) |
|---|---|---|
| For **antibodies** provide the following information:<br>- Commercial antibodies: RRID (if possible) or supplier name, catalogue number and or/clone number<br>- Non-commercial: RRID or citation | Yes | Reagents and Tools Table |

| DNA and RNA sequences | Information included in the manuscript? | In which section is the information available? (Reagents and Tools Table, Materials and Methods, Figures, Data Availability Section) |
|---|---|---|
| **Short novel DNA or RNA including primers, probes**: provide the sequences. | Not Applicable | |

| Cell materials | Information included in the manuscript? | In which section is the information available? (Reagents and Tools Table, Materials and Methods, Figures, Data Availability Section) |
|---|---|---|
| **Cell lines:** Provide species information, strain. Provide accession number in repository **OR** supplier name, catalog number, clone number, and/**OR** RRID. | Not Applicable | |
| **Primary cultures:** Provide species, strain, sex of origin, genetic modification status. | Not Applicable | |
| Report if the cell lines were recently **authenticated** (e.g., by STR profiling) and tested for mycoplasma contamination. | Not Applicable | |

| Experimental animals | Information included in the manuscript? | In which section is the information available? (Reagents and Tools Table, Materials and Methods, Figures, Data Availability Section) |
|---|---|---|
| **Laboratory animals or Model organisms:** Provide species, strain, sex, age, genetic modification status. Provide accession number in repository **OR** supplier name, catalog number, clone number, **OR** RRID. | Yes | Reagents and Tools Table + Fly maintenance, husbandry and transgene expression (Methods section) |
| **Animal observed in or captured from the field:** Provide species, sex, and age where possible. | Not Applicable | |
| Please detail **housing and husbandry conditions**. | Yes | Fly maintenance, husbandry and transgene expression (Methods section) |

| Plants and microbes | Information included in the manuscript? | In which section is the information available? (Reagents and Tools Table, Materials and Methods, Figures, Data Availability Section) |
|---|---|---|
| **Plants:** provide species and strain, ecotype and cultivar where relevant, unique accession number if available, and source (including location for collected wild specimens). | Not Applicable | |
| **Microbes:** provide species and strain, unique accession number if available, and source. | Not Applicable | |

| Human research participants | Information included in the manuscript? | In which section is the information available? (Reagents and Tools Table, Materials and Methods, Figures, Data Availability Section) |
|---|---|---|
| If collected and within the bounds of privacy constraints report on age, sex and gender or ethnicity for all study participants. | Not Applicable | |

| Core facilities | Information included in the manuscript? | In which section is the information available? (Reagents and Tools Table, Materials and Methods, Figures, Data Availability Section) |
|---|---|---|
| If your work benefited from core facilities, was their service mentioned in the acknowledgments section? | Yes | Acknowledgments |

**Design**

| **Study protocol** | **Information included in the manuscript?** | **In which section is the information available?** (Reagents and Tools Table, Materials and Methods, Figures, Data Availability Section) |
|---|---|---|
| If study protocol has been **pre-registered, provide DOI in the manuscript**. For clinical trials, provide the trial registration number **OR** cite DOI. | Not Applicable | |
| Report the **clinical trial registration number** (at ClinicalTrials.gov or equivalent), where applicable. | Not Applicable | |

| **Laboratory protocol** | **Information included in the manuscript?** | **In which section is the information available?** (Reagents and Tools Table, Materials and Methods, Figures, Data Availability Section) |
|---|---|---|
| Provide DOI OR other citation details if **external detailed step-by-step protocols** are available. | Not Applicable | |

| **Experimental study design and statistics** | **Information included in the manuscript?** | **In which section is the information available?** (Reagents and Tools Table, Materials and Methods, Figures, Data Availability Section) |
|---|---|---|
| Include a statement about **sample size** estimate even if no statistical methods were used. | Yes | Sample size was always bigger than 5 |
| Were any steps taken to minimize the effects of subjective bias when allocating animals/samples to treatment (e.g. **randomization procedure**)? If yes, have they been described? | Yes | The sex of experimental larvae was not considered relevant to this study and was not determined |
| Include a statement about **blinding** even if no blinding was done. | Yes | No blinding was done. |
| Describe **inclusion/exclusion criteria** if samples or animals were excluded from the analysis. Were the criteria pre-established?<br><br>If sample or data points were omitted from analysis, report if this was due to attrition or intentional exclusion and provide justification. | Not Applicable | |
| For every figure, are **statistical tests** justified as appropriate? Do the data meet the assumptions of the tests (e.g., normal distribution)? Describe any methods used to assess it. Is there an estimate of variation within each group of data? Is the variance similar between the groups that are being statistically compared? | Yes | Figure legends and Statistical analysis (Methods section) |

| **Sample definition and in-laboratory replication** | **Information included in the manuscript?** | **In which section is the information available?** (Reagents and Tools Table, Materials and Methods, Figures, Data Availability Section) |
|---|---|---|
| In the figure legends: state number of times the experiment was **replicated** in laboratory. | Yes | Figure legends |
| In the figure legends: define whether data describe **technical or biological replicates**. | Yes | Figure legends |

## Ethics

| **Ethics** | **Information included in the manuscript?** | **In which section is the information available?** (Reagents and Tools Table, Materials and Methods, Figures, Data Availability Section) |
|---|---|---|
| Studies involving **human participants**: State details of **authority granting ethics approval** (IRB or equivalent committee(s), provide reference number for approval. | Not Applicable | |
| Studies involving **human participants**: Include a statement confirming that **informed consent** was obtained from all subjects and that the experiments conformed to the principles set out in the WMA Declaration of Helsinki and the Department of Health and Human Services Belmont Report. | Not Applicable | |
| Studies involving **human participants:** For publication of **patient photos**, include a statement confirming that consent to publish was obtained. | Not Applicable | |
| Studies involving experimental **animals**: State details of **authority granting ethics approval** (IRB or equivalent committee(s), provide reference number for approval. Include a statement of compliance with ethical regulations. | Not Applicable | |
| Studies involving **specimen and field samples:** State if relevant **permits** obtained, provide details of authority approving study; if none were required, explain why. | Not Applicable | |

| **Dual Use Research of Concern (DURC)** | **Information included in the manuscript?** | **In which section is the information available?** (Reagents and Tools Table, Materials and Methods, Figures, Data Availability Section) |
|---|---|---|
| Could your study fall under dual use research restrictions? Please check biosecurity documents and list of **select agents and toxins** (CDC): https://www.selectagents.gov/sat/list.htm | Not Applicable | |
| If you used a select agent, is the security level of the lab appropriate and reported in the manuscript? | Not Applicable | |
| If a study is subject to dual use research of concern regulations, is the name of the **authority granting approval and reference number** for the regulatory approval provided in the manuscript? | Not Applicable | |

## Reporting

The MDAR framework recommends adoption of discipline-specific guidelines, established and endorsed through community initiatives. Journals have their own policy about requiring specific guidelines and recommendations to complement MDAR.

| **Adherence to community standards** | **Information included in the manuscript?** | **In which section is the information available?** (Reagents and Tools Table, Materials and Methods, Figures, Data Availability Section) |
|---|---|---|
| State if relevant guidelines or checklists (e.g., **ICMJE, MIBBI, ARRIVE, PRISMA**) have been followed or provided. | Not Applicable | |
| For **tumor marker prognostic studies,** we recommend that you follow the **REMARK** reporting guidelines (see link list at top right). See author guidelines, under 'Reporting Guidelines'. Please confirm you have followed these guidelines. | Not Applicable | |
| For **phase II and III randomized controlled trials**, please refer to the **CONSORT** flow diagram (see link list at top right) and submit the CONSORT checklist (see link list at top right) with your submission. See author guidelines, under 'Reporting Guidelines'. Please confirm you have submitted this list. | Not Applicable | |

## Data Availability

| **Data availability** | **Information included in the manuscript?** | **In which section is the information available?** (Reagents and Tools Table, Materials and Methods, Figures, Data Availability Section) |
|---|---|---|
| Have **primary datasets** been deposited according to the journal's guidelines (see 'Data Deposition' section) and the respective accession numbers provided in the Data Availability Section? | Yes | Data availability (Methods section) |
| Were **human clinical and genomic datasets** deposited in a public access-controlled repository in accordance to ethical obligations to the patients and to the applicable consent agreement? | Not Applicable | |
| Are **computational models** that are central and integral to a study available without restrictions in a machine-readable form? Were the relevant accession numbers or links provided? | Not Applicable | |
| If publicly available data were reused, provide the respective **data citations in the reference list**. | Not Applicable | |
