# Supplementary material for: A tumour-host feed-forward loop contributes to the growth of chromosomal instability-induced tumours
Source: EMBO Rep. 2026 Jun 5;27(16):4694–720. doi: 10.1038/s44319-026-00811-7 (PMC13503916; doi:10.1038/s44319-026-00811-7)
Supplement: Supplementary file 5 [file 44319_2026_811_MOESM5_ESM.zip › Figure 1/Figure 1C/pca.pdf]

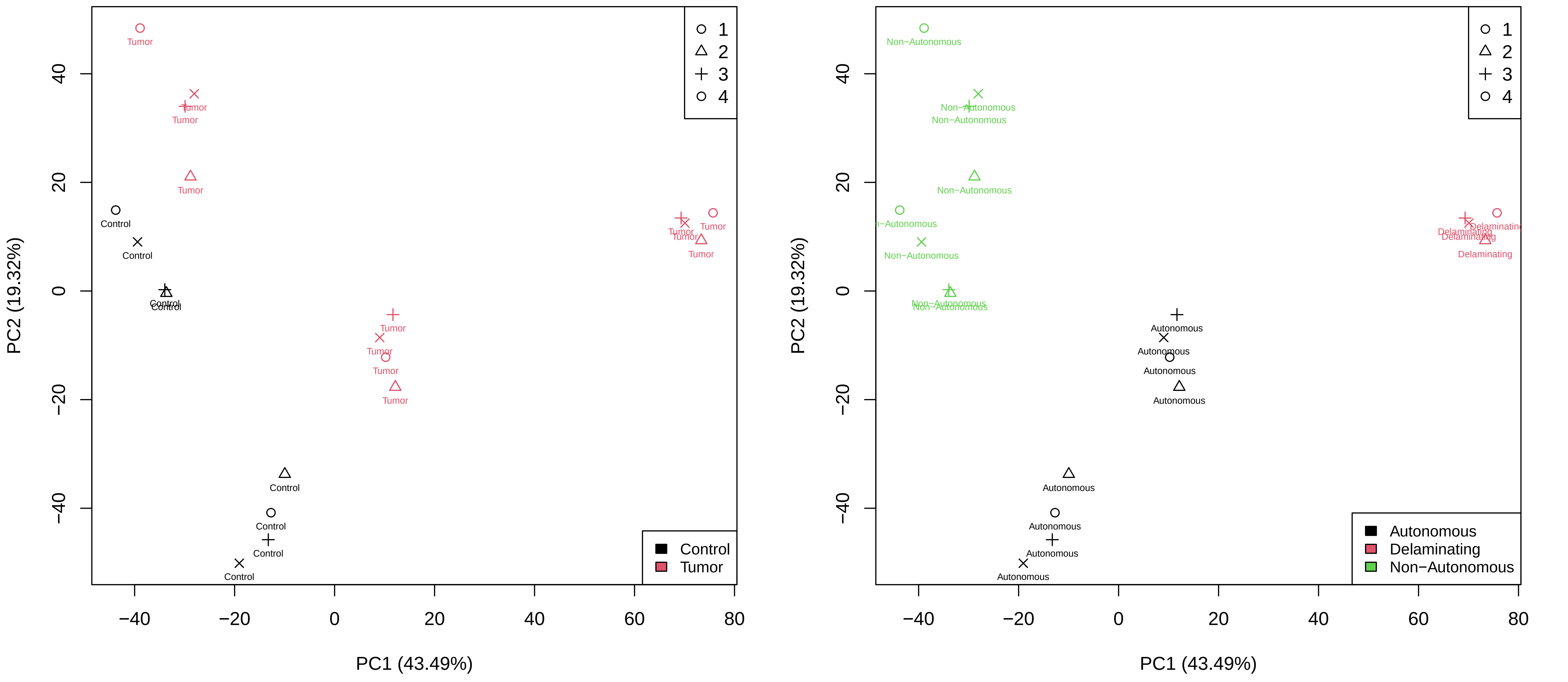

PC1 (43.49%)
PC2 (19.32%)
1
2
3
4
Control
Tumor
Autonomous
Delaminating
Non−Autonomous
